# Expression profile analysis identifies IER3 to predict overall survival and promote lymph node metastasis in tongue cancer

**DOI:** 10.1186/s12935-019-1028-2

**Published:** 2019-11-21

**Authors:** Fang Xiao, Yinhua Dai, Yujiao Hu, Mengmeng Lu, Qun Dai

**Affiliations:** 1 The Affiliated Stomatological Hospital of Nanchang University, The Key Laboratory of Oral Biomedicine, 49 Fuzhou Road, Nanchang, 330000 China; 2 Department of Oral and Maxillofacial Surgery, Shanghai Stomatological Hospital, Fudan University, 1258 Fuxin Zhong Road, Shanghai, 200031 China

**Keywords:** Gene set variation analysis, Tongue cancer, Survival analysis, Lymph node metastasis

## Abstract

**Background:**

Lymph node metastasis is one of the most important factors affecting the prognosis of tongue cancer, and the molecular mechanism regulating lymph node metastasis of tongue cancer is poorly known.

**Methods:**

The gene expression dataset GSE2280 and The Cancer Genome Atlas (TCGA) tongue cancer dataset were downloaded. R software was used to identify the differentially expressed hallmark gene sets and individual genes between metastatic lymph node tissues and primary tongue cancer tissues, and the Kaplan–Meier method was used to evaluate the association with overall survival. The screening and validation of functional genes was performed using western blot, q-PCR, CCK-8, migration and invasion assays, and lymphangiogenesis was examined by using a tube formation assay.

**Results:**

Thirteen common hallmark gene sets were found based on Gene Set Variation Analysis (GSVA) and then subjected to differential gene expression analysis, by which 76 deregulated genes were found. Gene coexpression network analysis and survival analysis further confirmed that IER3 was the key gene associated with the prognosis and lymph node metastasis of tongue cancer patients. Knockdown of IER3 with siRNA inhibited the proliferation, colony formation, migration and invasion of Tca-8113 cells in vitro and it also inhibited the secretion and expression of VEGF-C in these cells. The culture supernatant of Tca-8113 cells could promote lymphangiogenesis and migration of lymphatic endothelial cells, and knockdown of IER3 in Tca-8113 cells suppressed these processes.

**Conclusion:**

Our study demonstrated that IER3 plays important roles in lymphangiogenesis regulation and prognosis in tongue cancer and might be a potential therapeutic target.

## Background

Tongue cancer is one of the most common cancers in the oral cavity, most of which are squamous cell carcinomas [1, 2]. Lymph node metastasis tends to occur during the early stage of tongue cancer, and it is one of the important factors leading to poor prognosis [3]. Lymph node metastasis during tumor progression involves the deregulation of multiple gene and signaling pathways. In this process cancer cells separate from the primary tumor site and penetrate the basement membrane by degrading the extracellular matrix and then permeate into the lymphatic vessels. Many signaling pathways are associated with this process, such as PI3K-AKT, hedgehog, and NF-κB signaling pathways [4–6], but the molecular process in tongue cancer remains unknown [7]. Therefore, finding key genes and pathways involved in lymph node metastasis and inhibiting the progression of tongue cancer at the molecular level is expected to improve the prognosis of patients with tongue cancer.

In recent years, with the development of bioinformatics, genomic sequencing, gene chip, and data mining have played an important role in personalized medicine [8, 9]. Using bioinformatic techniques to mine and analyze microarrays and sequencing datasets can provide a new direction for studies on tumor development and progression. GEO (Gene Expression Omnibus) is a public database that contains microarray, sequencing and high-throughput functional genomic datasets [10]. TCGA (The Cancer Genome Atlas) provides genomic sequencing datasets and clinical data for 33 types of cancer [11]. Both GEO and TCGA have made lasting contributions to understanding the mechanisms of cancer development and progression and have identified novel targets for cancer therapy [12].

In this study, we identified that IER3 was an important gene associated with lymph node metastasis and prognosis in tongue cancer patients by analyzing the GSE2280 RNA microarray dataset and the TCGA tongue cancer RNA-seq dataset. We then investigated the roles of IER3 in tongue cancer cells by performing proliferation, migration and invasion assays, and we also demonstrated that IER3 could promote lymphangiogenesis in vitro.

## Methods and materials

### Data source

The dataset of primary tongue cancer tissues and metastatic lymph node tissues was downloaded from the GEO database. The accession number was GSE2280 [13]. The microarray data of GSE2280 was based on GPL96 ([HG-U133A] Affymetrix Human Genome U133A Array). The preprocessed level 3 RNA-seq data and clinical information of 148 tongue cancer patients with detailed follow-up information were downloaded from the TCGA data portal.

### Data analysis

The t scores of hallmark gene sets larger than 1 were obtained by using the “GSVA” package (Gene Set Variation Analysis) in R language [14], and hallmark gene sets are coherently expressed signatures derived by aggregating many MSigDB gene sets to represent well-defined biological states or processes [15]. Differentially expressed genes with log FC > 1 and P value < 0.05 were obtained by using the “limma” package [16]. The “pheatmap” package in R software was used to visualize differentially expressed genes. The Benjamini and Hochberg method was used to calculate the P value. Cytoscape software was used to establish a gene coexpression network and determine hub genes. The “survival” and “survminer” packages were used to perform survival analysis and create survival curves.

### Cell culture

Human oral tongue squamous carcinoma cell line Tca-8113 and human lymphatic endothelial cells (LECs) were purchased from the Tumor Cell Bank of the Chinese Academy of Medical Science (Beijing, China). Tongue cancer cell lines were cultured in DMEM (Gibco, USA) with 10% fetal bovine serum (FBS, HyClone, USA) and 1% penicillin/streptomycin at 37 °C with 5% CO_2_. LECs were maintained in RPMI-1640 medium (Gibco, USA) with 10% fetal bovine serum and 1% penicillin/streptomycin.

### Migration and invasion assays

Cell migration and invasion assays were performed using a 24-well transwell chamber with an 8 μm pore size polycarbonate membrane (Corning, Corning, NY, USA). For the invasion assay, the membranes of 24-well transwell chambers were pretreated with Matrigel (BD Biosciences, USA) in 200 μl serum-free medium. Briefly, 2 × 10^5^ control or transfected cells were seeded on the chamber, and 500 μl culture medium with 10% FBS was added to the lower chamber. After incubating for 24 h, the cells were fixed with 5% paraformaldehyde for 20 min. After removing the cells on the upper surface of the chamber, the cells on the lower surface of the chamber were stained with 0.1% crystal violet. The number of cells was counted in five random fields, and photos were taken under a microscope (Olympus).

### Cell proliferation assay

The cell proliferation assay was performed using the Cell Counting Kit-8 (CCK-8, Dojindo, Japan). A total of 4 × 10^3^ control or transfected cells were seeded into 96-well plates. After incubating for 24 h, 48 h and 72 h, 10 μl CCK-8 solution was added to each well and then incubated for another 4 h at 37 °C. The 450 nm OD value was measured using a microplate spectrophotometer (BioTek, USA).

### Colony formation assay

A total of 5 × 10^2^ control or transfected cells were seeded in 6-well plates. The culture medium was refreshed every 4 days. After incubating for 14 days, colonies were fixed with 5% paraformaldehyde for 20 min and then stained with 0.1% crystal violet solution for 15 min. The number of colonies was counted, and the cells were photographed with a camera.

### Enzyme-linked immunosorbent assay (ELISA)

ELISA was used to determine the effects of si-IER3 on the secretion of VEGF-C in tongue cancer cells.

The cell culture medium of Tca-8113 and si-IER3-treated Tca-8113 cells was collected, and a VEGF-C ELISA kit (eBioscience, Thermo Fisher, USA) was used to examine VEGF-C expression according to the manufacturer’s instructions.

### RNA interference

IER3 siRNA was purchased from RiboBio (Guangzhou). Cells were transfected with either 5 μM si-IER3-1 or si-IER3-2 using Lipofectamine 3000 (Invitrogen, USA) according to the manufacturer’s protocols, control group in this study was only transfected with Lipofectamine 3000 without Si-RNA. The expression of IER3 was evaluated by Western blot and q-PCR. The si-IER3-1 sequence was 5′-AGCAGCAGAAAGAGAAGAAGCCtt-3′. The si-IER3-2 sequence was 5′-GGAAGGAGAGCGUCGUUAA-3′.

### RNA isolation and quantitative real-time polymerase chain reaction

Total RNA was extracted from cells using TRIzol (Takara, Japan) according to the manufacturer’s instructions. A total of 500 ng of total RNA was used for cDNA synthesis by using a reverse transcription kit (Takara, Japan). qRT-PCR was performed using a 7500 HT Fast Real-Time PCR System (Applied Biosystems, USA). mRNA expression was normalized with GAPDH, and data are presented as an expression fold change using the 2−ΔΔCt method. Primers were purchased from Sangon Biotech (China). The IER3 primer sequences were forward 5′-TCCTGTTTTGTCTCCCCTTACG-3′ and reverse 5′-TCAGGATCTGGCAGAAGACGAT-3′. The VEGF-C primer sequences were forward 5′-CAGTTACGGTCTGTGTCCAGTGTAG-3′ and reverse 5′-GGACACACATGGAGGTTTAAAGAAG-3′. The VEGF-D primer sequences were forward 5′-TGGGTCATCTTCTCGCGGTT-3′ and reverse 5′-GATGATGATATCGCCGCGCT-3′..

### Tube formation assay

The tube formation assay was performed using μ-Plate Angiogenesis 96 Well according to the manufacturer’s instructions and a previous study. Briefly, the Matrigel (BD Biosciences, USA) coat formed a solid structure on the bottom of a 96-well plate. Control or transfected LECs were suspended in culture media and seeded onto Matrigel-pretreated 96-well plates. After incubation, LECs were observed for tube formation under a light-field microscope. The tubes were imaged, and the number of tubes was counted.

### Western blot analysis

Total protein was extracted from cells with lysis buffer (KeyGEN BioTECH, China). Total protein samples were separated by SDS-PAGE (10–15%) and transferred to a PVDF membrane. After blocking with 5% defatted milk for 1 h, the membrane was incubated with primary antibodies overnight at 4 °C and then further incubated with HRP-conjugated secondary antibody for 1 h at room temperature. Protein bands were detected using chemiluminescence (KeyGEN BioTECH, China).

### Statistical analysis

All data are presented as the mean ± standard deviation (SD). Student’s t-test was used to calculate the P-value when comparing two groups. A one-way analysis of variance (ANOVA) was used when comparing more than two groups. SPSS 18.0 was used to analyze all data. In all cases, two-sided P-values < 0.05 were considered statistically significant.

## Results

### Analysis of hallmark gene sets involved in lymph node metastasis

**Fig. 1:**
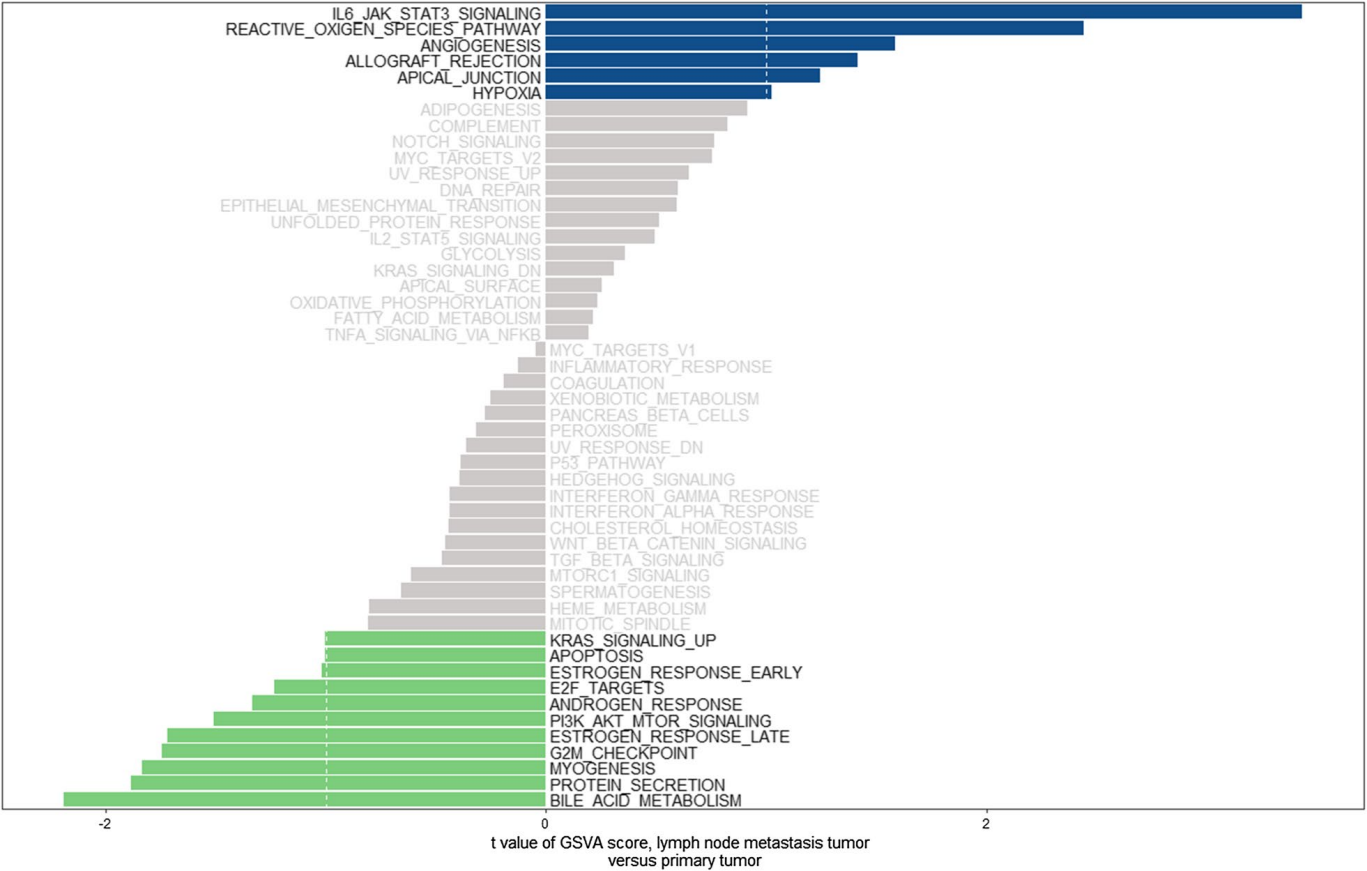
The changed Hallmark gene sets of metastatic lymph node versus primary tumor. A t value > 1 or < − 1 represents statistically significant changes

Hallmark gene sets consist of fifty sets that represent over four thousands original overlapping gene sets from v4.0 MSigDB collections C1 to C6, and provide accurate results when used with enrichment analysis methods in cancer [15]. We only found one dataset (GSE2280) of tongue cancer that compared the gene expression of metastatic lymph node with primary cancer tissues from GEO datasets. Then, we analyzed the changes in hallmark gene sets in metastatic lymph nodes through GSVA. As shown in Fig. 1, we found that six hallmark gene sets were upregulated (t score > 1), eleven hallmark gene sets were downregulated (t score < − 1), and in total the changed hallmark gene sets contained 1839 genes.

### Screening of hub genes involved in lymph node metastasis

**Fig. 2:**
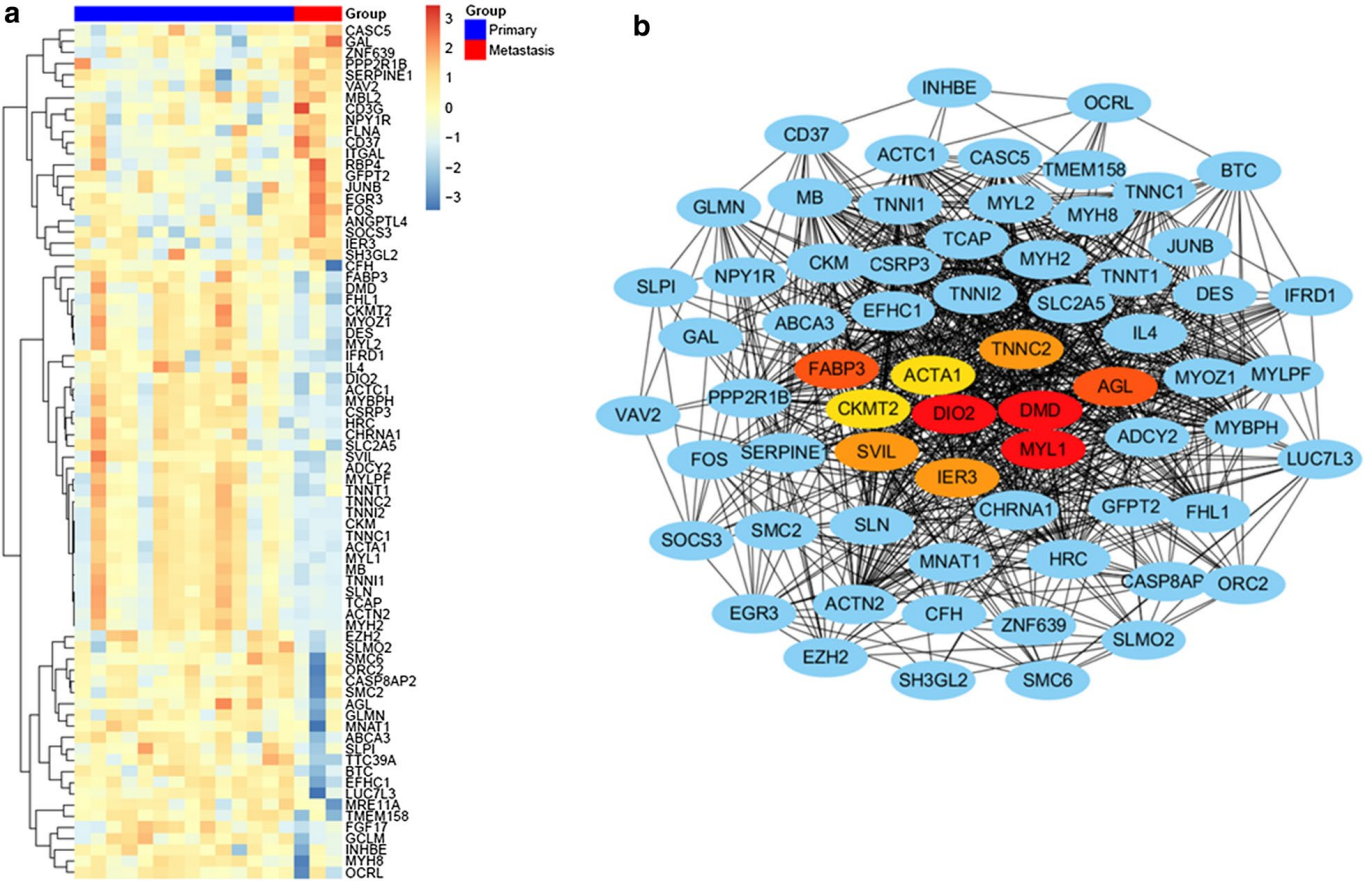
Screening of hub genes involved with lymph node metastasis. **a** Heatmap of the deregulated genes in metastatic lymph node versus primary tumor. **b** Gene coexpression network of the 76 differentially expressed genes. The red, yellow and orange ovals represent ten candidate hub genes in the network

To further investigate the hub genes from these hallmark gene sets that were involved in lymph node metastasis, we analyzed the change in expression of the 1839 genes in metastatic lymph nodes and primary tumors (Additional file 1: Table S1). As shown in Fig. 2a, in a total of 1839 genes, 76 genes were deregulated, of which 21 genes were upregulated and 55 genes were downregulated. Most of these deregulated genes were enriched in MYOGENESIS, KRAS_SIGNALING_UP, HYPOXIA, G2M_CHECKPOINT, and E2F_TARGETS (Table 1). Then, a molecular network of the deregulated genes was established, and the top 10 deregulation hub genes were identified by Cytoscape (Fig. 2b), of which only the expression of IER3 was upregulated (LogFC = 1.38, P = 0.015), while the other 9 genes were downregulated (Table 2).

**Table 1:** A total of 76 deregulated genes were identified in 1839 primary tumor versus metastatic lymph node genes

Gene symbol	logFC	P value	Hallmark
MBL2	1.836355	0.016409	ALLOGRAFT_REJECTION
ITGAL	1.09615	0.029208	ALLOGRAFT_REJECTION
CD3G	1.084988	0.023672	ALLOGRAFT_REJECTION
FLNA	1.003019	0.037726	ALLOGRAFT_REJECTION
GLMN	− 1.11045	0.044154	ALLOGRAFT_REJECTION
VAV2	1.222483	0.037575	ANGIOGENESIS, APICAL_JUNCTION
ACTA1	− 4.81243	0.007734	APICAL_JUNCTION
EFHC1	− 1.33106	0.001242	BILE_ACID_METABOLISM
DIO2	− 1.48714	0.003577	BILE_ACID_METABOLISM
SMC6	− 1.01378	0.036294	E2F_TARGETS
ORC2	− 1.02171	0.039099	E2F_TARGETS
LUC7L3	− 1.21609	0.002313	E2F_TARGETS
MRE11A	− 1.23003	0.045764	E2F_TARGETS
ABCA3	− 1.4387	0.009719	ESTROGEN_RESPONSE_EARLY, ESTROGEN_RESPONSE_LATE
TTC39A	− 1.90927	0.002779	ESTROGEN_RESPONSE_EARLY
NPY1R	2.020464	0.03779	ESTROGEN_RESPONSE_LATE, ESTROGEN_RESPONSE_EARLY
GAL	1.468347	0.023249	ESTROGEN_RESPONSE_LATE
EGR3	1.177485	0.036595	ESTROGEN_RESPONSE_LATE, ESTROGEN_RESPONSE_EARLY, KRAS_SIGNALING_UP
CASC5	1.897606	0.036429	G2M_CHECKPOINT
CASP8AP2	− 1.09086	0.016979	G2M_CHECKPOINT
EZH2	− 1.18721	0.018306	G2M_CHECKPOINT, E2F_TARGETS
MNAT1	− 1.27108	0.027402	G2M_CHECKPOINT
SMC2	− 1.33982	0.04864	G2M_CHECKPOINT
SERPINE1	1.733088	0.021088	HYPOXIA
FOS	1.543577	0.005153	HYPOXIA
IER3	1.387516	0.015602	HYPOXIA, APOPTOSIS
ANGPTL4	1.261686	0.018984	HYPOXIA, KRAS_SIGNALING_UP, ADIPOGENESIS
SLC2A5	− 2.14747	0.047968	HYPOXIA
SOCS3	1.72843	0.019538	IL6_JAK_STAT3_SIGNALING
INHBE	− 1.17054	0.031406	IL6_JAK_STAT4_SIGNALING
ZNF639	2.511151	0.001222	KRAS_SIGNALING_UP
RBP4	2.229625	0.033045	KRAS_SIGNALING_UP
CD37	1.547497	0.019287	KRAS_SIGNALING_UP
GFPT2	1.031506	0.042835	KRAS_SIGNALING_UP
SLMO2	− 1.00092	0.02985	KRAS_SIGNALING_UP
CFH	− 1.6692	0.040962	KRAS_SIGNALING_UP
SLPI	− 1.67431	0.014661	KRAS_SIGNALING_UP
BTC	− 2.14105	0.00012	KRAS_SIGNALING_UP
TMEM158	− 2.44114	0.000586	KRAS_SIGNALING_UP
SVIL	− 1.15372	0.004237	MYOGENESIS
IFRD1	− 1.29777	0.000871	MYOGENESIS
AGL	− 1.34563	0.006822	MYOGENESIS
FABP3	− 1.39162	0.018123	MYOGENESIS
CKMT2	− 1.43326	0.0434	MYOGENESIS
MYH8	− 1.48548	0.015832	MYOGENESIS
DMD	− 1.53731	0.006311	MYOGENESIS, G2M_CHECKPOINT
MYOZ1	− 1.74048	0.041979	MYOGENESIS
CHRNA1	− 1.8081	0.017455	MYOGENESIS
HRC	− 2.02106	0.038865	MYOGENESIS
FHL1	− 2.03688	0.035865	MYOGENESIS
SLN	− 2.11469	0.041452	MYOGENESIS
ACTC1	− 2.90968	0.011697	MYOGENESIS
DES	− 3.03388	0.031095	MYOGENESIS
MYBPH	− 3.21711	0.003079	MYOGENESIS
TNNT1	− 3.34517	0.024009	MYOGENESIS
MYH2	− 3.3503	0.042848	MYOGENESIS
MB	− 3.35038	0.010556	MYOGENESIS
TCAP	− 3.38871	0.029364	MYOGENESIS
MYLPF	− 3.50266	0.020669	MYOGENESIS
TNNC2	− 3.52148	0.020435	MYOGENESIS
MYL2	− 3.59938	0.011658	MYOGENESIS
ACTN2	− 4.31463	0.01354	MYOGENESIS
TNNI2	− 4.42965	0.011318	MYOGENESIS
MYL1	− 4.54843	0.006995	MYOGENESIS
CKM	− 4.71858	0.011289	MYOGENESIS
TNNI1	− 4.77636	0.0062	MYOGENESIS
TNNC1	− 5.64825	0.007417	MYOGENESIS
CSRP3	− 5.77325	0.012999	MYOGENESIS
PPP2R1B	1.171797	0.042423	PI3K_AKT_MTOR_SIGNALING
FGF17	− 1.34021	0.032683	PI3K_AKT_MTOR_SIGNALING
IL4	− 1.52042	0.040415	PI5K_AKT_MTOR_SIGNALING, IL6_JAK_STAT3_SIGNALING
ADCY2	− 1.91434	0.025949	PI6K_AKT_MTOR_SIGNALING
SH3GL2	1.508413	0.042024	PROTEIN_SECRETION
OCRL	− 1.09672	0.008807	PROTEIN_SECRETION
JUNB	1.047607	0.019591	REACTIVE_OXIGEN_SPECIES_PATHWAY
GCLM	− 1.71176	0.003821	REACTIVE_OXIGEN_SPECIES_PATHWAY, BILE_ACID_METABOLISM

**Table 2:** Top 10 hubgenes in 76 deregulated genes

Gene	logFC	AveExpr	t	P value
ACTA1	− 4.81243	8.399569	− 2.99088	0.007734
AGL	− 1.34563	7.828326	− 3.04814	0.006822
CKMT2	− 1.43326	6.654878	− 2.16996	0.0434
DIO2	− 1.48714	7.679014	− 3.34018	0.003577
DMD	− 1.53731	6.485171	− 3.08362	0.006311
FABP3	− 1.39162	5.339507	− 2.59505	0.018123
IER3	1.387516	10.10791	2.665727	0.015602
MYL1	− 4.54843	8.410023	− 3.03672	0.006995
SVIL	− 1.15372	9.014868	− 3.26402	0.004237
TNNC2	− 3.52148	6.850598	− 2.53798	0.020435

### Subsequent screening and validation of the hub genes using TCGA data

**Fig. 3:**
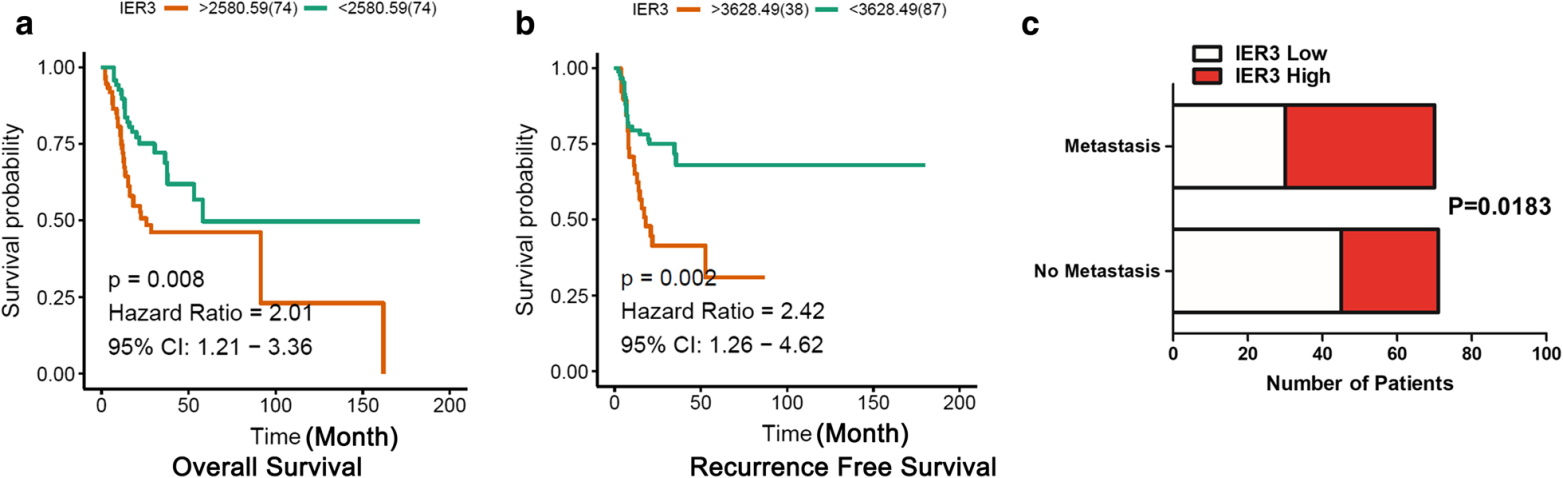
Expression of IER3 is correlated with overall survival and lymph node metastasis. **a** Overall survival of 148 tongue cancer patients (TCGA data) analyzed by the Kaplan–Meier method and log-rank test according to IER3 expression. **b** Recurrence-free survival of 148 tongue cancer patients (TCGA data) analyzed by the Kaplan–Meier method and log-rank test according to IER3 expression. **c** Correlations between the expression of IER3 and lymph node metastasis in tongue cancer patients (n = 141)

To investigate whether candidate genes are involved in lymph node metastasis and the prognosis of tongue cancer patients, we analyzed TCGA tongue cancer data for validation and for further screening of functional genes. Overall survival and lymph node metastasis data of TCGA tongue cancer patients were extracted from the TCGA-HNSC dataset. Based on these data, we found that patients with high expression of IER3 had poor prognosis (Fig. 3a), and those with high expression of IOD2 had better prognosis (Additional file 2: Figure S1); however, the other candidate genes had no significant association with overall survival (Additional file 2: Figure S1 and Table 3). More importantly, tongue cancer patients with lymph node metastasis showed significantly higher expression of IER3, and high expression of IER3 showed better recurrence-free survival (Fig. 3b, c).

**Table 3:** Overall survival analysis of the top 10 hub genes of TCGA tongue cancer patients

Gene	Hazard ratio	95% CI	P value
IER3	2.01	1.21–3.36	0.008
DIO2	0.59	0.35–0.98	0.045
CKMT2	1.66	0.93–2.97	0.055
SVIL	1.64	0.97–2.78	0.086
AGL	1.5	0.89–2.54	0.114
FABP3	1.56	0.92–2.64	0.082
ACTA1	1.44	0.84–2.49	0.164
DMD	0.75	0.45–1.25	0.264
MYL1	1.31	0.76–2.24	0.308
TNNC2	1.28	0.74–2.21	0.397

### Knockdown of IER3 inhibits the proliferation, migration and invasion of Tca-8113 cells

**Fig. 4:**
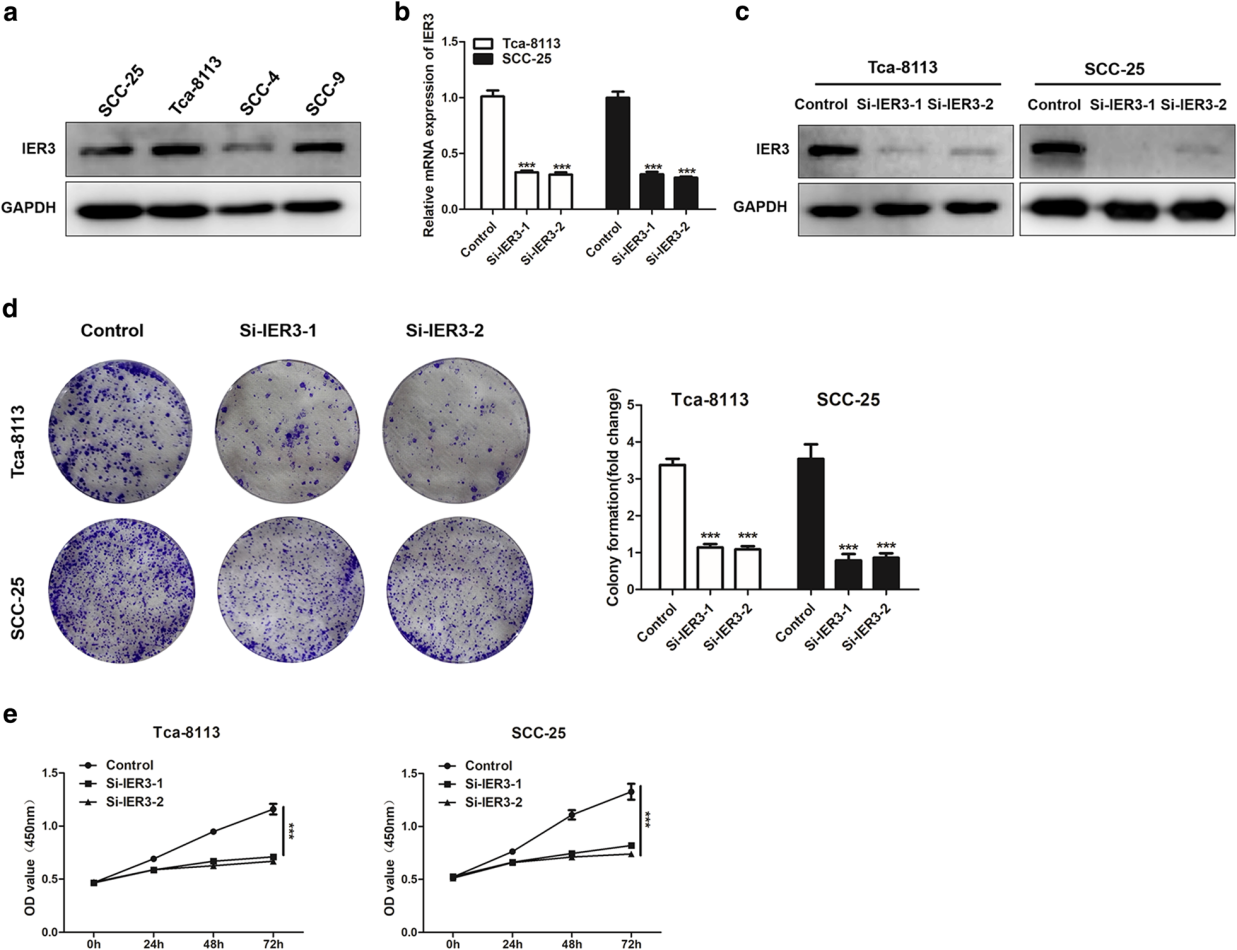
Knockdown of IER3 inhibits the proliferation of tongue cancer cells. **a** Protein expression of IER3 in tongue cancer cell lines (SCC-25 and Tca-8113) and oral squamous cell carcinoma cell lines (SCC-4 and SCC-9). **b** mRNA expression of IER3 in Tca-8113 and SCC-25 cells after transfection with siRNA. **c** Protein expression of IER3 in Tca-8113 and SCC-25 cells after transfection with siRNA. **d** Colony formation of Tca-8113 and SCC-25 cells with IER3 knockdown. Colony formation assay (left), quantification of colony formation assay (right). Data are presented as the mean ± SD (n = 3), *** indicates si-IER3-1 and si-IER3-2 versus control, P < 0.001. **e** OD value (450 nm) of Tca-8113 and SCC-25 cells after culture for 24 h, 48 h, and 72 h with IER3 knockdown

**Fig. 5:**
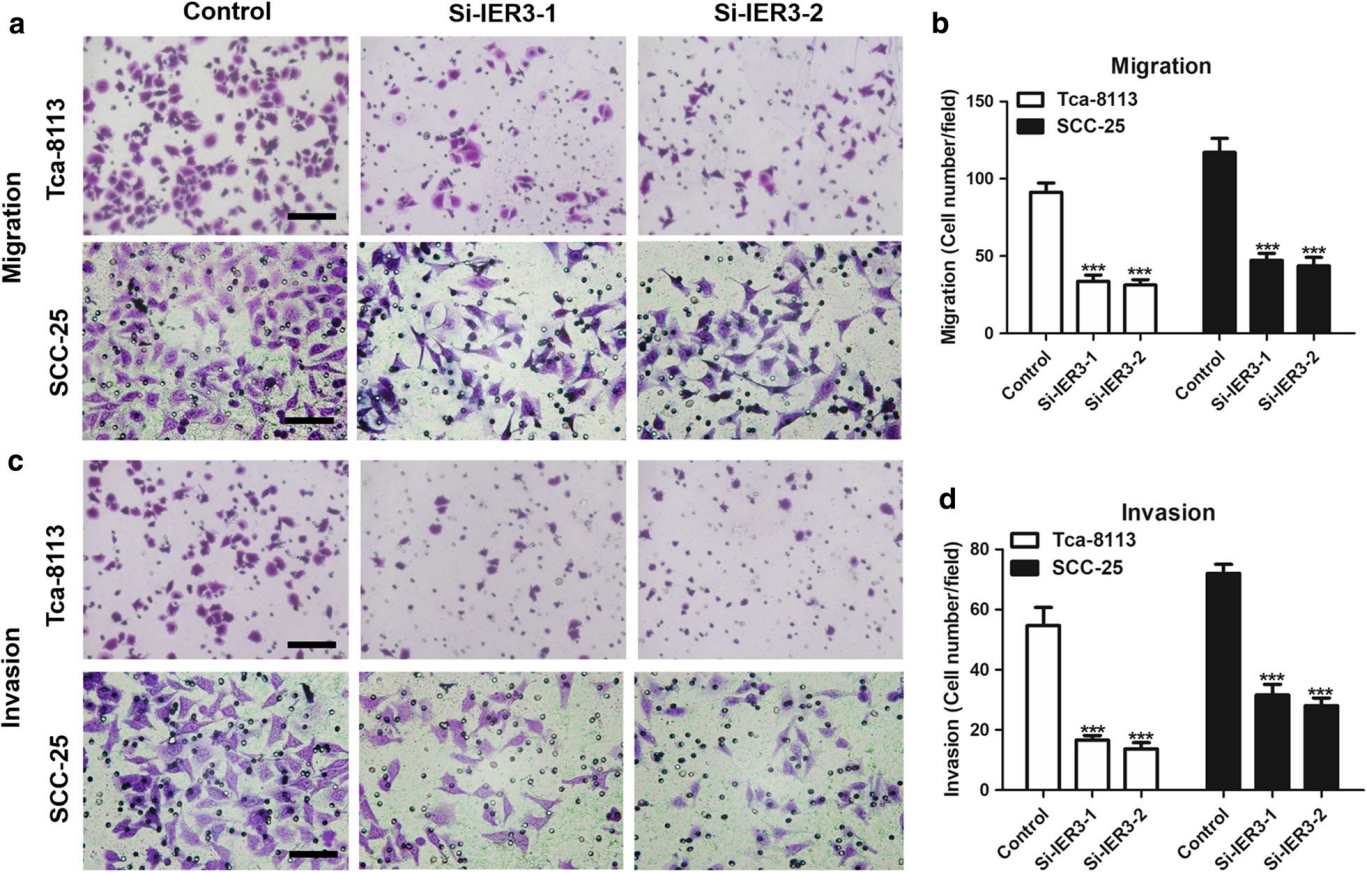
Knockdown of IER3 inhibits the migration and invasion of tongue cancer cells. **a**, **b** Migration of Tca-8113 and SCC-25 cells with IER3 knockdown (**a**), and quantification of the migration assay (**b**). **c**, **d** Invasion of Tca-8113 and SCC-25 cells with IER3 knockdown (**c**), and quantification of the invasion assay (**d**). Data are presented as the mean ± SD (n = 3), *** indicates si-IER3-1 and si-IER3-2 versus control, P < 0.001. Black bar = 100 μm

As shown above, we found that IER3 might be a key gene that promotes the progression and lymph node metastasis of tongue cancer. We examined the protein expression of IER3 in 2 tongue cancer cell lines and 2 oral squamous cell carcinoma cell lines, and the results showed that all 4 oral cancer cell lines expressed IER3 (Fig. 4a). We knocked down the expression of IER3 in Tca-8113 cells by transfecting specific siRNA, and the validation results are shown in Fig. 4b, c. The results of proliferation and colony formation assays suggested that knocking down IER3 could significantly suppress cell proliferation in Tca-8113 and SCC-25 cells (Fig. 4d, e). Furthermore, Fig. 5 shows that knockdown of IER3 inhibited the migration and invasion of Tca-8113 and SCC-25 cells (Fig. 5a–d).

### Knockdown of IER3 in Tca-8113 cells inhibits lymphangiogenesis and migration of LECs

**Fig. 6:**
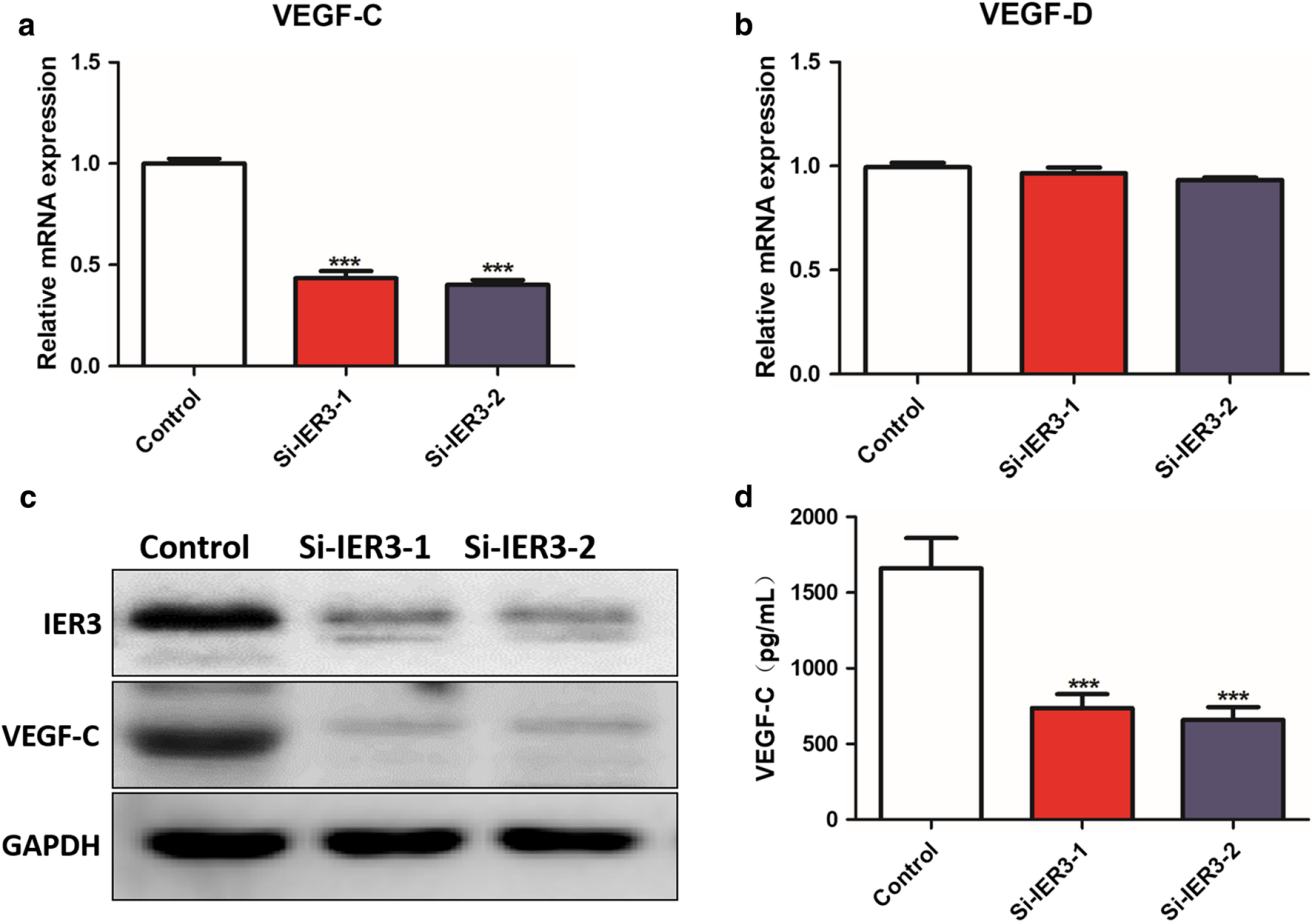
Knockdown of IER3 inhibits the expression of VEGF-C in tongue cancer cells. **a**, **b** The mRNA expression of VEGF-C (**a**) and VEGF-D (**b**) in Tca-8113 cells after IER3 knockdown. **c** Immunoblot analysis of VEGF-C and IER3 in Tca-8113 cells with IER3 knockdown. **d** ELISA analysis of VEGF-C in Tca-8113 cells with IER3 knockdown. Data are presented as the mean ± SD (n = 3), *** indicates si-IER3-1 and si-IER3-2 versus control, P < 0.001

**Fig. 7:**
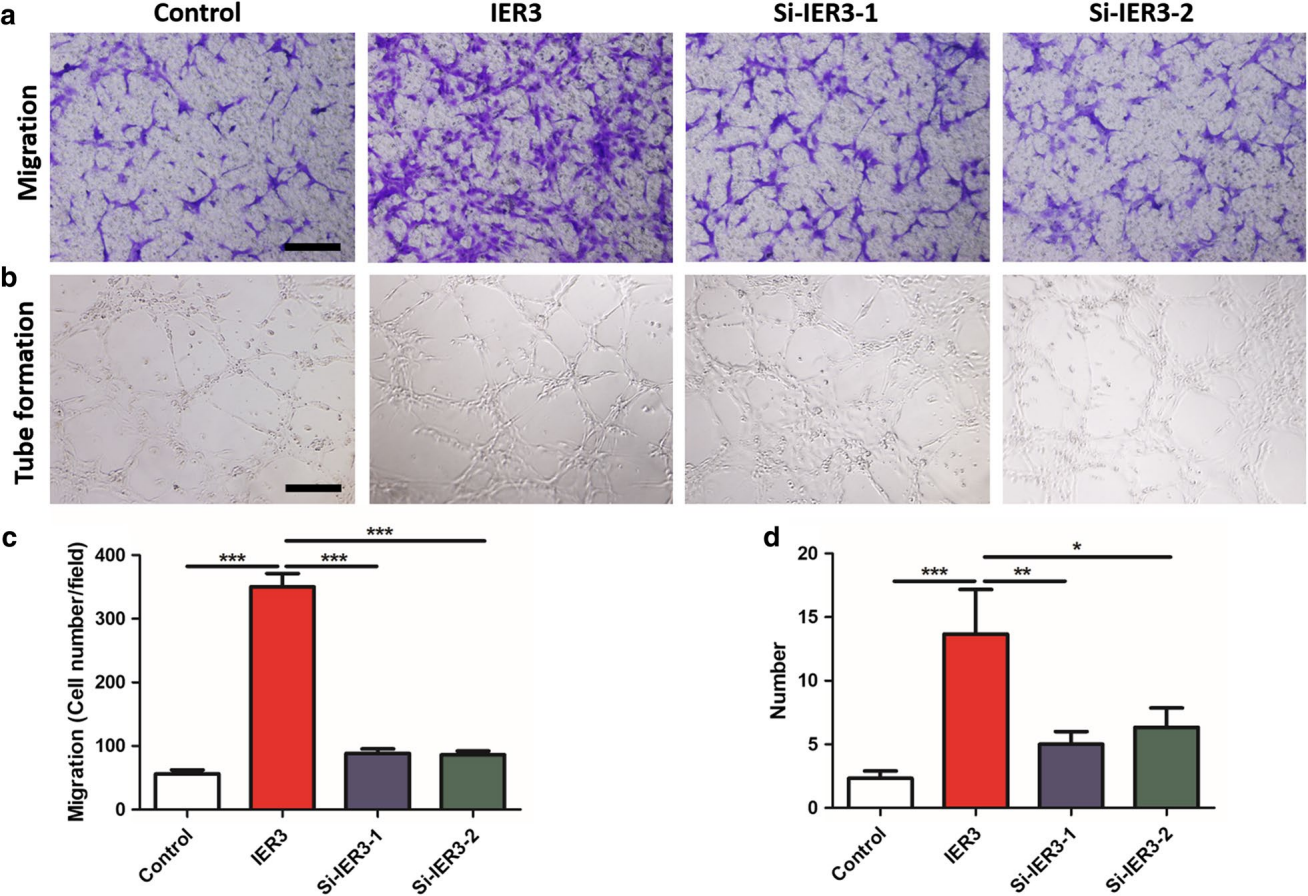
Knockdown of IER3 in tongue cancer cells inhibits the migration and tube formation of LECs. **a**, **c** Migration of LECs with the culture supernatant of IER3 knockdown Tca-8113 cells (**a**), and quantification of the migration assay (**c**). **b**, **d** Tube formation of LECs with the culture supernatant of IER3-knockdown Tca-8113 cells (**b**), and quantification of the tube formation assay (**d**). Data are presented as the mean ± SD (n = 3), * indicates P < 0. 05, ** indicates P < 0.01, and *** indicates P < 0.001. Black bar = 100 µm

VEGF-C and VEGF-D have been identified as cytokines that promote lymphangiogenesis and lymph node metastasis in cancer. We found that knockdown of IER3 decreased the mRNA expression of VEGF-C but not VEGF-D in Tca-8113 cells (Fig. 6a, b), and Western blot results showed that the protein expression of VEGF-C also decreased (Fig. 6c). In addition, the results of the ELISA assay showed that the secretion of VEGF-C in Tca-8113 cells was decreased when IER3 was knocked down (Fig. 6d). We performed migration and tube formation assays in LECs using the culture supernatants of Tca-8113 and si-IER3-transfected Tca-8113 cells. The results showed that the number of migrated cells in the Tca-8113 group was significantly higher than that in the control and si-IER3 groups (Fig. 7a, c), and the tube number in the Tca-8113 group was also higher than that in the control and si-IER3 groups (Fig. 7b, d).

## Discussion

Tongue cancer is one of the most common cancers in oral squamous cell carcinoma, and the development of oral cancer is a multi-step process involving a variety of molecular and genomic deregulation steps. Lymph node metastasis is an important factor affecting the prognosis of tongue cancer, and the effective molecular targets remain unknown. In this study, we identified the hallmark gene sets and hub genes involved in the lymph node metastasis of tongue cancer. Moreover, based on TCGA data, we found that IER3 was the key gene that might promote lymph node metastasis and a potential marker of prognosis for tongue cancer patients. We validated the function of IER3 in tongue cancer cells, and the results showed that knocking down IER3 significantly decreased the proliferation, migration and invasion of Tca-8113 cells and decreased the secretion of VEGF-C, which reduced the tube formation and migration of LECs.

GSVA is a method that builds on GSEA and is better than GSEA for characterizing pathways from data obtained from both microarray and RNA-seq assays. GSVA is widely used in bioinformatic analysis of different tumors for investigating the molecular mechanism of cancer development and progression [17, 18]. Based on GSVA, we identified 17 hallmark gene sets involved in lymph node metastasis, of which the upregulated hallmark gene sets IL6_JAK_STAT3_SIGNALING, REACTIVE_OXIGEN_SPECIES_PATHWAY,ANGIOGENESIS, HYPOXIA and APICAL_JUNCTION were reportedly associated with lymph node metastasis in cancer [19–22]. However, the downregulated hallmark gene sets were also related to lymph node metastasis, such as KRAS_SIGNALING and PI3 K_AKT_MTOR_SIGNALING [23, 24], which indicated that the mechanism of lymph node metastasis is complicated and involves various activated and inactivated signaling pathways. In our study, each deregulated hallmark gene set contained many genes, not all of which were deregulated. Therefore, we analyzed the expression of all 1839 genes in the identified hallmark gene sets and found that 76 genes were deregulated and were mostly enriched in MYOGENESIS, ANGIOGENESIS, HYPOXIA, KRAS_SIGNALING_UP, and G2M_CHECKPOINT, of which MYOGENESIS, G2M_CHECKPOINT, and KRAS_SIGNALING_UP were downregulated in metastatic lymph nodes, and ANGIOGENESIS and HYPOXIA were upregulated. It has been reported that HYPOXIA and ANGIOGENESIS can promote lymph node metastasis in cancer [25, 26]. MYOGENESIS is associated with the formation of muscle tissue during embryonic development [27], and we speculate that its upregulation in metastatic lymph nodes might be associated with the differences in cells in primary and metastatic tumor tissues, such as muscles and fibroblasts, because tissue heterogeneity affects the accuracy of microarray and sequencing data [28]. The downregulation of G2M_CHECKPOINT and KRAS_SIGNALING_UP might contribute to the low proliferation ability of metastatic cancer cells [29]. Finally, we found that IER3 might be the key gene that could promote lymph node metastasis, and the results of validation using TCGA data could demonstrate it.

IER3, also known as IEX-1, p22/PRG1, Dif-2, or gly96, belongs to the early response gene family, which is involved in cell differentiation and cell proliferation [30]. IER3 is highly expressed in a variety of tumors and is associated with poor prognosis, such as bladder cancer, pancreatic cancer, breast cancer, melanoma, etc. [30, 31]. However, no studies have reported its relationship with tongue cancer. Our study found that high expression of IER3 was associated with poor prognosis and lymph node metastasis in patients with tongue cancer, suggesting that IER3 might have a cancer-promoting effect in tongue cancer.

We found that IER3 could promote the proliferation, invasion and migration of tongue cancer cells in vitro. Previous studies have reported that IER3 is involved in the activation of the PI3 K/AKT and MAPK/ERK signaling pathways [32], which promotes tumor progression, also suggesting that IER3 plays a role in promoting tongue cancer, but further in vivo evidence is needed. More importantly, we found that knocking down IER3 reduced the secretion of VEGF-C from tongue cancer cells, which could promote the formation of lymphatic vessels and lymph node metastasis [33, 34]. Our studies demonstrated that tongue cancer cell supernatant increased the tube formation of LECs, while the supernatant of IER3-downregulated tongue cancer cells could not, which indicated that IER3 could promote lymphangiogenesis by promoting the secretion of VEGF-C, and lymph node metastasis in tongue cancer was associated with this process. The activation of the PI3 K/AKT and JAK2/STAT3 signaling pathways could promote the secretion of VEGF-C in cancer cells [35, 36], suggesting that IER3 might induce the generation of VEGF-C in tongue cancer cells, but the specific molecular mechanism needs to be identified in further studies.

## Conclusions

Our study identified the changed hallmark gene sets between metastatic lymph node and primary tongue cancer that are associated with lymph node metastasis in tongue cancer. More importantly, we found that the hub gene IER3 might predict prognosis tongue cancer, and our in vitro experiments demonstrated that IER3 might promote the progression and lymph node metastasis in tongue cancer, which might be a potential therapeutic target.

## Supplementary information

**Additional file 1: Table S1.** New management strategies of oral tongue cancer in Bangladesh.

**Additional file 2: Figure S1.** Overall survival analysis of 9 hub genes of TCGA tongue cancer patients.

## Data Availability

The datasets used and/or analyzed during the study are available from the corresponding author on reasonable request.

## Abbreviations

GSVA: gene set variation analysis
GSEA: gene set enrichment analysis
TCGA: The Cancer Genome Atlas
OSCC: oral squamous cell carcinoma
OS: overall survival
RFS: recurrence-free survival
HR: hazard ratio
