# Supplementary material for: Expression profile analysis identifies IER3 to predict overall survival and promote lymph node metastasis in tongue cancer
Source: Cancer Cell Int. 2019 Nov 21;19:307. doi: 10.1186/s12935-019-1028-2 (PMC6873470; doi:10.1186/s12935-019-1028-2)
Supplement: Supplementary file 1 — Additional file 1: Table S1. New management strategies of oral tongue cancer in Bangladesh. [file 12935_2019_1028_MOESM1_ESM.docx]

|  | logFC | AveExpr | t | P.Value |
| --- | --- | --- | --- | --- |
| BTC | -2.14105 | 3.353918 | -4.86098 | 0.00012 |
| TMEM158 | -2.44114 | 7.039909 | -4.1475 | 0.000586 |
| ARFGEF2 | -0.84342 | 8.168435 | -3.98841 | 0.000837 |
| IFRD1 | -1.29777 | 7.265869 | -3.97098 | 0.000871 |
| CD28 | 0.905611 | 5.650838 | 3.834844 | 0.001182 |
| ZNF639 | 2.511151 | 3.48534 | 3.819938 | 0.001222 |
| EFHC1 | -1.33106 | 5.673557 | -3.81292 | 0.001242 |
| LUC7L3 | -1.21609 | 8.942118 | -3.53552 | 0.002313 |
| TTC39A | -1.90927 | 4.701272 | -3.45348 | 0.002779 |
| MYBPH | -3.21711 | 5.600187 | -3.40746 | 0.003079 |
| DIO2 | -1.48714 | 7.679014 | -3.34018 | 0.003577 |
| EVI5 | -0.94411 | 6.10788 | -3.32377 | 0.00371 |
| GCLM | -1.71176 | 7.810002 | -3.31057 | 0.003821 |
| SVIL | -1.15372 | 9.014868 | -3.26402 | 0.004237 |
| FOS | 1.543577 | 9.599422 | 3.175558 | 0.005153 |
| TNNI1 | -4.77636 | 5.47834 | -3.09169 | 0.0062 |
| DMD | -1.53731 | 6.485171 | -3.08362 | 0.006311 |
| AGL | -1.34563 | 7.828326 | -3.04814 | 0.006822 |
| MYL1 | -4.54843 | 8.410023 | -3.03672 | 0.006995 |
| TNNC1 | -5.64825 | 6.1409 | -3.01001 | 0.007417 |
| ACTA1 | -4.81243 | 8.399569 | -2.99088 | 0.007734 |
| BTBD3 | -0.73955 | 7.30058 | -2.9683 | 0.008125 |
| ELTD1 | -0.82987 | 6.402464 | -2.96148 | 0.008247 |
| ABCA5 | -0.79428 | 6.286039 | -2.93792 | 0.008681 |
| OCRL | -1.09672 | 6.496419 | -2.9313 | 0.008807 |
| ABCA3 | -1.4387 | 4.353384 | -2.88596 | 0.009719 |
| MB | -3.35038 | 8.997423 | -2.84783 | 0.010556 |
| LMNA | 0.810048 | 9.391353 | 2.82267 | 0.011146 |
| CKM | -4.71858 | 7.081455 | -2.81676 | 0.011289 |
| TNNI2 | -4.42965 | 6.131408 | -2.81555 | 0.011318 |
| MYL2 | -3.59938 | 8.011886 | -2.80182 | 0.011658 |
| ACTC1 | -2.90968 | 9.228316 | -2.80027 | 0.011697 |
| CSRP3 | -5.77325 | 5.925775 | -2.75119 | 0.012999 |
| PLCB1 | -0.79928 | 6.801262 | -2.73935 | 0.013333 |
| ACTN2 | -4.31463 | 5.742404 | -2.73215 | 0.01354 |
| SLPI | -1.67431 | 10.23708 | -2.69492 | 0.014661 |
| EED | -0.88977 | 7.32789 | -2.67844 | 0.015185 |
| IER3 | 1.387516 | 10.10791 | 2.665727 | 0.015602 |
| MYH8 | -1.48548 | 5.421094 | -2.65884 | 0.015832 |
| MBL2 | 1.836355 | 2.129861 | 2.641987 | 0.016409 |
| UBE2S | 0.717874 | 7.749259 | 2.637747 | 0.016557 |
| SMC3 | -0.97759 | 7.532347 | -2.63565 | 0.016631 |
| CASP8AP2 | -1.09086 | 6.425969 | -2.62588 | 0.016979 |
| CHRNA1 | -1.8081 | 6.613088 | -2.61283 | 0.017455 |
| ROCK1 | -0.7152 | 8.654199 | -2.60092 | 0.0179 |
| FABP3 | -1.39162 | 5.339507 | -2.59505 | 0.018123 |
| EZH2 | -1.18721 | 6.377157 | -2.59027 | 0.018306 |
| STAM | -0.78148 | 7.611603 | -2.58942 | 0.018339 |
| PFKL | 0.649254 | 7.452281 | 2.574785 | 0.018914 |
| ANGPTL4 | 1.261686 | 5.894179 | 2.573031 | 0.018984 |
| CD37 | 1.547497 | 6.089705 | 2.565501 | 0.019287 |
| SOCS3 | 1.72843 | 5.35841 | 2.55936 | 0.019538 |
| JUNB | 1.047607 | 9.504597 | 2.558066 | 0.019591 |
| DEGS1 | -0.82105 | 8.380158 | -2.54293 | 0.020224 |
| TNNC2 | -3.52148 | 6.850598 | -2.53798 | 0.020435 |
| MYLPF | -3.50266 | 6.437274 | -2.53255 | 0.020669 |
| TNFRSF1A | 0.790141 | 8.96073 | 2.531453 | 0.020717 |
| SERPINE1 | 1.733088 | 8.997299 | 2.522969 | 0.021088 |
| ZNF277 | -0.94977 | 6.445158 | -2.51397 | 0.021489 |
| MAP4K2 | 0.63567 | 1.408031 | 2.507604 | 0.021777 |
| PDLIM7 | 0.980458 | 7.580304 | 2.479609 | 0.023087 |
| GAL | 1.468347 | 5.538361 | 2.476254 | 0.023249 |
| CD3G | 1.084988 | 3.424158 | 2.467591 | 0.023672 |
| TNNT1 | -3.34517 | 6.806452 | -2.46079 | 0.024009 |
| ADCY2 | -1.91434 | 5.596298 | -2.4233 | 0.025949 |
| RELA | 0.579455 | 8.930924 | 2.422054 | 0.026016 |
| MNAT1 | -1.27108 | 6.050742 | -2.3969 | 0.027402 |
| SLC1A1 | -0.81815 | 5.520006 | -2.37938 | 0.028408 |
| ITGAL | 1.09615 | 5.293819 | 2.365852 | 0.029208 |
| TCAP | -3.38871 | 5.793547 | -2.36325 | 0.029364 |
| SLMO2 | -1.00092 | 6.807486 | -2.35525 | 0.02985 |
| DHRS3 | 0.793205 | 7.704477 | 2.347911 | 0.030302 |
| FDFT1 | -0.76226 | 8.757802 | -2.34466 | 0.030504 |
| CUL5 | -0.69915 | 7.936924 | -2.34158 | 0.030697 |
| DES | -3.03388 | 7.830319 | -2.33526 | 0.031095 |
| ATF1 | -0.89104 | 6.753846 | -2.33319 | 0.031227 |
| INHBE | -1.17054 | 3.697936 | -2.33039 | 0.031406 |
| SCP2 | -0.52666 | 8.824066 | -2.31799 | 0.032211 |
| RPS9 | 0.692552 | 10.75746 | 2.311348 | 0.032649 |
| FGF17 | -1.34021 | 4.10108 | -2.31084 | 0.032683 |
| ZFP36 | 0.932115 | 9.810018 | 2.307674 | 0.032894 |
| RBP4 | 2.229625 | 2.606597 | 2.30543 | 0.033045 |
| VAMP4 | -0.60841 | 6.841805 | -2.30452 | 0.033106 |
| PAK1 | -0.93383 | 4.74508 | -2.2991 | 0.033473 |
| DEK | -0.90314 | 9.478667 | -2.29225 | 0.033942 |
| TBK1 | -0.58224 | 7.95685 | -2.28317 | 0.034573 |
| COPB1 | -0.56986 | 9.951311 | -2.28051 | 0.034759 |
| TSTA3 | 0.68975 | 8.113729 | 2.273435 | 0.035261 |
| LAPTM5 | 0.734552 | 10.2606 | 2.272545 | 0.035324 |
| FHL1 | -2.03688 | 10.18665 | -2.26504 | 0.035865 |
| FAIM3 | 0.918289 | 7.342569 | 2.264424 | 0.035909 |
| RPS6KA5 | -0.69695 | 6.835356 | -2.25932 | 0.036282 |
| SMC6 | -1.01378 | 7.178253 | -2.25915 | 0.036294 |
| CASC5 | 1.897606 | 2.19134 | 2.257305 | 0.036429 |
| EGR3 | 1.177485 | 6.565499 | 2.255051 | 0.036595 |
| PPP1CA | 0.706566 | 8.906436 | 2.249429 | 0.037013 |
| RAB5A | -0.50265 | 9.148464 | -2.24624 | 0.037252 |
| HK2 | -0.87541 | 8.189964 | -2.24218 | 0.037557 |
| VAV2 | 1.222483 | 5.516089 | 2.241954 | 0.037575 |
| FLNA | 1.003019 | 9.943659 | 2.239961 | 0.037726 |
| NPY1R | 2.020464 | 3.632869 | 2.239111 | 0.03779 |
| JUN | 0.736171 | 9.379204 | 2.234309 | 0.038157 |
| PNN | 0.655539 | 10.21468 | 2.228883 | 0.038576 |
| THBS3 | 0.894523 | 6.365507 | 2.225709 | 0.038823 |
| HRC | -2.02106 | 4.612534 | -2.22516 | 0.038865 |
| ORC2 | -1.02171 | 5.846122 | -2.22217 | 0.039099 |
| ALDH9A1 | -0.52705 | 8.526383 | -2.21683 | 0.03952 |
| FTL | 0.547064 | 12.65868 | 2.206348 | 0.04036 |
| IL4 | -1.52042 | 2.297948 | -2.20566 | 0.040415 |
| USP1 | -0.71921 | 8.554322 | -2.20276 | 0.040651 |
| LY86 | 0.816169 | 7.445605 | 2.199006 | 0.040957 |
| CFH | -1.6692 | 6.85753 | -2.19894 | 0.040962 |
| SLN | -2.11469 | 8.664015 | -2.19299 | 0.041452 |
| PFKM | -0.85908 | 8.861614 | -2.18942 | 0.041749 |
| MYOZ1 | -1.74048 | 7.025425 | -2.18667 | 0.041979 |
| SH3GL2 | 1.508413 | 3.196615 | 2.18613 | 0.042024 |
| PPP2R1B | 1.171797 | 9.465111 | 2.181397 | 0.042423 |
| GFPT2 | 1.031506 | 6.99587 | 2.176544 | 0.042835 |
| MYH2 | -3.3503 | 7.797682 | -2.17639 | 0.042848 |
| CKMT2 | -1.43326 | 6.654878 | -2.16996 | 0.0434 |
| PTPN11 | -0.65862 | 9.097738 | -2.16803 | 0.043568 |
| GLMN | -1.11045 | 5.809134 | -2.16131 | 0.044154 |
| TEAD4 | -0.83199 | 7.369216 | -2.15451 | 0.044754 |
| MRE11A | -1.23003 | 5.485563 | -2.14325 | 0.045764 |
| ABLIM1 | -0.80487 | 9.245346 | -2.13509 | 0.046509 |
| PRKAA2 | -0.92006 | 5.009845 | -2.13033 | 0.046949 |
| MEIS1 | -0.76802 | 6.290633 | -2.12748 | 0.047215 |
| ELANE | 0.838477 | 2.090507 | 2.126475 | 0.047308 |
| PDS5B | -0.46187 | 7.297964 | -2.12387 | 0.047552 |
| SLC2A5 | -2.14747 | 5.371399 | -2.11946 | 0.047968 |
| RHOB | 0.777798 | 9.641631 | 2.113503 | 0.048535 |
| SMC2 | -1.33982 | 6.373141 | -2.11241 | 0.04864 |
| COX6A2 | -1.15398 | 7.299486 | -2.09556 | 0.050279 |
| MYH1 | -3.47979 | 5.666981 | -2.0945 | 0.050384 |
| PKLR | 1.189516 | 3.217178 | 2.088749 | 0.050955 |
| CCND1 | 1.225274 | 7.674469 | 2.088395 | 0.050991 |
| TOM1L1 | -1.05401 | 6.726923 | -2.08403 | 0.051429 |
| CLDN9 | 0.633448 | 5.965108 | 2.07806 | 0.052034 |
| SLC2A8 | -1.25241 | 4.447316 | -2.07562 | 0.052283 |
| CACNA2D2 | -1.14343 | 4.371548 | -2.07257 | 0.052597 |
| MAP3K1 | 1.466133 | 4.41465 | 2.070026 | 0.052859 |
| RAC2 | 0.728392 | 8.859688 | 2.06895 | 0.05297 |
| OPTN | -0.51843 | 8.609926 | -2.06595 | 0.053281 |
| MYH3 | -2.73221 | 4.015688 | -2.06072 | 0.053828 |
| AP2M1 | 0.653886 | 9.664655 | 2.060696 | 0.053831 |
| HSD17B6 | -1.23498 | 5.41044 | -2.04469 | 0.055536 |
| CUL1 | -0.53682 | 7.714688 | -2.04111 | 0.055925 |
| UGDH | -0.61073 | 7.965527 | -2.03556 | 0.056531 |
| TGFBI | 1.215008 | 11.36243 | 2.031889 | 0.056935 |
| CRLF2 | 0.691031 | 3.338411 | 2.019425 | 0.058328 |
| MYL12B | -0.77128 | 11.07408 | -2.01738 | 0.058559 |
| MYL4 | -0.72382 | 7.388979 | -2.00679 | 0.05977 |
| SCNN1A | -1.60376 | 7.265366 | -2.00614 | 0.059846 |
| SLC25A1 | 0.470826 | 6.836896 | 1.995448 | 0.061093 |
| CHRNB1 | -0.67889 | 6.26509 | -1.99396 | 0.061268 |
| RBBP8 | -1.05139 | 8.457301 | -1.98924 | 0.061828 |
| DR1 | -0.67426 | 7.52307 | -1.98208 | 0.062685 |
| TUBB | 0.719162 | 11.34896 | 1.981313 | 0.062778 |
| GAA | -0.70898 | 6.645716 | -1.97035 | 0.064113 |
| BET1 | -0.42336 | 7.187255 | -1.96354 | 0.064955 |
| FHL2 | 0.732484 | 9.289566 | 1.961223 | 0.065244 |
| AP1G1 | -0.71004 | 6.528666 | -1.95126 | 0.066499 |
| BIRC3 | -0.9638 | 5.90091 | -1.95079 | 0.066558 |
| PRKCB | 0.716211 | 6.545397 | 1.949736 | 0.066692 |
| CRTAM | -1.21158 | 3.788188 | -1.94953 | 0.066719 |
| IRS2 | -0.97084 | 8.345969 | -1.94645 | 0.067112 |
| CLDN7 | -1.40352 | 6.724111 | -1.93583 | 0.068485 |
| ERCC2 | 1.040242 | 4.292175 | 1.933566 | 0.06878 |
| SRPX | 0.986089 | 8.14184 | 1.93153 | 0.069047 |
| MYH4 | -1.62477 | 2.534634 | -1.93136 | 0.06907 |
| POLQ | -0.85611 | 6.118613 | -1.92274 | 0.07021 |
| LPIN1 | -0.76206 | 6.994164 | -1.92261 | 0.070227 |
| CD36 | 1.174771 | 6.260673 | 1.917679 | 0.070888 |
| CARTPT | 0.999684 | 5.084395 | 1.917137 | 0.070961 |
| HDLBP | 0.472105 | 9.040081 | 1.914512 | 0.071315 |
| PDLIM3 | -1.55255 | 8.488986 | -1.9087 | 0.072105 |
| CBX1 | -0.54799 | 8.537909 | -1.90483 | 0.072634 |
| BTG1 | 0.612696 | 9.818755 | 1.902305 | 0.072982 |
| IL18 | -0.91911 | 6.400475 | -1.90105 | 0.073155 |
| CBR4 | -0.57554 | 6.494363 | -1.90099 | 0.073163 |
| FAM63A | -0.48551 | 7.50221 | -1.89776 | 0.073611 |
| PIK3R3 | -0.55202 | 6.776443 | -1.88484 | 0.075426 |
| DLC1 | 1.092111 | 7.165873 | 1.876593 | 0.076606 |
| ANXA10 | 1.05609 | 3.904279 | 1.875751 | 0.076728 |
| TNNT3 | -1.49003 | 7.309403 | -1.8684 | 0.077795 |
| MMP11 | -1.61452 | 8.699879 | -1.85761 | 0.079384 |
| BPGM | -0.82187 | 7.315811 | -1.85399 | 0.079924 |
| UBE2D1 | -0.49191 | 7.225961 | -1.85391 | 0.079935 |
| NEDD4 | -0.59378 | 6.948458 | -1.84903 | 0.080667 |
| IL17RA | 0.550438 | 7.315356 | 1.84889 | 0.080689 |
| HAS1 | 1.243427 | 4.482204 | 1.846707 | 0.081019 |
| TRIB1 | 0.710883 | 8.821871 | 1.844814 | 0.081305 |
| CD14 | 1.063038 | 8.501426 | 1.834932 | 0.082817 |
| P2RY2 | -1.55959 | 3.92952 | -1.83212 | 0.083252 |
| KCNH1 | 1.393664 | 3.231772 | 1.82747 | 0.083975 |
| RYR1 | -1.32744 | 7.247994 | -1.82527 | 0.08432 |
| KRT13 | -3.11092 | 7.945391 | -1.82484 | 0.084387 |
| PSIP1 | -0.65987 | 7.371326 | -1.82373 | 0.084562 |
| ATP2A1 | -1.59142 | 6.820758 | -1.82183 | 0.08486 |
| CCND3 | 0.474845 | 8.032941 | 1.818743 | 0.085348 |
| BHLHE40 | 0.632598 | 9.664997 | 1.814321 | 0.086051 |
| ENG | 0.752814 | 7.569602 | 1.813638 | 0.08616 |
| CDK8 | -0.56031 | 5.960258 | -1.81276 | 0.0863 |
| KIF20B | -0.64917 | 5.793015 | -1.81255 | 0.086333 |
| BBOX1 | -1.64601 | 4.820123 | -1.80582 | 0.087416 |
| CCL11 | -1.50971 | 6.210195 | -1.80203 | 0.08803 |
| F2R | -0.77302 | 7.401144 | -1.79981 | 0.088392 |
| FGF2 | -0.98225 | 3.949515 | -1.79981 | 0.088392 |
| RHOT2 | 0.46342 | 6.99775 | 1.798476 | 0.088611 |
| AMPD3 | -0.58119 | 7.437546 | -1.79667 | 0.088907 |
| MEIS2 | -0.6171 | 6.771707 | -1.79606 | 0.089006 |
| WRN | -0.46764 | 5.97137 | -1.79503 | 0.089176 |
| MPZL2 | -1.29891 | 8.060104 | -1.792 | 0.089675 |
| SLC39A6 | -0.80564 | 9.697706 | -1.78955 | 0.090081 |
| ATRX | -0.49697 | 8.08472 | -1.78627 | 0.090628 |
| KIF5B | -0.60953 | 9.254111 | -1.78472 | 0.090887 |
| PCNA | -0.66808 | 8.968049 | -1.78115 | 0.091486 |
| CPE | 1.10272 | 7.242122 | 1.779381 | 0.091784 |
| DUSP2 | 1.482448 | 5.127515 | 1.778356 | 0.091957 |
| SNAP23 | -0.44642 | 8.406728 | -1.77513 | 0.092504 |
| NUP205 | -0.57403 | 7.312935 | -1.77213 | 0.093015 |
| IL6ST | -0.50369 | 8.956551 | -1.77167 | 0.093094 |
| GCLC | -1.12079 | 8.252732 | -1.77161 | 0.093104 |
| PRKAR2B | 0.829744 | 5.76006 | 1.764874 | 0.09426 |
| CBFA2T3 | -0.83561 | 5.41682 | -1.7604 | 0.095035 |
| REEP1 | -0.57171 | 7.166429 | -1.75764 | 0.095516 |
| JMJD6 | 0.405107 | 7.435752 | 1.756596 | 0.095699 |
| KIF20A | 0.649694 | 7.146753 | 1.755038 | 0.095972 |
| PPP2R4 | 0.433026 | 7.646929 | 1.753638 | 0.096218 |
| PRDX2 | -0.8487 | 8.674979 | -1.75285 | 0.096357 |
| IFNB1 | 0.578425 | 2.052416 | 1.752273 | 0.096458 |
| LUM | -0.91027 | 10.54691 | -1.74971 | 0.09691 |
| SGCD | -1.76155 | 5.624667 | -1.74723 | 0.09735 |
| FEZ1 | -0.65714 | 7.639729 | -1.74704 | 0.097384 |
| PKIA | -1.03329 | 6.483335 | -1.74589 | 0.097588 |
| TGFB2 | -0.68431 | 5.321132 | -1.74551 | 0.097656 |
| NCAN | 0.75262 | 4.954097 | 1.742854 | 0.09813 |
| MREG | -0.8924 | 7.338382 | -1.74109 | 0.098446 |
| DLG1 | -0.67253 | 8.662962 | -1.73579 | 0.0994 |
| ABI1 | -0.6204 | 8.890453 | -1.72485 | 0.101396 |
| ATF3 | 0.678367 | 7.992954 | 1.723465 | 0.101651 |
| IL10 | 0.504239 | 5.096813 | 1.723045 | 0.101728 |
| ACE | -0.88833 | 3.327891 | -1.72132 | 0.102048 |
| MSN | 0.438745 | 10.39699 | 1.721006 | 0.102105 |
| IPO7 | -0.48175 | 8.93199 | -1.70972 | 0.104212 |
| DDIT4 | -0.64964 | 6.009627 | -1.70463 | 0.105174 |
| ANO1 | 1.346379 | 8.34603 | 1.698766 | 0.106293 |
| CASQ2 | -2.01956 | 6.544387 | -1.69804 | 0.106432 |
| IL10RB | 0.473488 | 7.073779 | 1.696782 | 0.106673 |
| KCNH2 | -0.80591 | 6.175595 | -1.6938 | 0.107247 |
| APOA1 | -1.18701 | 3.39183 | -1.6887 | 0.108237 |
| LPL | 1.393102 | 5.834869 | 1.684992 | 0.10896 |
| MYH9 | 0.604772 | 9.260762 | 1.683321 | 0.109287 |
| SDC2 | 0.907206 | 7.671586 | 1.675838 | 0.110764 |
| GADD45B | 1.163232 | 7.560279 | 1.670286 | 0.11187 |
| SCARB1 | 0.486709 | 6.68401 | 1.668473 | 0.112233 |
| NDRG1 | 1.053082 | 11.08946 | 1.666043 | 0.112722 |
| PRDX4 | 0.554826 | 9.410191 | 1.665108 | 0.112911 |
| GNPAT | -0.40666 | 8.052118 | -1.65653 | 0.114652 |
| RABGAP1L | -0.58957 | 7.031505 | -1.65593 | 0.114775 |
| ETV5 | -0.48755 | 7.051408 | -1.65414 | 0.115143 |
| CLDN6 | 0.432881 | 5.876359 | 1.653991 | 0.115172 |
| INCENP | -0.90474 | 4.664359 | -1.65381 | 0.11521 |
| DNTT | 0.568917 | 2.437046 | 1.651636 | 0.115657 |
| IRF8 | 0.700035 | 7.445296 | 1.648424 | 0.116319 |
| NPC1 | -0.60092 | 8.053903 | -1.64781 | 0.116446 |
| MYF6 | -1.58787 | 4.698113 | -1.64516 | 0.116996 |
| ADAM8 | -1.00887 | 5.962798 | -1.64463 | 0.117106 |
| KLK11 | -1.67862 | 7.994898 | -1.64178 | 0.117701 |
| TPD52 | -0.79791 | 8.269292 | -1.64119 | 0.117824 |
| PVRL3 | -0.50254 | 5.241554 | -1.64017 | 0.118038 |
| ETFB | 0.596413 | 7.369579 | 1.63962 | 0.118153 |
| GNGT1 | -1.2932 | 2.124982 | -1.63929 | 0.118223 |
| RASAL2 | 1.126929 | 3.366025 | 1.637655 | 0.118566 |
| PODXL | -0.61251 | 7.577836 | -1.6369 | 0.118724 |
| CCNG2 | -0.73647 | 7.524875 | -1.6366 | 0.118788 |
| GALNT1 | -0.5648 | 8.563501 | -1.63623 | 0.118867 |
| CCND2 | -0.87946 | 9.063478 | -1.63607 | 0.1189 |
| APP | 0.709576 | 9.244044 | 1.632594 | 0.119634 |
| EIF3A | -0.43184 | 9.47577 | -1.63227 | 0.119702 |
| NFASC | -1.09692 | 4.32193 | -1.62379 | 0.121513 |
| SCG5 | 1.188737 | 6.490684 | 1.619541 | 0.122428 |
| TMEM45A | 0.837076 | 9.42732 | 1.619511 | 0.122434 |
| FBP1 | 0.536769 | 6.505164 | 1.618739 | 0.122601 |
| CASP2 | -1.24721 | 4.509278 | -1.61533 | 0.123341 |
| PEX13 | 0.599972 | 5.94018 | 1.614946 | 0.123424 |
| SKAP2 | 0.619999 | 6.586696 | 1.614736 | 0.12347 |
| IDI1 | -0.53801 | 8.099515 | -1.61467 | 0.123485 |
| CASP3 | -0.41523 | 6.848318 | -1.60675 | 0.125219 |
| CCL19 | 1.386567 | 7.924306 | 1.606556 | 0.125261 |
| CASQ1 | -1.27312 | 6.067115 | -1.60212 | 0.126242 |
| CDC20 | 0.596891 | 6.210946 | 1.601996 | 0.126269 |
| DBF4 | -0.776 | 5.878952 | -1.59888 | 0.126962 |
| ODC1 | -1.39759 | 10.0358 | -1.59475 | 0.127885 |
| RIT1 | -0.3506 | 6.647005 | -1.59361 | 0.128142 |
| SLC37A4 | -1.59951 | 7.802708 | -1.59331 | 0.12821 |
| H1F0 | -0.53394 | 8.579074 | -1.59069 | 0.128797 |
| RPS6KA2 | -0.60834 | 6.43258 | -1.58987 | 0.128984 |
| PTPN6 | 0.473038 | 7.381032 | 1.58908 | 0.129162 |
| PICK1 | 0.514963 | 5.743745 | 1.588818 | 0.129221 |
| ALDH3B1 | 0.435415 | 6.757956 | 1.587412 | 0.129539 |
| RRAGD | -1.39191 | 6.259168 | -1.58676 | 0.129687 |
| PRMT5 | 0.675594 | 7.737805 | 1.582974 | 0.130549 |
| DONSON | -0.57988 | 6.696672 | -1.58255 | 0.130646 |
| AP2S1 | 0.399685 | 9.803477 | 1.580902 | 0.131022 |
| ARHGDIA | 0.367504 | 9.58952 | 1.580611 | 0.131088 |
| CNTFR | 0.495821 | 5.870713 | 1.579967 | 0.131236 |
| NUP98 | -0.53276 | 6.991483 | -1.57982 | 0.131271 |
| ABCG4 | 0.605357 | 5.829834 | 1.579275 | 0.131395 |
| DCBLD2 | 0.616319 | 2.213688 | 1.575264 | 0.132317 |
| TSG101 | -0.46659 | 9.064011 | -1.57493 | 0.132396 |
| SLC38A1 | -0.64164 | 8.472453 | -1.57229 | 0.133005 |
| FAM134B | -1.11336 | 6.484716 | -1.57132 | 0.133231 |
| FLNC | -1.20184 | 7.339563 | -1.5705 | 0.13342 |
| IL12A | 0.554866 | 4.692444 | 1.570369 | 0.133451 |
| PPIF | -0.65978 | 9.157599 | -1.56971 | 0.133603 |
| FCER1G | 0.595531 | 8.585017 | 1.56938 | 0.133681 |
| TIMP1 | 0.771567 | 10.83691 | 1.569179 | 0.133728 |
| PEX12 | -0.3836 | 6.596374 | -1.56873 | 0.133833 |
| FGF22 | 1.127945 | 4.284857 | 1.568326 | 0.133926 |
| MMP2 | -0.88411 | 10.41278 | -1.5664 | 0.134376 |
| CAB39L | 0.470098 | 2.173165 | 1.562145 | 0.135373 |
| INHBB | 0.81041 | 6.502925 | 1.562019 | 0.135403 |
| E2F8 | -0.53048 | 5.29593 | -1.56115 | 0.135608 |
| SLC22A18 | 0.537765 | 6.430003 | 1.561115 | 0.135616 |
| HSD11B1 | 0.6694 | 5.688405 | 1.557412 | 0.13649 |
| EXOSC8 | -0.50512 | 6.862255 | -1.55701 | 0.136586 |
| NOD1 | 0.374677 | 7.448191 | 1.556858 | 0.136622 |
| ARID4A | -0.53149 | 5.894058 | -1.55431 | 0.137226 |
| TUBB2B | 0.83962 | 4.553331 | 1.553405 | 0.137442 |
| CASP6 | 0.531098 | 5.712016 | 1.552408 | 0.13768 |
| CAPG | 0.559312 | 8.406323 | 1.550905 | 0.138039 |
| SRC | 0.914589 | 4.620728 | 1.549701 | 0.138327 |
| TRIM29 | -0.84079 | 10.58139 | -1.54501 | 0.139454 |
| TSPAN1 | -1.29664 | 6.453775 | -1.54117 | 0.140381 |
| DLG5 | -0.48876 | 8.119034 | -1.54107 | 0.140407 |
| EVL | 0.578002 | 7.282262 | 1.540475 | 0.140551 |
| CDK1 | -0.81106 | 7.579564 | -1.54024 | 0.140607 |
| LRPAP1 | 0.643651 | 7.523364 | 1.539582 | 0.140767 |
| NFIL3 | 0.482994 | 8.429251 | 1.533985 | 0.142133 |
| HMGCS2 | -0.97498 | 4.172433 | -1.53037 | 0.14302 |
| ABCA6 | -1.06064 | 3.905914 | -1.52793 | 0.143622 |
| TPM3 | -1.68851 | 6.694878 | -1.5274 | 0.143753 |
| ETV1 | -0.7849 | 5.183914 | -1.52568 | 0.14418 |
| IGFBP3 | -0.97535 | 8.999556 | -1.52567 | 0.144182 |
| DUSP1 | 0.708453 | 11.31951 | 1.525645 | 0.144188 |
| ANXA2 | 0.376766 | 12.42236 | 1.524971 | 0.144355 |
| PDGFB | 0.441447 | 6.517282 | 1.524814 | 0.144394 |
| NQO1 | -0.83729 | 8.733634 | -1.52293 | 0.144863 |
| DAPP1 | -0.56369 | 7.492863 | -1.52191 | 0.145117 |
| NDUFS2 | -0.40851 | 8.998776 | -1.52106 | 0.145327 |
| TGFB1 | 0.585246 | 8.06543 | 1.520308 | 0.145516 |
| MDK | 0.902187 | 6.52217 | 1.520095 | 0.145569 |
| FST | -0.8535 | 9.006271 | -1.51905 | 0.145831 |
| CCNT1 | 0.727417 | 5.497082 | 1.518862 | 0.145877 |
| LIF | 1.362493 | 5.220618 | 1.518031 | 0.146085 |
| SPC25 | -0.62077 | 5.577195 | -1.51469 | 0.146925 |
| GCNT2 | 0.833689 | 3.764871 | 1.513466 | 0.147233 |
| STIL | -0.52343 | 6.670785 | -1.50987 | 0.148142 |
| MEF2C | -1.04377 | 7.705327 | -1.50896 | 0.148373 |
| GNA14 | 0.776987 | 5.673063 | 1.507046 | 0.14886 |
| SDC3 | 0.379661 | 9.103376 | 1.506435 | 0.149015 |
| FADS2 | -0.91127 | 6.215902 | -1.50605 | 0.149114 |
| CD209 | 0.634156 | 7.15898 | 1.504918 | 0.149402 |
| GFRA1 | 0.562672 | 4.713759 | 1.503376 | 0.149796 |
| ENO3 | -1.3893 | 6.337027 | -1.50228 | 0.150077 |
| LDB3 | -1.39913 | 6.446645 | -1.4977 | 0.151255 |
| RXRG | 0.606438 | 5.271321 | 1.495327 | 0.151867 |
| DYNLT3 | -0.49413 | 8.736245 | -1.49523 | 0.151892 |
| DHRS2 | 0.628928 | 5.436766 | 1.494634 | 0.152047 |
| ALDOA | 0.51124 | 11.37575 | 1.490947 | 0.153004 |
| SMTN | 0.469914 | 7.670284 | 1.490234 | 0.15319 |
| MIF | 0.525499 | 10.64771 | 1.488575 | 0.153623 |
| ATP6V1H | 0.39041 | 7.88741 | 1.485719 | 0.15437 |
| ANKZF1 | 1.285556 | 4.05539 | 1.485346 | 0.154468 |
| TMEM8B | 0.459971 | 5.775873 | 1.484735 | 0.154628 |
| WEE1 | -0.74412 | 7.340397 | -1.47449 | 0.157338 |
| YTHDC1 | -0.39707 | 9.25363 | -1.47268 | 0.15782 |
| GPX4 | 0.497251 | 9.162745 | 1.470344 | 0.158446 |
| HEXA | 0.522153 | 7.93835 | 1.463726 | 0.160227 |
| TKTL1 | 1.033955 | 3.357303 | 1.462217 | 0.160636 |
| ALDH3A2 | -0.53036 | 8.335566 | -1.4619 | 0.160722 |
| CXCL1 | -1.06198 | 8.396636 | -1.46179 | 0.160752 |
| NGF | 0.784075 | 4.48838 | 1.460279 | 0.161162 |
| ANKRD2 | -1.13308 | 3.581407 | -1.4573 | 0.161972 |
| ITGB4 | 0.730224 | 9.084306 | 1.456991 | 0.162057 |
| KCNJ8 | 1.556824 | 4.981332 | 1.454357 | 0.162777 |
| GALNT3 | -0.86732 | 7.815842 | -1.45244 | 0.163304 |
| PRPF4B | -0.39119 | 7.878525 | -1.45237 | 0.163322 |
| RER1 | 0.339893 | 8.919129 | 1.451866 | 0.163461 |
| GCNT1 | 0.71509 | 5.811184 | 1.446995 | 0.164804 |
| TSC1 | -0.38702 | 7.230436 | -1.44578 | 0.165141 |
| ABCA8 | -1.57156 | 5.837346 | -1.44439 | 0.165525 |
| ANXA1 | -0.79091 | 11.65865 | -1.44219 | 0.166137 |
| APBB1 | 0.6008 | 5.977012 | 1.441221 | 0.166408 |
| SPP1 | 1.426519 | 9.399512 | 1.440987 | 0.166473 |
| IGF1 | 1.305906 | 5.744883 | 1.439247 | 0.166959 |
| CCP110 | -0.77228 | 5.719968 | -1.43828 | 0.16723 |
| PTK2 | 0.534346 | 8.401127 | 1.43737 | 0.167485 |
| RASA1 | -0.49659 | 8.746409 | -1.43716 | 0.167543 |
| ERGIC3 | 0.354353 | 9.146049 | 1.435992 | 0.167872 |
| CH25H | -0.76937 | 6.339854 | -1.43389 | 0.168463 |
| USO1 | -0.42797 | 8.563685 | -1.43308 | 0.168692 |
| IL1B | -1.17984 | 7.974264 | -1.43195 | 0.169009 |
| TFPI | -0.46708 | 6.687749 | -1.42952 | 0.169698 |
| KIF11 | -0.75799 | 6.661942 | -1.4284 | 0.170016 |
| C2 | 0.576407 | 6.667929 | 1.426112 | 0.170665 |
| PITX2 | -0.97306 | 4.823915 | -1.42564 | 0.170801 |
| GCH1 | -0.44085 | 7.378039 | -1.42217 | 0.17179 |
| SLBP | -0.36104 | 8.400478 | -1.42112 | 0.172092 |
| CLDN8 | -0.61809 | 4.724557 | -1.41988 | 0.172448 |
| HSPA4L | -0.89015 | 6.573361 | -1.41807 | 0.172966 |
| CD4 | 0.565025 | 7.674299 | 1.417086 | 0.173251 |
| ANXA9 | -0.96697 | 4.849965 | -1.41595 | 0.173577 |
| DSC3 | -1.42503 | 9.306586 | -1.41517 | 0.173803 |
| NUP50 | -0.42445 | 8.329015 | -1.41249 | 0.174578 |
| MYOM1 | -1.23986 | 5.068571 | -1.41183 | 0.174771 |
| FBXO5 | -0.72661 | 5.473351 | -1.41151 | 0.174864 |
| SMC4 | -0.70883 | 7.902679 | -1.41144 | 0.174883 |
| RPS19 | 0.38306 | 12.1045 | 1.409052 | 0.175578 |
| MON2 | -0.32285 | 7.676122 | -1.40738 | 0.176065 |
| MYOM2 | -0.89229 | 5.675046 | -1.40442 | 0.176931 |
| CDC45 | 1.103801 | 3.95348 | 1.403541 | 0.17719 |
| ITK | 0.635634 | 6.465507 | 1.401445 | 0.177806 |
| TES | -0.46692 | 8.796965 | -1.3985 | 0.178675 |
| HN1 | 0.441605 | 8.833485 | 1.396695 | 0.179208 |
| AR | 0.737639 | 5.496765 | 1.396499 | 0.179266 |
| NCAPD2 | 0.422431 | 6.981366 | 1.395789 | 0.179476 |
| CDADC1 | 0.666574 | 4.950381 | 1.395216 | 0.179646 |
| CMKLR1 | 0.689005 | 4.630692 | 1.395043 | 0.179698 |
| ELF1 | 0.347359 | 7.710002 | 1.393435 | 0.180175 |
| TLE3 | 0.459972 | 7.049938 | 1.391785 | 0.180667 |
| SGMS1 | -0.42664 | 6.941096 | -1.39168 | 0.180698 |
| EIF3J | -0.37134 | 8.332365 | -1.38365 | 0.183104 |
| DMPK | -0.6562 | 6.994437 | -1.38354 | 0.183139 |
| STX4 | 0.332773 | 7.726258 | 1.383091 | 0.183273 |
| ING3 | -0.46095 | 6.217304 | -1.38134 | 0.1838 |
| BNIP3 | -0.58638 | 7.870522 | -1.38106 | 0.183886 |
| PLAT | -0.54995 | 7.815434 | -1.37245 | 0.186504 |
| AVL9 | -0.33202 | 7.135173 | -1.37031 | 0.18716 |
| CSF2RB | -0.57402 | 7.624238 | -1.36807 | 0.187848 |
| GPI | 0.415331 | 9.475326 | 1.367516 | 0.188018 |
| MRAS | 0.499169 | 2.387902 | 1.364632 | 0.188907 |
| CDH13 | -0.77326 | 5.51165 | -1.36304 | 0.189399 |
| CAV3 | -0.60583 | 6.356613 | -1.36276 | 0.189487 |
| PPM1D | -0.32818 | 6.179315 | -1.36222 | 0.189654 |
| CD7 | 1.060725 | 3.839517 | 1.359866 | 0.190385 |
| WAS | 0.502608 | 8.402208 | 1.358114 | 0.19093 |
| IL1R1 | -0.48568 | 8.846565 | -1.35667 | 0.191379 |
| EIF3D | 0.354891 | 9.631992 | 1.353402 | 0.192402 |
| ENO1 | 0.395362 | 11.24587 | 1.349436 | 0.193649 |
| CTNNA1 | 0.33195 | 9.604255 | 1.349182 | 0.193729 |
| PIPOX | -0.38966 | 5.633034 | -1.34696 | 0.19443 |
| RAB14 | 0.39038 | 8.558265 | 1.344802 | 0.195114 |
| BARD1 | -0.50462 | 5.753784 | -1.34474 | 0.195133 |
| ACVRL1 | 0.874014 | 5.359292 | 1.34335 | 0.195574 |
| GRB2 | 0.32762 | 7.262941 | 1.342813 | 0.195745 |
| RAD51AP1 | -0.63015 | 6.132075 | -1.34223 | 0.195931 |
| FUCA1 | 0.55693 | 6.528469 | 1.339824 | 0.196696 |
| KIF23 | -0.66637 | 5.114271 | -1.33875 | 0.197039 |
| ACTG2 | 0.769918 | 7.105257 | 1.335949 | 0.197936 |
| GNAI2 | 0.508631 | 7.304064 | 1.334197 | 0.198498 |
| CD8A | 0.873464 | 6.455448 | 1.331388 | 0.199402 |
| HSPB2 | -1.27492 | 5.308222 | -1.33128 | 0.199438 |
| SMAD7 | 0.382717 | 7.504902 | 1.328979 | 0.200181 |
| ASB13 | -0.80229 | 6.46728 | -1.3279 | 0.200531 |
| COG2 | -0.28502 | 7.656034 | -1.32739 | 0.200694 |
| MSH2 | -0.49377 | 6.853001 | -1.32538 | 0.201347 |
| CDKN1A | 0.454637 | 8.98265 | 1.323129 | 0.20208 |
| TMEM176A | 0.636086 | 8.789051 | 1.319697 | 0.203201 |
| SLCO2A1 | 0.463806 | 7.397879 | 1.318515 | 0.203588 |
| CLDN15 | 0.718406 | 4.686965 | 1.31681 | 0.204148 |
| TMEM176B | 0.860111 | 8.226896 | 1.315755 | 0.204495 |
| MYL3 | -1.02255 | 5.909023 | -1.31546 | 0.204591 |
| VHL | 0.772286 | 3.271794 | 1.31536 | 0.204625 |
| ATG10 | 0.516261 | 5.629126 | 1.314897 | 0.204777 |
| KRT15 | -1.73314 | 8.910835 | -1.3139 | 0.205105 |
| RPL9 | 0.338774 | 12.34283 | 1.312994 | 0.205405 |
| LPPR4 | -0.45548 | 5.843932 | -1.31289 | 0.205439 |
| BMP1 | 0.761415 | 7.426086 | 1.312057 | 0.205714 |
| ITGAV | -0.48615 | 9.900263 | -1.31109 | 0.206033 |
| RAB9A | -0.30988 | 8.327646 | -1.31058 | 0.206203 |
| PGR | 0.555762 | 1.758469 | 1.307557 | 0.207206 |
| BIN1 | -0.75399 | 8.255615 | -1.30165 | 0.209178 |
| SLC29A1 | 0.55451 | 6.618925 | 1.301403 | 0.20926 |
| TIMP2 | 0.747313 | 6.577118 | 1.293153 | 0.212038 |
| MKNK2 | -0.50982 | 9.264649 | -1.2919 | 0.212463 |
| ACTR3 | -0.3164 | 11.36128 | -1.2883 | 0.213686 |
| EBP | 0.67007 | 6.47736 | 1.288247 | 0.213704 |
| FASN | 0.466234 | 7.506138 | 1.284433 | 0.215006 |
| RBBP7 | -0.33155 | 9.278037 | -1.283 | 0.215495 |
| EIF4E | -0.47717 | 8.177741 | -1.28197 | 0.215848 |
| RAD50 | -0.4345 | 6.487846 | -1.28196 | 0.215855 |
| GSTM1 | -0.63316 | 7.179844 | -1.28108 | 0.216157 |
| BAG1 | -0.44018 | 8.021185 | -1.28075 | 0.216269 |
| NEFH | 1.30362 | 3.119527 | 1.28056 | 0.216334 |
| ITGA4 | 0.391761 | 6.308415 | 1.280023 | 0.216519 |
| AGRN | 0.557646 | 9.006336 | 1.272512 | 0.219115 |
| NF2 | 0.492741 | 6.687525 | 1.270265 | 0.219897 |
| ARFIP1 | -0.40484 | 7.280819 | -1.26797 | 0.220697 |
| SEMA3B | 0.646239 | 3.329177 | 1.267153 | 0.220982 |
| VLDLR | -0.49501 | 6.75333 | -1.26671 | 0.221137 |
| ATAD2 | -0.61185 | 6.559117 | -1.26658 | 0.221184 |
| DHCR7 | 0.870141 | 7.32532 | 1.264419 | 0.22194 |
| SEC14L2 | 0.590528 | 5.568833 | 1.26392 | 0.222115 |
| FKBP1B | -0.69377 | 6.243715 | -1.26135 | 0.223019 |
| ADD3 | -0.59206 | 9.543677 | -1.25974 | 0.223584 |
| ID2 | 0.489166 | 8.406273 | 1.259238 | 0.223763 |
| HLA-DRA | 0.798582 | 10.46374 | 1.25917 | 0.223787 |
| CD3E | 0.361328 | 6.992037 | 1.255427 | 0.225111 |
| ADD1 | 0.393411 | 8.385669 | 1.254448 | 0.225458 |
| FOXC1 | 0.427985 | 6.130595 | 1.254326 | 0.225502 |
| SLC22A5 | -0.38087 | 6.736468 | -1.25283 | 0.226034 |
| MLPH | -0.59672 | 7.218791 | -1.25129 | 0.226581 |
| IL3RA | -0.59576 | 3.709614 | -1.24683 | 0.228175 |
| TAOK2 | 0.398237 | 6.30242 | 1.246149 | 0.22842 |
| CSNK2B | 0.356956 | 9.328375 | 1.242834 | 0.229611 |
| RGS16 | 0.678844 | 6.840663 | 1.240432 | 0.230478 |
| PLVAP | 0.488349 | 7.555125 | 1.239081 | 0.230966 |
| ATF5 | 0.479481 | 6.482025 | 1.236936 | 0.231743 |
| MVD | -0.96764 | 3.106998 | -1.22795 | 0.235019 |
| EPHX2 | -0.89085 | 3.568951 | -1.22745 | 0.235202 |
| UGP2 | -0.39579 | 9.588077 | -1.2274 | 0.23522 |
| STAT1 | -0.46103 | 10.39331 | -1.22473 | 0.236201 |
| KIF22 | 0.913183 | 5.117543 | 1.223524 | 0.236646 |
| ADRA1B | 0.688498 | 3.153686 | 1.22346 | 0.23667 |
| GUCY1A3 | -0.42943 | 7.776442 | -1.22224 | 0.237121 |
| CCL7 | 0.784195 | 4.281359 | 1.219595 | 0.238098 |
| TSPAN7 | -0.56093 | 7.394704 | -1.21687 | 0.239109 |
| CTSS | 0.619215 | 8.010718 | 1.213949 | 0.240195 |
| CSK | 0.308645 | 7.797507 | 1.213284 | 0.240443 |
| MICB | -0.51011 | 6.674177 | -1.21281 | 0.24062 |
| TSC2 | 0.565022 | 6.242081 | 1.208574 | 0.242205 |
| HRAS | -0.44656 | 7.943972 | -1.20845 | 0.242252 |
| MSRA | -0.38293 | 6.626442 | -1.2075 | 0.242609 |
| TTK | -0.68105 | 6.688984 | -1.20558 | 0.243331 |
| IL10RA | 0.502051 | 7.535134 | 1.200415 | 0.245281 |
| TXNRD1 | -0.4422 | 8.63058 | -1.19969 | 0.245556 |
| PTBP2 | -0.37393 | 6.575317 | -1.19724 | 0.246487 |
| JAG2 | 0.556325 | 8.860443 | 1.194091 | 0.247684 |
| GLRX2 | -0.33082 | 7.756292 | -1.19271 | 0.248211 |
| F2 | 0.625413 | 3.272678 | 1.192496 | 0.248294 |
| SLIT2 | 0.523217 | 6.304941 | 1.187908 | 0.250053 |
| PPP1R15A | 0.451471 | 7.801136 | 1.184741 | 0.251272 |
| IKZF1 | 0.398341 | 6.773621 | 1.183869 | 0.251609 |
| CCL22 | -0.59424 | 6.411038 | -1.18341 | 0.251785 |
| PPP1R3C | -1.10941 | 6.401533 | -1.18014 | 0.253051 |
| SLC23A2 | 0.457339 | 6.256334 | 1.179455 | 0.253317 |
| IL9R | 0.505215 | 5.631472 | 1.176726 | 0.254378 |
| MYH7 | -0.82597 | 6.248877 | -1.1751 | 0.255011 |
| CDH1 | -0.73401 | 10.00866 | -1.1701 | 0.256969 |
| LTB | 0.748672 | 5.912818 | 1.169811 | 0.257081 |
| SH3BP5 | -0.57052 | 8.154763 | -1.16828 | 0.257683 |
| FKBP5 | 1.273187 | 3.955588 | 1.16801 | 0.257789 |
| TBC1D30 | -0.41009 | 5.277054 | -1.16798 | 0.257801 |
| DNAJA1 | -0.36078 | 9.312642 | -1.16581 | 0.258655 |
| SFPQ | 0.319038 | 9.435923 | 1.164872 | 0.259025 |
| IFNAR2 | -0.26779 | 7.454771 | -1.16349 | 0.25957 |
| MT2A | 0.37865 | 12.13908 | 1.160722 | 0.260667 |
| UNG | -0.44255 | 7.325117 | -1.16038 | 0.260801 |
| SYT12 | 0.683601 | 5.014721 | 1.157091 | 0.26211 |
| DSC1 | -1.3152 | 6.025635 | -1.15467 | 0.263073 |
| CD3D | 0.547576 | 7.282073 | 1.154253 | 0.263241 |
| PTTG3P | -0.91208 | 5.224879 | -1.15222 | 0.264053 |
| KLF10 | 0.373498 | 9.727083 | 1.150251 | 0.264844 |
| MAP4K1 | 1.009015 | 3.67508 | 1.149453 | 0.265164 |
| MAP3K7 | -0.42963 | 7.384355 | -1.14588 | 0.266601 |
| ACSL1 | -0.51018 | 8.920881 | -1.14551 | 0.266753 |
| TGM2 | 0.459494 | 8.334699 | 1.144986 | 0.266963 |
| TSPAN4 | 0.578529 | 6.414503 | 1.144041 | 0.267345 |
| CYP7B1 | -0.42967 | 5.696751 | -1.14395 | 0.26738 |
| IFNGR1 | -0.33392 | 9.858873 | -1.14356 | 0.267539 |
| HPRT1 | 0.733205 | 6.33216 | 1.140818 | 0.268649 |
| XPO1 | -0.31858 | 9.816743 | -1.14079 | 0.268662 |
| CNN2 | 0.286013 | 8.39325 | 1.140076 | 0.26895 |
| BRCA1 | -0.78076 | 5.583665 | -1.13981 | 0.269059 |
| SPDEF | -0.63141 | 6.710436 | -1.13812 | 0.269744 |
| SMARCC1 | -0.29915 | 7.968473 | -1.13695 | 0.270222 |
| HAX1 | 0.338104 | 8.203783 | 1.13525 | 0.270915 |
| TRIB2 | -0.41101 | 7.39618 | -1.13447 | 0.271235 |
| SPI1 | 0.564436 | 5.500101 | 1.13271 | 0.271953 |
| TMPO | -0.43591 | 7.012183 | -1.13066 | 0.272794 |
| EXO1 | -0.58282 | 6.049454 | -1.12959 | 0.273233 |
| CDC25A | 0.540986 | 4.821492 | 1.128081 | 0.273853 |
| STMN1 | 0.816052 | 6.607533 | 1.126733 | 0.274407 |
| CD40LG | 0.739851 | 3.423388 | 1.125304 | 0.274997 |
| BCL11B | 0.492948 | 7.017924 | 1.124466 | 0.275343 |
| CADM3 | -0.59177 | 7.055177 | -1.12275 | 0.276052 |
| KIF15 | -0.42564 | 5.931201 | -1.12102 | 0.27677 |
| TYK2 | -0.48288 | 6.972139 | -1.12026 | 0.277082 |
| MYO1C | -0.3411 | 8.617767 | -1.12005 | 0.277173 |
| LEF1 | -0.43383 | 7.153612 | -1.11967 | 0.277328 |
| SGK1 | 0.499053 | 10.24012 | 1.11956 | 0.277375 |
| HBEGF | 0.793593 | 7.700029 | 1.119045 | 0.277589 |
| NCF4 | 0.382535 | 6.678334 | 1.117907 | 0.278062 |
| YWHAH | 0.305019 | 8.714007 | 1.114423 | 0.279514 |
| CLDN14 | 0.766424 | 4.934431 | 1.113434 | 0.279927 |
| NAV2 | -0.5383 | 7.808537 | -1.11295 | 0.280129 |
| BTG3 | -0.37293 | 8.638004 | -1.11123 | 0.280852 |
| MAPKAP1 | 0.314042 | 7.0389 | 1.111104 | 0.280903 |
| CDC7 | -0.38018 | 6.964096 | -1.11009 | 0.281329 |
| ADAM17 | -0.46197 | 7.326721 | -1.10927 | 0.281671 |
| HOXB9 | 0.42177 | 4.621616 | 1.108885 | 0.281834 |
| RHOD | 0.398542 | 9.738992 | 1.108632 | 0.281941 |
| MAPK8 | 0.6272 | 4.597139 | 1.108332 | 0.282067 |
| GPC3 | -0.56766 | 6.531527 | -1.10756 | 0.282392 |
| GUCY2D | 0.522492 | 5.04856 | 1.106188 | 0.282969 |
| GBE1 | -0.34646 | 8.190752 | -1.1051 | 0.283427 |
| HLA-DMA | 0.515151 | 8.599263 | 1.104384 | 0.28373 |
| TRAIP | -0.52819 | 4.44499 | -1.10289 | 0.28436 |
| KLF6 | 0.486822 | 8.258967 | 1.102045 | 0.284719 |
| CCL2 | 1.397307 | 7.192377 | 1.101134 | 0.285105 |
| ACTN4 | 0.366863 | 9.437582 | 1.100806 | 0.285244 |
| SOCS5 | -0.31973 | 6.943137 | -1.1005 | 0.285376 |
| DHX16 | 0.290144 | 7.455221 | 1.099786 | 0.285677 |
| COX7A1 | -0.82076 | 7.130752 | -1.09792 | 0.286469 |
| PRKG2 | 0.712358 | 5.356222 | 1.096632 | 0.287018 |
| SOD1 | 0.331649 | 10.38255 | 1.096384 | 0.287123 |
| SMAD3 | -0.76073 | 6.561112 | -1.08448 | 0.292226 |
| PEG3 | -0.38718 | 5.804478 | -1.08332 | 0.292728 |
| DTYMK | 0.454447 | 5.798331 | 1.083158 | 0.292798 |
| IL15 | -0.40831 | 6.606921 | -1.08297 | 0.292881 |
| ACHE | -0.79462 | 3.887642 | -1.08101 | 0.293726 |
| DNAJC12 | -0.8314 | 4.897881 | -1.07664 | 0.295623 |
| CSF2RA | -0.7431 | 4.506842 | -1.07627 | 0.295785 |
| CCRN4L | 0.407204 | 6.099997 | 1.076142 | 0.295841 |
| MTIF2 | -0.57647 | 7.529889 | -1.07603 | 0.29589 |
| C3AR1 | 0.469552 | 7.09833 | 1.073287 | 0.297086 |
| PYGM | -1.13092 | 5.915546 | -1.07314 | 0.29715 |
| CAV2 | -0.51628 | 9.203308 | -1.07246 | 0.297448 |
| ADAM15 | 0.708942 | 4.803137 | 1.072283 | 0.297525 |
| BATF | 0.648849 | 6.062659 | 1.069501 | 0.298742 |
| NCK1 | -0.39271 | 8.070363 | -1.06939 | 0.298792 |
| TSPAN13 | -0.45092 | 8.830966 | -1.06902 | 0.298954 |
| SH3BGR | -1.25477 | 4.331277 | -1.06825 | 0.299289 |
| AKAP1 | -0.31483 | 8.381559 | -1.06644 | 0.300088 |
| CYP26B1 | -0.40008 | 6.97136 | -1.06438 | 0.300993 |
| CXCR3 | 0.615198 | 4.836501 | 1.063457 | 0.301401 |
| RFC3 | -0.41293 | 7.228739 | -1.06272 | 0.301726 |
| SPARCL1 | -0.58006 | 9.853613 | -1.06227 | 0.301924 |
| CD74 | 0.553671 | 11.42231 | 1.06137 | 0.302323 |
| H2AFX | -0.46039 | 7.97204 | -1.06133 | 0.302342 |
| CFL1 | 0.26764 | 12.68228 | 1.061057 | 0.302462 |
| LTF | -1.60535 | 4.090286 | -1.0585 | 0.303594 |
| MTMR10 | 0.680342 | 4.778114 | 1.057716 | 0.303942 |
| RACGAP1 | -0.41811 | 7.678693 | -1.0569 | 0.304303 |
| CHST3 | 0.678626 | 6.917986 | 1.055759 | 0.304812 |
| GADD45A | -0.37876 | 8.479744 | -1.05476 | 0.305258 |
| COL17A1 | 1.190251 | 8.704767 | 1.051083 | 0.306897 |
| LDHC | 0.458596 | 5.054289 | 1.049531 | 0.307591 |
| LLGL2 | -0.40508 | 5.860996 | -1.04902 | 0.30782 |
| LCP1 | 0.432197 | 7.997655 | 1.049004 | 0.307827 |
| STAM2 | -0.66667 | 5.861561 | -1.0475 | 0.308501 |
| SLC20A1 | 0.39267 | 8.647284 | 1.047048 | 0.308705 |
| CXCL14 | -0.64624 | 10.6961 | -1.04633 | 0.309025 |
| THY1 | 0.578791 | 8.115308 | 1.045282 | 0.309498 |
| SLC2A3 | 0.793541 | 7.317684 | 1.04464 | 0.309787 |
| TNFRSF12A | 0.495537 | 8.052802 | 1.043202 | 0.310435 |
| XPNPEP1 | -0.25484 | 8.566974 | -1.04178 | 0.311076 |
| SLC19A2 | -0.35388 | 6.843895 | -1.04115 | 0.31136 |
| RRAS | 0.50767 | 6.739256 | 1.040218 | 0.311781 |
| M6PR | 0.549865 | 7.138239 | 1.037151 | 0.313171 |
| PHYH | -0.48552 | 6.820296 | -1.03469 | 0.31429 |
| SGCG | -1.25386 | 4.203367 | -1.03389 | 0.314654 |
| ABHD2 | 0.588501 | 6.94717 | 1.030212 | 0.316329 |
| FAM102A | 0.462701 | 7.765784 | 1.028629 | 0.317053 |
| SLC12A2 | -0.39466 | 7.28862 | -1.02732 | 0.317651 |
| SRSF1 | 0.362276 | 8.91945 | 1.026923 | 0.317834 |
| FSTL1 | -0.41498 | 10.65872 | -1.02629 | 0.318124 |
| PIN1 | -0.76093 | 5.579071 | -1.02561 | 0.318437 |
| MCM6 | -0.39053 | 8.588016 | -1.02546 | 0.318505 |
| CASP4 | -0.34106 | 6.73014 | -1.02495 | 0.318741 |
| SBNO2 | 0.571046 | 6.656849 | 1.024858 | 0.318781 |
| HLA-DMB | 0.690066 | 8.402236 | 1.024742 | 0.318835 |
| HMGN2 | 0.246334 | 11.1297 | 1.02469 | 0.318859 |
| HHEX | 0.366401 | 6.043093 | 1.024478 | 0.318956 |
| DPYSL4 | -0.53274 | 4.833545 | -1.01941 | 0.321291 |
| ETS1 | 0.524912 | 5.479548 | 1.017721 | 0.322072 |
| COL16A1 | 0.517309 | 7.80532 | 1.017642 | 0.322108 |
| TNFRSF1B | 0.532318 | 6.866335 | 1.017562 | 0.322145 |
| AURKB | 0.460315 | 6.315715 | 1.01677 | 0.322512 |
| TFPI2 | 0.914448 | 2.993251 | 1.015558 | 0.323074 |
| GADD45G | 0.771268 | 5.20412 | 1.014273 | 0.32367 |
| IL16 | 0.280535 | 6.925125 | 1.014051 | 0.323773 |
| KLK10 | -1.29944 | 9.384104 | -1.01213 | 0.324666 |
| PRDM1 | -0.4962 | 6.138374 | -1.00949 | 0.325898 |
| SORBS3 | 0.804873 | 5.750472 | 1.008866 | 0.326187 |
| CUL4A | -0.48961 | 8.034988 | -1.00747 | 0.32684 |
| BCL2L2 | -0.23654 | 7.849977 | -1.00684 | 0.327134 |
| CFB | -0.48176 | 7.619597 | -1.00209 | 0.329362 |
| IL13RA1 | -0.31241 | 8.613511 | -1.00161 | 0.329585 |
| FXYD1 | -0.70495 | 6.48771 | -0.99763 | 0.331463 |
| EPHB3 | 0.541906 | 6.817077 | 0.997622 | 0.331466 |
| BCL10 | -0.28761 | 8.111362 | -0.99404 | 0.333163 |
| GYPC | 0.432699 | 7.034124 | 0.993119 | 0.333597 |
| IGSF1 | -0.69305 | 4.292704 | -0.99279 | 0.333755 |
| WISP2 | 0.959937 | 4.984531 | 0.990934 | 0.334634 |
| CTH | 0.412518 | 4.754444 | 0.990921 | 0.334641 |
| SS18 | -0.40884 | 6.653793 | -0.98671 | 0.336648 |
| VASP | 0.386295 | 7.361838 | 0.985661 | 0.337147 |
| ZNF292 | -0.38505 | 7.398706 | -0.98483 | 0.337542 |
| PF4 | 0.489207 | 5.564109 | 0.980972 | 0.339393 |
| PLXNB1 | 0.737826 | 4.948514 | 0.98087 | 0.339442 |
| IL1R2 | -1.23749 | 6.48681 | -0.98068 | 0.339534 |
| CROT | -0.40105 | 7.265213 | -0.97932 | 0.340187 |
| CDK2 | -0.33152 | 7.719908 | -0.97908 | 0.340301 |
| PLCB2 | 0.466966 | 5.031004 | 0.978993 | 0.340344 |
| GZMB | -0.41705 | 6.293984 | -0.97838 | 0.340636 |
| G0S2 | 0.633188 | 7.96512 | 0.977694 | 0.340969 |
| ITGA2 | 0.442452 | 7.210438 | 0.977076 | 0.341266 |
| ACTN3 | -0.49949 | 5.749759 | -0.97376 | 0.342867 |
| MAFB | -0.41662 | 9.311791 | -0.97317 | 0.34315 |
| P4HA1 | 0.360429 | 8.123701 | 0.970579 | 0.344407 |
| ORC5 | -0.29679 | 7.297584 | -0.97002 | 0.344678 |
| PRNP | -0.40613 | 10.81129 | -0.96833 | 0.345497 |
| IL2 | 0.902172 | 2.418968 | 0.967514 | 0.345895 |
| GALK1 | -0.31952 | 2.298566 | -0.96706 | 0.346117 |
| ERBB3 | -0.43467 | 7.541068 | -0.96692 | 0.346183 |
| LARGE | -0.27007 | 6.768225 | -0.96675 | 0.346267 |
| PEX7 | -0.24944 | 6.657181 | -0.96612 | 0.346572 |
| TFF1 | -0.60109 | 5.299911 | -0.96508 | 0.347078 |
| MYC | 0.409863 | 9.091664 | 0.964431 | 0.347397 |
| SCAF4 | -0.38206 | 6.114579 | -0.96435 | 0.347437 |
| EFS | 0.311778 | 7.00841 | 0.962942 | 0.348124 |
| CENPE | -0.96488 | 5.27145 | -0.96249 | 0.348343 |
| OXSR1 | -0.27336 | 7.756247 | -0.96119 | 0.348981 |
| TAGLN | 0.710298 | 9.402159 | 0.957956 | 0.350565 |
| NMU | -1.08942 | 4.998343 | -0.95595 | 0.351549 |
| TGFBR3 | -0.4826 | 6.391162 | -0.95332 | 0.352847 |
| TFAP2C | -0.42793 | 8.408137 | -0.95069 | 0.354146 |
| TFDP1 | -0.4735 | 8.745372 | -0.94921 | 0.354878 |
| XBP1 | -0.47585 | 9.082646 | -0.94718 | 0.35588 |
| LCK | 0.63391 | 6.350396 | 0.942128 | 0.358394 |
| FOXO4 | 0.585437 | 4.039726 | 0.940032 | 0.359439 |
| IFITM3 | -0.48929 | 11.34395 | -0.93919 | 0.359857 |
| NME1 | 0.310168 | 9.595396 | 0.93877 | 0.36007 |
| AKT1 | 0.29085 | 7.882283 | 0.938729 | 0.36009 |
| CYP4F11 | -0.88878 | 2.339307 | -0.93761 | 0.36065 |
| TNF | -0.47724 | 4.942375 | -0.93739 | 0.36076 |
| FGF6 | 0.481392 | 4.183678 | 0.935979 | 0.361467 |
| DOCK2 | 0.906171 | 4.880132 | 0.935006 | 0.361955 |
| SNX10 | -0.51045 | 6.534689 | -0.93348 | 0.362722 |
| PTPRR | -0.41509 | 4.561406 | -0.93311 | 0.362907 |
| CLTA | -0.24338 | 10.04529 | -0.93282 | 0.363053 |
| CSE1L | -0.24875 | 9.144536 | -0.93272 | 0.363101 |
| IGFBP1 | 0.57815 | 4.056471 | 0.931069 | 0.363934 |
| SNRPD1 | -0.3586 | 8.785456 | -0.93075 | 0.364094 |
| PFN1 | 0.367012 | 10.94197 | 0.930171 | 0.364386 |
| KAZN | -0.45181 | 7.569791 | -0.93003 | 0.364457 |
| LRIG1 | -0.55861 | 7.147571 | -0.92995 | 0.364496 |
| COL5A1 | 0.69999 | 9.870809 | 0.926237 | 0.366372 |
| TXN | -0.39693 | 10.67491 | -0.92585 | 0.366566 |
| BRS3 | 0.319978 | 4.852648 | 0.92397 | 0.36752 |
| ZYX | 0.342958 | 8.627306 | 0.922545 | 0.368243 |
| BCL2L11 | 0.546135 | 4.354554 | 0.92252 | 0.368256 |
| VPS4B | -0.36837 | 8.782629 | -0.92229 | 0.368373 |
| IL18R1 | -0.52634 | 4.713648 | -0.92209 | 0.368475 |
| ICAM2 | 0.57177 | 6.680382 | 0.920668 | 0.369197 |
| ADAMDEC1 | 0.528072 | 7.04973 | 0.918784 | 0.370155 |
| NRAP | -0.8505 | 5.513986 | -0.9182 | 0.370451 |
| ISG20L2 | 0.205155 | 7.610938 | 0.913467 | 0.37287 |
| G3BP1 | 0.287279 | 8.554811 | 0.91121 | 0.374027 |
| CD79A | 0.558676 | 6.339649 | 0.91099 | 0.374139 |
| MEF2A | -0.31388 | 8.288637 | -0.91025 | 0.374517 |
| AKT2 | 0.823079 | 4.077925 | 0.910202 | 0.374544 |
| FOSL2 | 0.438526 | 7.417845 | 0.909725 | 0.374789 |
| TLR8 | -0.603 | 2.155682 | -0.90863 | 0.375354 |
| PKP1 | -1.25677 | 9.372401 | -0.90744 | 0.375965 |
| NRTN | 0.708489 | 0.940026 | 0.907023 | 0.376179 |
| CA2 | 0.800706 | 7.503582 | 0.905437 | 0.376996 |
| ALDH8A1 | -0.40147 | 3.897204 | -0.90523 | 0.377103 |
| BAX | -0.4966 | 4.47194 | -0.90501 | 0.377216 |
| TOB1 | -0.32974 | 8.611757 | -0.90403 | 0.377722 |
| KPNA2 | -0.34623 | 9.593157 | -0.90363 | 0.377926 |
| CAMK4 | 0.63931 | 4.622693 | 0.903606 | 0.377941 |
| BGN | 0.54779 | 9.495724 | 0.901318 | 0.379124 |
| MBP | 0.425853 | 6.874228 | 0.900499 | 0.379547 |
| NF1 | 0.401055 | 4.175313 | 0.900476 | 0.379559 |
| AMMECR1 | -0.4426 | 7.445886 | -0.89981 | 0.379904 |
| SOD3 | -0.39167 | 6.85352 | -0.89936 | 0.380136 |
| ADAM23 | -0.37671 | 6.458855 | -0.89705 | 0.381336 |
| SMAD2 | -0.31345 | 8.176284 | -0.89656 | 0.381591 |
| CKB | 0.459846 | 7.277714 | 0.895197 | 0.382301 |
| PTPRC | -0.43818 | 7.915232 | -0.89474 | 0.382537 |
| PDGFA | -0.36447 | 6.691134 | -0.89453 | 0.382648 |
| SAP30 | -0.28958 | 6.591099 | -0.89402 | 0.382914 |
| HLA-E | 0.264151 | 11.10148 | 0.892579 | 0.383665 |
| G6PD | -0.44339 | 6.96113 | -0.88916 | 0.385453 |
| CXCL6 | -0.5742 | 5.805899 | -0.88894 | 0.385568 |
| AQP3 | -0.786 | 10.57559 | -0.88845 | 0.385825 |
| PDZK1 | -0.47381 | 4.560952 | -0.88733 | 0.386408 |
| PGAM2 | -0.90568 | 3.140402 | -0.88662 | 0.38678 |
| NCAM1 | -0.2937 | 6.395332 | -0.88624 | 0.386979 |
| CCR2 | -0.3356 | 6.224669 | -0.88536 | 0.387444 |
| CALCR | 0.410786 | 5.841171 | 0.884401 | 0.387947 |
| COPE | 0.292053 | 8.469705 | 0.88191 | 0.389257 |
| DST | -0.55436 | 9.514744 | -0.88173 | 0.38935 |
| SRSF10 | -0.23272 | 7.83226 | -0.87971 | 0.390417 |
| ATXN1 | -0.33126 | 8.049935 | -0.87841 | 0.391106 |
| FYB | 0.423261 | 6.19731 | 0.876015 | 0.392371 |
| ITGB3 | 0.400525 | 5.900648 | 0.8757 | 0.392537 |
| HSD3B1 | 0.574478 | 2.447921 | 0.871784 | 0.394615 |
| IL12B | 0.409089 | 4.521904 | 0.870624 | 0.395232 |
| CD86 | 0.340641 | 6.893847 | 0.869965 | 0.395583 |
| XIAP | 0.740957 | 4.6025 | 0.869269 | 0.395953 |
| CAV1 | -0.41235 | 10.71675 | -0.86793 | 0.396668 |
| WNT7A | 0.508697 | 5.643415 | 0.866198 | 0.397591 |
| RETN | -0.62796 | 2.97808 | -0.86595 | 0.397724 |
| POLD2 | -0.25647 | 7.392338 | -0.86582 | 0.397792 |
| NUSAP1 | -0.40481 | 8.306045 | -0.8653 | 0.398072 |
| VAMP7 | -0.46921 | 5.415024 | -0.85718 | 0.402426 |
| UGCG | 0.440658 | 8.674259 | 0.856596 | 0.402741 |
| SETD8 | -0.38281 | 5.403471 | -0.85651 | 0.402786 |
| HADH | -0.39041 | 6.986162 | -0.85579 | 0.403178 |
| RPS6KA3 | -0.27808 | 7.510148 | -0.85556 | 0.403299 |
| PSEN1 | 0.239767 | 7.568662 | 0.852569 | 0.404914 |
| NEDD4L | -0.36489 | 6.880449 | -0.85075 | 0.405896 |
| MED24 | 0.378124 | 3.719093 | 0.850712 | 0.405919 |
| BID | -0.31655 | 7.337052 | -0.8482 | 0.407277 |
| DTNA | -0.36001 | 5.854266 | -0.84775 | 0.407522 |
| ITGB2 | -0.23686 | 8.589859 | -0.84748 | 0.407669 |
| MYOG | -0.48902 | 3.776393 | -0.84462 | 0.409226 |
| ADAM10 | -0.35174 | 9.170842 | -0.8439 | 0.409619 |
| SPTAN1 | 0.301315 | 8.30385 | 0.841576 | 0.410883 |
| MGMT | 0.345287 | 6.337062 | 0.840228 | 0.41162 |
| IL9 | 0.590557 | 3.955071 | 0.840052 | 0.411716 |
| PEX1 | -0.20687 | 5.997866 | -0.83989 | 0.411806 |
| CTSC | 0.37285 | 10.02071 | 0.839779 | 0.411865 |
| PA2G4 | 0.28161 | 8.758641 | 0.838967 | 0.412309 |
| GJA1 | 0.413804 | 10.77952 | 0.838082 | 0.412793 |
| MELK | -0.40831 | 8.136016 | -0.83609 | 0.413882 |
| NUP107 | -0.21691 | 8.167694 | -0.83564 | 0.414131 |
| CHST2 | -0.38142 | 7.054096 | -0.83497 | 0.4145 |
| IPCEF1 | 0.332979 | 5.288219 | 0.833972 | 0.415046 |
| CREBBP | -0.18244 | 7.934846 | -0.83204 | 0.416108 |
| PIKFYVE | -0.28494 | 6.573233 | -0.83147 | 0.416422 |
| AURKA | 0.372913 | 6.989669 | 0.831053 | 0.416651 |
| MCM4 | 0.287024 | 8.302911 | 0.83051 | 0.41695 |
| TXNIP | -0.42748 | 10.39605 | -0.82775 | 0.41847 |
| MLYCD | -0.56572 | 5.377653 | -0.82747 | 0.418629 |
| NOLC1 | -0.20273 | 8.797528 | -0.82714 | 0.418809 |
| KDM3A | -0.27363 | 8.067321 | -0.82637 | 0.419234 |
| GNAI1 | -0.37145 | 7.223009 | -0.82629 | 0.419277 |
| CENPM | -0.59804 | 5.152627 | -0.82233 | 0.421468 |
| AKR1D1 | 0.350256 | 4.226963 | 0.821881 | 0.421719 |
| AQP9 | -0.87196 | 5.479044 | -0.82163 | 0.42186 |
| PURA | -0.26551 | 8.18394 | -0.82155 | 0.421904 |
| UBE2N | -0.21086 | 9.319278 | -0.8209 | 0.422267 |
| IDS | -0.28521 | 8.075137 | -0.81934 | 0.423132 |
| ILF3 | -0.30214 | 8.397244 | -0.8176 | 0.4241 |
| DUSP6 | 0.358238 | 8.841997 | 0.817026 | 0.424419 |
| CDCA8 | 0.757685 | 5.653896 | 0.81697 | 0.424449 |
| PRKDC | 0.351842 | 8.378009 | 0.816366 | 0.424786 |
| EMP2 | -0.3414 | 8.523648 | -0.81527 | 0.425396 |
| HSD17B4 | 0.22007 | 8.159003 | 0.813855 | 0.426187 |
| PXMP2 | -0.33231 | 6.608972 | -0.81365 | 0.426301 |
| CD247 | 0.434209 | 5.95446 | 0.805791 | 0.430706 |
| FLT4 | 0.584837 | 3.915128 | 0.805191 | 0.431043 |
| SEC24D | -0.35141 | 7.159007 | -0.80505 | 0.431125 |
| RAD54L | -0.5422 | 4.43567 | -0.80351 | 0.43199 |
| CDSN | -0.96319 | 5.107862 | -0.80229 | 0.432675 |
| BNIP3L | 0.309768 | 8.528789 | 0.802204 | 0.432725 |
| HSPB1 | 0.309504 | 11.5228 | 0.801185 | 0.4333 |
| CASP9 | 0.238962 | 7.352231 | 0.801036 | 0.433384 |
| NDST1 | 0.358555 | 6.800393 | 0.798522 | 0.434804 |
| NAGK | 0.238107 | 8.39199 | 0.798239 | 0.434965 |
| ITPK1 | 0.211261 | 7.826743 | 0.79798 | 0.435111 |
| TPI1 | 0.252726 | 10.93592 | 0.795133 | 0.436723 |
| CTNNB1 | -0.26719 | 8.851067 | -0.79512 | 0.436733 |
| INSIG1 | -0.32949 | 7.456466 | -0.79481 | 0.436909 |
| WWTR1 | 0.349399 | 7.79116 | 0.793287 | 0.437772 |
| SYMPK | 0.460498 | 4.569387 | 0.792905 | 0.437988 |
| MAPK14 | 0.243941 | 7.146407 | 0.792813 | 0.43804 |
| DIAPH3 | -0.68292 | 3.133271 | -0.79251 | 0.438215 |
| MCM5 | 0.24852 | 7.819549 | 0.79215 | 0.438417 |
| MYL6B | -0.40762 | 7.951323 | -0.79178 | 0.43863 |
| STAT3 | 0.197538 | 10.06916 | 0.791576 | 0.438744 |
| DMP1 | 0.783471 | 1.013882 | 0.791273 | 0.438916 |
| CTCF | 0.178908 | 8.234513 | 0.791009 | 0.439066 |
| ITGA9 | 0.323673 | 5.221257 | 0.787799 | 0.440895 |
| CHEK2 | -0.24424 | 6.549938 | -0.78766 | 0.440974 |
| GRHPR | -0.24704 | 7.681777 | -0.78757 | 0.441024 |
| GINS2 | -0.51928 | 6.29124 | -0.78685 | 0.441435 |
| MYBL2 | 0.474864 | 6.350792 | 0.786572 | 0.441596 |
| DNAJC3 | -0.43619 | 4.90022 | -0.78501 | 0.442486 |
| YKT6 | -0.37306 | 7.069888 | -0.78391 | 0.443115 |
| AKT3 | -0.18319 | 7.824552 | -0.78268 | 0.443824 |
| XRCC6 | 0.18672 | 10.34453 | 0.781215 | 0.444661 |
| MYLK | 0.454099 | 8.36069 | 0.781046 | 0.444758 |
| PLK1 | 0.276781 | 6.617319 | 0.780739 | 0.444934 |
| TLR2 | -0.46893 | 7.267674 | -0.78058 | 0.445028 |
| RANBP1 | 0.26013 | 8.729543 | 0.780227 | 0.445228 |
| IL1A | -0.64645 | 7.01158 | -0.77909 | 0.445878 |
| EDN2 | 0.713938 | 3.559421 | 0.777611 | 0.446731 |
| ITGA7 | -0.99389 | 5.450365 | -0.77682 | 0.447188 |
| NCOR2 | 0.270483 | 8.888638 | 0.775645 | 0.447863 |
| IL13 | 0.403863 | 4.923669 | 0.772296 | 0.449794 |
| CDKN1B | -0.27501 | 8.457119 | -0.77221 | 0.449847 |
| TSPO | 0.209414 | 9.180016 | 0.771826 | 0.450066 |
| CBL | 0.390578 | 4.294542 | 0.768776 | 0.45183 |
| MT1E | 0.323699 | 10.78257 | 0.768279 | 0.452118 |
| CACNA1H | 0.353985 | 3.120007 | 0.767157 | 0.452768 |
| AGR2 | -1.07695 | 5.438908 | -0.76715 | 0.452773 |
| GALE | 0.24787 | 6.29738 | 0.766692 | 0.453038 |
| ALDH1A3 | -0.53726 | 7.650918 | -0.76197 | 0.45578 |
| KATNA1 | 0.173525 | 7.319224 | 0.761616 | 0.455989 |
| AMIGO2 | -0.54631 | 8.201455 | -0.76099 | 0.456352 |
| CAP1 | -0.23357 | 10.45841 | -0.75984 | 0.457025 |
| NOTCH1 | 0.293393 | 6.813032 | 0.759721 | 0.457093 |
| CSF3R | 0.62875 | 4.793428 | 0.758406 | 0.457861 |
| LOX | 0.448738 | 7.69844 | 0.757934 | 0.458136 |
| PCDH1 | 0.452029 | 5.761883 | 0.756528 | 0.458958 |
| EGFR | 0.500255 | 9.376942 | 0.756225 | 0.459135 |
| TNFRSF11B | -0.76988 | 2.314693 | -0.75606 | 0.459234 |
| CD44 | 0.367954 | 10.0243 | 0.756045 | 0.45924 |
| NUMA1 | -0.28762 | 7.40431 | -0.75251 | 0.461311 |
| MYOF | -0.23713 | 9.770509 | -0.75181 | 0.461721 |
| ERBB2 | -0.51134 | 7.794383 | -0.7516 | 0.461847 |
| HSPB8 | -0.50674 | 8.562198 | -0.75072 | 0.462359 |
| SATB1 | -0.25008 | 7.50568 | -0.75057 | 0.462452 |
| NIN | 0.46091 | 3.41235 | 0.750149 | 0.462697 |
| MED13L | -0.22453 | 7.691259 | -0.74888 | 0.463443 |
| SIRPA | 0.281807 | 7.816659 | 0.748875 | 0.463446 |
| PAK4 | 0.421834 | 5.961045 | 0.748523 | 0.463653 |
| APLNR | 0.5071 | 5.554696 | 0.745406 | 0.465489 |
| STX7 | -0.23887 | 7.479552 | -0.74501 | 0.465725 |
| GTF2F1 | 0.213452 | 7.253502 | 0.74274 | 0.467063 |
| TACC3 | 0.365897 | 6.209372 | 0.742233 | 0.467363 |
| CRAT | -0.35154 | 6.166611 | -0.74191 | 0.467551 |
| ARPC2 | 0.166924 | 10.93789 | 0.740624 | 0.468315 |
| MAD2L1 | -0.41399 | 7.720779 | -0.73979 | 0.468807 |
| PEX11A | -0.38427 | 4.890422 | -0.73921 | 0.46915 |
| GCK | 0.353927 | 2.717488 | 0.737049 | 0.470433 |
| KLF5 | -0.47531 | 8.896303 | -0.73564 | 0.471268 |
| FASLG | 0.236477 | 5.469306 | 0.734385 | 0.472016 |
| NOTCH2 | 0.236477 | 9.145191 | 0.733793 | 0.472368 |
| PEA15 | -0.21263 | 9.711192 | -0.73245 | 0.473166 |
| BCAR3 | 0.300671 | 6.046338 | 0.731751 | 0.473584 |
| KLRD1 | -0.33329 | 5.704871 | -0.7301 | 0.474566 |
| FOXO3 | 0.217598 | 7.847029 | 0.729605 | 0.474864 |
| TIMP3 | 0.551273 | 8.978675 | 0.729456 | 0.474952 |
| IL12RB1 | 0.539286 | 4.228703 | 0.729055 | 0.475192 |
| MEF2D | 0.492564 | 4.600937 | 0.728845 | 0.475317 |
| TJP3 | 0.415734 | 3.596241 | 0.727883 | 0.475892 |
| SSPN | -0.4692 | 6.53158 | -0.72776 | 0.475963 |
| MPZL1 | 0.172595 | 8.880061 | 0.727739 | 0.475978 |
| LBR | -0.28665 | 8.516721 | -0.72727 | 0.476258 |
| CIDEA | 0.380111 | 5.229004 | 0.726686 | 0.476608 |
| KIF18B | -0.25809 | 7.064558 | -0.72599 | 0.477022 |
| TMPRSS3 | 0.275935 | 5.119716 | 0.719152 | 0.481126 |
| FSCN1 | 0.428388 | 8.115691 | 0.718709 | 0.481393 |
| COL6A3 | -0.35637 | 11.60236 | -0.71838 | 0.481588 |
| SOX3 | 0.479799 | 4.013339 | 0.717949 | 0.48185 |
| STAG1 | -0.27958 | 6.862718 | -0.71223 | 0.485298 |
| OLFML3 | 0.504729 | 7.998298 | 0.710193 | 0.486533 |
| TRAT1 | -0.54249 | 3.17772 | -0.70878 | 0.487388 |
| GPX1 | 0.30003 | 9.300875 | 0.707653 | 0.488072 |
| HOXC10 | 0.490157 | 6.359996 | 0.707414 | 0.488217 |
| LAMB3 | 0.446562 | 9.699088 | 0.70654 | 0.488748 |
| CTF1 | 0.380285 | 4.39507 | 0.70641 | 0.488827 |
| MEST | 0.374252 | 8.386818 | 0.705827 | 0.489181 |
| ATP1A1 | 0.251731 | 9.925837 | 0.703464 | 0.490617 |
| TRA2B | -0.176 | 9.866753 | -0.70145 | 0.491845 |
| PRDX6 | 0.248048 | 9.206163 | 0.701091 | 0.492062 |
| AMD1 | -0.20938 | 7.851839 | -0.70024 | 0.492582 |
| CD2 | 0.365221 | 7.08043 | 0.69996 | 0.492752 |
| ZW10 | -0.23018 | 7.593616 | -0.69974 | 0.492887 |
| SLCO1A2 | 0.279079 | 5.084498 | 0.696605 | 0.4948 |
| MPP5 | -0.24472 | 5.857486 | -0.69634 | 0.494965 |
| ACVR2A | -0.19237 | 7.047774 | -0.6957 | 0.495354 |
| MYL9 | 0.65055 | 8.168722 | 0.695403 | 0.495535 |
| OSMR | 0.231432 | 6.670732 | 0.694444 | 0.496123 |
| GBF1 | 0.177157 | 7.377688 | 0.694231 | 0.496253 |
| GLRX | -0.36834 | 8.37987 | -0.69238 | 0.497388 |
| ISG20 | 0.928641 | 5.144149 | 0.690574 | 0.498496 |
| KIF5C | 0.239639 | 5.960096 | 0.689398 | 0.499218 |
| SUV39H1 | 0.187161 | 6.902477 | 0.689093 | 0.499406 |
| DIABLO | 0.163696 | 8.56762 | 0.688645 | 0.499681 |
| CASP1 | -0.28646 | 8.230025 | -0.68844 | 0.499806 |
| TMX1 | -0.26744 | 9.444794 | -0.68818 | 0.499968 |
| CD38 | 0.32043 | 6.440979 | 0.688075 | 0.500032 |
| DAPK2 | -0.34651 | 5.399752 | -0.68732 | 0.500495 |
| KPNB1 | 0.201134 | 9.863359 | 0.685964 | 0.501331 |
| APOD | -0.59776 | 8.702356 | -0.68412 | 0.502466 |
| RFC1 | -0.20679 | 7.012765 | -0.68353 | 0.502831 |
| IL15RA | 0.171049 | 7.299756 | 0.679435 | 0.505362 |
| MARCKS | 0.190145 | 10.25417 | 0.677885 | 0.506322 |
| NOS1 | -0.24676 | 6.232737 | -0.67748 | 0.506573 |
| PAPSS2 | -0.33648 | 7.66492 | -0.67654 | 0.507155 |
| HMGB2 | -0.23821 | 9.162294 | -0.67124 | 0.510451 |
| SCUBE2 | -0.25635 | 5.709276 | -0.66964 | 0.511446 |
| FLNB | 0.324752 | 7.168304 | 0.669541 | 0.511507 |
| TAP1 | -0.26632 | 8.649958 | -0.66931 | 0.511648 |
| PRKAG1 | -0.16942 | 7.894829 | -0.66854 | 0.512131 |
| BCAP31 | 0.195533 | 8.976297 | 0.668204 | 0.51234 |
| TPX2 | 0.303846 | 8.286367 | 0.667098 | 0.51303 |
| HS3ST1 | -0.69544 | 4.490976 | -0.66637 | 0.513484 |
| GABARAPL2 | -0.18768 | 9.524336 | -0.66636 | 0.513489 |
| KLHL24 | 0.327204 | 6.783923 | 0.665186 | 0.514225 |
| HLA-DQA1 | -1.18542 | 5.740097 | -0.6649 | 0.514402 |
| MCL1 | 0.181551 | 10.66582 | 0.664046 | 0.514937 |
| DHCR24 | -0.30899 | 9.00677 | -0.66355 | 0.515248 |
| NDC80 | -0.40231 | 5.444645 | -0.66287 | 0.515674 |
| KCNK5 | 0.215117 | 5.259935 | 0.661826 | 0.516327 |
| EPHB2 | 0.338698 | 5.865843 | 0.661521 | 0.516518 |
| MYH11 | 0.878296 | 5.370569 | 0.661454 | 0.51656 |
| CHAF1A | -0.27195 | 6.357518 | -0.66138 | 0.516605 |
| WARS | 0.261346 | 9.239503 | 0.660037 | 0.517448 |
| CCNA2 | 0.306869 | 7.07799 | 0.658744 | 0.51826 |
| MAP3K8 | -0.26267 | 5.320386 | -0.65753 | 0.519024 |
| YWHAB | -0.17953 | 10.88453 | -0.65672 | 0.519531 |
| LAT2 | 0.256986 | 6.19402 | 0.656502 | 0.519668 |
| MSMB | 0.779676 | 3.512978 | 0.655711 | 0.520166 |
| TK1 | 0.216849 | 7.147199 | 0.655133 | 0.520529 |
| IMPA2 | -0.36633 | 8.368314 | -0.6543 | 0.521053 |
| CDH4 | -0.38258 | 5.62289 | -0.65308 | 0.521825 |
| LAMA3 | 0.482082 | 9.535028 | 0.652622 | 0.522111 |
| F2RL1 | 0.272291 | 7.882941 | 0.652097 | 0.522442 |
| GYS1 | -0.17347 | 8.139959 | -0.65099 | 0.52314 |
| TNPO2 | -0.2325 | 7.966584 | -0.65003 | 0.523746 |
| TNNT2 | -0.29804 | 5.159625 | -0.64975 | 0.523921 |
| CDKN3 | 0.270801 | 7.646264 | 0.649635 | 0.523995 |
| RAN | 0.206065 | 10.63466 | 0.649519 | 0.524069 |
| RIPK1 | 0.147907 | 6.627188 | 0.649347 | 0.524178 |
| CDC27 | 0.243744 | 7.050436 | 0.648824 | 0.524508 |
| KLF1 | 0.255214 | 4.810893 | 0.648073 | 0.524983 |
| IL7R | 0.257794 | 7.81233 | 0.648022 | 0.525015 |
| ACTB | 0.171047 | 13.37855 | 0.646588 | 0.525922 |
| PLAUR | 0.28298 | 8.556917 | 0.645555 | 0.526576 |
| EIF2S1 | -0.25552 | 9.026059 | -0.64516 | 0.526826 |
| SCAMP3 | 0.137351 | 7.989265 | 0.644319 | 0.52736 |
| IL6 | 0.487161 | 7.928764 | 0.643645 | 0.527787 |
| STC2 | -0.61381 | 4.313185 | -0.64364 | 0.527791 |
| STAT4 | 0.186493 | 6.688329 | 0.642349 | 0.528609 |
| PIK3CB | -0.16011 | 6.887606 | -0.64175 | 0.528986 |
| RB1 | -0.22519 | 7.830568 | -0.63954 | 0.530396 |
| CKS2 | -0.32945 | 8.406446 | -0.63928 | 0.530562 |
| YIPF6 | -0.16864 | 7.768492 | -0.63899 | 0.53074 |
| BRCA2 | -0.26988 | 5.255039 | -0.63633 | 0.532436 |
| RHOBTB3 | 0.374438 | 7.544014 | 0.633717 | 0.534104 |
| E2F1 | -0.23881 | 7.282201 | -0.63349 | 0.534249 |
| MTHFD2 | -0.20652 | 8.7077 | -0.63293 | 0.534605 |
| CD63 | 0.186317 | 11.2667 | 0.632084 | 0.535146 |
| DUT | -0.1847 | 9.819883 | -0.63077 | 0.535985 |
| TRAF2 | -0.33949 | 3.673148 | -0.63002 | 0.536468 |
| STC1 | 0.228858 | 6.955418 | 0.629561 | 0.53676 |
| CCL20 | 0.937019 | 4.961576 | 0.628355 | 0.537532 |
| F3 | -0.44362 | 8.927312 | -0.62742 | 0.538129 |
| RAD23B | -0.1695 | 9.401182 | -0.62542 | 0.539412 |
| CTGF | 0.439733 | 9.466828 | 0.622078 | 0.541561 |
| ITGBL1 | -0.69331 | 5.461363 | -0.62196 | 0.541637 |
| NEK2 | -0.42683 | 6.111307 | -0.62121 | 0.542117 |
| TPBG | -0.23066 | 9.594075 | -0.62055 | 0.542545 |
| MSX1 | 0.265674 | 6.321609 | 0.619602 | 0.543154 |
| MALL | -0.41393 | 8.262127 | -0.61941 | 0.543281 |
| GAPDH | 0.181623 | 12.98449 | 0.61813 | 0.544103 |
| BMP2 | -0.33677 | 6.914562 | -0.61806 | 0.544148 |
| PVALB | -0.33597 | 3.747203 | -0.61788 | 0.544262 |
| PAN2 | 0.221843 | 5.631283 | 0.617676 | 0.544396 |
| HIST1H2BB | 0.307505 | 1.41903 | 0.616925 | 0.54488 |
| EFNA1 | 0.271331 | 7.836411 | 0.614618 | 0.546371 |
| PEX19 | 0.266413 | 6.614961 | 0.614129 | 0.546687 |
| GNAO1 | 0.208695 | 5.78117 | 0.613737 | 0.54694 |
| NFKBIB | 0.255532 | 5.968649 | 0.612434 | 0.547783 |
| CHEK1 | -0.26916 | 6.015993 | -0.61147 | 0.548408 |
| SIT1 | 0.272767 | 4.5167 | 0.611159 | 0.548608 |
| ARPC3 | -0.16574 | 9.585645 | -0.6109 | 0.548775 |
| PTPN2 | -0.192 | 8.154345 | -0.60984 | 0.54946 |
| SLC9A3R1 | -0.22406 | 8.245655 | -0.60952 | 0.549672 |
| ATP2B4 | 0.162347 | 8.42603 | 0.608656 | 0.55023 |
| RBM14 | 0.338315 | 6.663109 | 0.608558 | 0.550294 |
| MADD | 0.151303 | 7.233728 | 0.608376 | 0.550412 |
| NR3C2 | 0.451153 | 4.289759 | 0.608346 | 0.550431 |
| KCNN4 | 0.198555 | 6.361986 | 0.607496 | 0.550983 |
| STX16 | 0.192471 | 7.652247 | 0.607424 | 0.55103 |
| HCLS1 | 0.286505 | 8.289857 | 0.607145 | 0.551211 |
| ITGA3 | 0.334547 | 7.829234 | 0.60636 | 0.551721 |
| DNMBP | -0.18344 | 7.061886 | -0.60576 | 0.552113 |
| SYNGR1 | 0.367073 | 5.515079 | 0.605512 | 0.552272 |
| CYP27A1 | -0.45556 | 3.57195 | -0.60468 | 0.552816 |
| RAPGEFL1 | -0.6216 | 5.833236 | -0.60379 | 0.553394 |
| CDC6 | -0.20368 | 6.247417 | -0.60309 | 0.553849 |
| FGF9 | -0.41258 | 5.06441 | -0.6025 | 0.554233 |
| SNX24 | -0.26421 | 6.013356 | -0.60185 | 0.554652 |
| PC | 0.229736 | 6.479294 | 0.600789 | 0.555346 |
| PLA2G2A | 0.884425 | 7.959031 | 0.600371 | 0.555619 |
| CBX8 | 0.306753 | 3.627328 | 0.599676 | 0.556072 |
| NBL1 | 0.228973 | 8.223079 | 0.599588 | 0.556129 |
| SLC30A3 | 0.209793 | 3.168205 | 0.599579 | 0.556135 |
| TIPARP | 0.285041 | 8.56971 | 0.596173 | 0.558359 |
| CDH6 | -0.23276 | 5.407492 | -0.5957 | 0.558667 |
| IRAK4 | -0.17383 | 6.188464 | -0.59196 | 0.561117 |
| SCRN1 | 0.244333 | 8.30848 | 0.590173 | 0.562289 |
| HDAC5 | 0.168668 | 6.657556 | 0.589888 | 0.562476 |
| SNAP91 | -0.38187 | 3.94699 | -0.5889 | 0.563127 |
| DIO1 | -0.20028 | 4.38251 | -0.58876 | 0.563214 |
| KIF1B | -0.22668 | 7.066588 | -0.58592 | 0.565085 |
| ALDOB | 0.268912 | 6.128519 | 0.584479 | 0.566031 |
| CXCL12 | -0.59993 | 8.192681 | -0.58195 | 0.567696 |
| PPBP | -0.67171 | 3.998409 | -0.58184 | 0.567769 |
| FLII | -0.19319 | 8.176716 | -0.58154 | 0.567969 |
| PSMB10 | 0.216884 | 6.957621 | 0.580709 | 0.568517 |
| ANP32E | -0.2041 | 8.467114 | -0.58045 | 0.56869 |
| ADCY9 | 0.482013 | 5.469078 | 0.578798 | 0.569778 |
| KCNK15 | 0.495181 | 2.208202 | 0.578213 | 0.570165 |
| CCNB2 | 0.27983 | 8.153866 | 0.577659 | 0.570531 |
| ADORA2B | -0.28785 | 7.520608 | -0.57712 | 0.570887 |
| CSRP2 | -0.28912 | 8.295562 | -0.5763 | 0.571429 |
| PTGIS | 0.442193 | 6.85255 | 0.575986 | 0.571638 |
| VCAN | -0.40254 | 10.28317 | -0.57557 | 0.571912 |
| TOP1 | -0.14204 | 8.685436 | -0.57522 | 0.572142 |
| CYP7A1 | 0.56002 | 2.404829 | 0.57389 | 0.573026 |
| MYBBP1A | -0.16066 | 7.230581 | -0.57368 | 0.573165 |
| DCXR | 0.673665 | 6.503203 | 0.573559 | 0.573245 |
| ACTR2 | 0.167846 | 9.354067 | 0.572113 | 0.574204 |
| CDCA3 | -0.37289 | 5.975097 | -0.57187 | 0.574368 |
| RRM2 | -0.34191 | 8.353819 | -0.57165 | 0.574511 |
| BCL2L1 | -0.17611 | 7.439098 | -0.57112 | 0.57486 |
| PEX6 | -0.24196 | 7.028312 | -0.57112 | 0.574866 |
| LIPE | 0.245253 | 5.226964 | 0.571066 | 0.574898 |
| PCP4 | -0.41736 | 3.568823 | -0.57007 | 0.57556 |
| AP2B1 | 0.178038 | 7.726759 | 0.56901 | 0.576264 |
| ADAM9 | -0.194 | 8.492878 | -0.56887 | 0.576358 |
| SIAH2 | -0.41423 | 7.215442 | -0.56876 | 0.57643 |
| PECAM1 | -0.24141 | 8.73334 | -0.56837 | 0.576691 |
| CLTC | 0.113752 | 10.9467 | 0.565447 | 0.578634 |
| GJA5 | 0.309086 | 3.573542 | 0.564777 | 0.579081 |
| RFC2 | 0.179933 | 6.811554 | 0.564754 | 0.579096 |
| FGR | -0.30772 | 5.967406 | -0.56341 | 0.579994 |
| WWC1 | -0.19187 | 6.955376 | -0.56285 | 0.580365 |
| FDPS | -0.18144 | 8.048528 | -0.56279 | 0.580407 |
| TSPAN8 | 0.599004 | 5.283343 | 0.562163 | 0.580823 |
| PRG2 | 0.287191 | 4.558141 | 0.561341 | 0.581372 |
| RAB31 | 0.218142 | 9.524523 | 0.561324 | 0.581383 |
| PPARGC1A | -0.44741 | 4.365904 | -0.56117 | 0.581485 |
| KIF4A | 0.199822 | 7.033143 | 0.559736 | 0.582444 |
| IL33 | -0.3798 | 5.312075 | -0.5594 | 0.582667 |
| ELOVL5 | -0.21125 | 9.027481 | -0.55699 | 0.584283 |
| IL1RL2 | 0.42238 | 3.733513 | 0.556108 | 0.58487 |
| JAK1 | 0.169979 | 9.552039 | 0.555298 | 0.585413 |
| CXCL3 | -0.30703 | 5.321745 | -0.55434 | 0.586054 |
| PEX16 | 0.151475 | 8.155729 | 0.553856 | 0.586379 |
| TBRG4 | 0.288586 | 4.822767 | 0.553628 | 0.586532 |
| WFS1 | 0.14449 | 7.267556 | 0.55347 | 0.586638 |
| ACTN1 | 0.221878 | 10.7 | 0.551455 | 0.587989 |
| PLIN2 | 0.197895 | 7.526382 | 0.551245 | 0.588131 |
| PRRX1 | -0.24001 | 8.078587 | -0.55017 | 0.588854 |
| PGM1 | -0.22075 | 8.910763 | -0.5496 | 0.589235 |
| HLA-DOB | 0.469626 | 4.814691 | 0.549536 | 0.589279 |
| ST14 | 0.187725 | 7.418947 | 0.548781 | 0.589786 |
| RAB22A | -0.14958 | 7.868526 | -0.54846 | 0.59 |
| ST6GALNAC2 | 0.328027 | 7.553055 | 0.548331 | 0.590089 |
| AKAP12 | 0.277052 | 7.513677 | 0.547432 | 0.590694 |
| NOS2 | 0.220824 | 5.52681 | 0.54719 | 0.590857 |
| HMOX1 | -0.22911 | 7.440344 | -0.54604 | 0.591634 |
| PNRC1 | 0.312187 | 8.486003 | 0.544922 | 0.592384 |
| OLFM1 | 0.30317 | 6.405504 | 0.54435 | 0.59277 |
| MCM2 | 0.236061 | 8.496163 | 0.54327 | 0.593497 |
| FBN1 | -0.43202 | 9.528687 | -0.5428 | 0.593815 |
| PARVA | 0.412802 | 6.425202 | 0.541642 | 0.594596 |
| ARHGEF6 | 0.212406 | 6.980524 | 0.540252 | 0.595535 |
| LMAN1 | -0.33797 | 5.352323 | -0.53953 | 0.596024 |
| ADAM12 | 0.424231 | 7.70553 | 0.538912 | 0.596441 |
| PCSK1N | -0.31961 | 4.323441 | -0.53797 | 0.59708 |
| E2F2 | 0.1972 | 2.565396 | 0.537526 | 0.597378 |
| CDH3 | 0.330642 | 9.871639 | 0.532612 | 0.600708 |
| SCD | 0.313916 | 8.372242 | 0.531895 | 0.601194 |
| OLR1 | -0.3223 | 6.113297 | -0.53186 | 0.60122 |
| IL18RAP | -0.26612 | 5.110121 | -0.53094 | 0.60184 |
| DNAJC1 | 0.142364 | 6.917643 | 0.530625 | 0.602056 |
| FCGR2B | -0.24189 | 6.682668 | -0.52975 | 0.602653 |
| SLC7A1 | -0.24145 | 9.676795 | -0.52953 | 0.6028 |
| TMED10 | -0.15634 | 9.774062 | -0.52714 | 0.604429 |
| MYBL1 | 0.205068 | 5.17028 | 0.527058 | 0.604481 |
| DCN | -0.39356 | 10.32666 | -0.52689 | 0.604593 |
| RHOF | 0.204968 | 6.27105 | 0.526529 | 0.604842 |
| LTBR | 0.160337 | 7.4657 | 0.526442 | 0.604901 |
| ICA1 | 0.189783 | 5.778841 | 0.525516 | 0.605531 |
| DNM1L | -0.25717 | 7.239013 | -0.52507 | 0.605833 |
| ORC6 | -0.22316 | 7.119795 | -0.52414 | 0.606467 |
| TMEM100 | 0.293113 | 4.979142 | 0.523997 | 0.606567 |
| NCR1 | -0.30356 | 4.124162 | -0.52327 | 0.607065 |
| SIRT2 | 0.155778 | 7.74323 | 0.521713 | 0.608125 |
| STAB1 | 0.197954 | 8.387972 | 0.519563 | 0.609593 |
| SERPINA3 | 0.408555 | 8.205858 | 0.518915 | 0.610036 |
| CCL5 | -0.454 | 7.292757 | -0.51876 | 0.610145 |
| HAO1 | 0.293974 | 4.673071 | 0.517392 | 0.611077 |
| TPM2 | -0.38617 | 8.836785 | -0.51639 | 0.611766 |
| GSK3B | 0.194625 | 7.278177 | 0.516214 | 0.611884 |
| PML | 0.236524 | 7.685068 | 0.515749 | 0.612202 |
| MXI1 | -0.1733 | 8.655057 | -0.51473 | 0.612897 |
| ITPR2 | 0.251234 | 5.675263 | 0.51425 | 0.613229 |
| CFLAR | 0.255465 | 8.580961 | 0.512697 | 0.614294 |
| CUL3 | -0.1327 | 8.910865 | -0.51258 | 0.614372 |
| GSN | 0.204898 | 9.965531 | 0.512447 | 0.614465 |
| NAPA | 0.164637 | 7.98559 | 0.511987 | 0.61478 |
| DCK | -0.18757 | 6.471741 | -0.5117 | 0.614979 |
| PVRL2 | 0.178158 | 7.516181 | 0.511167 | 0.615343 |
| DSCC1 | -0.25679 | 5.428801 | -0.51068 | 0.615679 |
| INHBA | 0.416229 | 8.592983 | 0.510654 | 0.615696 |
| PDZD3 | 0.247807 | 3.527814 | 0.510574 | 0.615751 |
| RASGRP1 | -0.30993 | 6.001319 | -0.51055 | 0.615764 |
| CAB39 | -0.15595 | 8.983702 | -0.50702 | 0.618195 |
| CASP7 | -0.13459 | 7.600704 | -0.5064 | 0.618622 |
| LY75 | -0.18627 | 6.800294 | -0.50615 | 0.618791 |
| ELF4 | -0.18769 | 7.640076 | -0.50543 | 0.619285 |
| KIFC3 | -0.21966 | 6.294659 | -0.50539 | 0.619314 |
| VEGFA | 0.223912 | 8.692406 | 0.503999 | 0.620273 |
| HOMER2 | -0.24634 | 6.972637 | -0.50325 | 0.620789 |
| CDH15 | -0.22306 | 6.062944 | -0.50211 | 0.621574 |
| CFP | 0.217993 | 1.553307 | 0.501311 | 0.622126 |
| TOR1AIP2 | 0.167148 | 7.202943 | 0.501023 | 0.622325 |
| POLA2 | -0.26185 | 5.492361 | -0.49844 | 0.624106 |
| BCAT1 | 0.254161 | 4.959299 | 0.498419 | 0.624123 |
| ICAM1 | -0.16715 | 8.066771 | -0.497 | 0.625107 |
| EMP1 | -0.22348 | 9.75731 | -0.4961 | 0.625725 |
| IRF9 | 0.142661 | 8.956096 | 0.494594 | 0.626769 |
| PSMC3IP | 0.205639 | 6.56801 | 0.493681 | 0.627401 |
| GNAS | 0.19207 | 10.86099 | 0.493432 | 0.627574 |
| JAM3 | -0.18968 | 7.5299 | -0.49318 | 0.627746 |
| TRAF1 | 0.141542 | 6.269047 | 0.492705 | 0.628078 |
| TNFSF10 | 0.264929 | 10.59802 | 0.492433 | 0.628266 |
| S100A9 | -0.50365 | 11.43225 | -0.4922 | 0.628428 |
| ALDOC | -0.19635 | 7.221698 | -0.49131 | 0.629047 |
| NR1H4 | -0.40797 | 3.904044 | -0.4911 | 0.629194 |
| NAP1L2 | 0.268169 | 5.210902 | 0.490803 | 0.629397 |
| RIPK2 | -0.17565 | 7.753162 | -0.49025 | 0.629782 |
| CCL13 | -0.32814 | 6.595656 | -0.49024 | 0.629785 |
| TOP2A | -0.21404 | 7.900173 | -0.48974 | 0.630137 |
| RSU1 | 0.157189 | 8.088021 | 0.488738 | 0.63083 |
| DEPDC1 | -0.19205 | 5.842733 | -0.48843 | 0.631043 |
| ASF1B | -0.19954 | 7.488197 | -0.48814 | 0.631246 |
| AREG | 0.442927 | 7.78558 | 0.48595 | 0.632768 |
| SCN1B | -0.21696 | 6.276102 | -0.48583 | 0.632849 |
| IL17RB | -0.26164 | 5.597363 | -0.48581 | 0.632863 |
| CD40 | -0.14653 | 8.082337 | -0.48466 | 0.633664 |
| PPT1 | 0.126068 | 9.649608 | 0.483736 | 0.634309 |
| KIF2C | 0.190649 | 7.859707 | 0.483344 | 0.634581 |
| PHKG1 | -0.18452 | 5.669464 | -0.483 | 0.634818 |
| ASF1A | -0.23121 | 6.675022 | -0.48224 | 0.635354 |
| RARA | 0.362475 | 5.619509 | 0.481341 | 0.635977 |
| FARP1 | -0.20578 | 7.124229 | -0.48128 | 0.636017 |
| CCNA1 | -0.39118 | 6.32705 | -0.48063 | 0.63647 |
| RNASEH2A | -0.16138 | 7.600752 | -0.48033 | 0.636684 |
| HIRA | 0.159988 | 6.138467 | 0.480227 | 0.636754 |
| CALB2 | -0.30133 | 6.677238 | -0.48011 | 0.636838 |
| VAMP3 | 0.123193 | 9.563529 | 0.479654 | 0.637154 |
| ST6GAL1 | 0.218567 | 7.605207 | 0.478808 | 0.637744 |
| KRT19 | -0.92975 | 5.63135 | -0.47713 | 0.638916 |
| SAT1 | -0.14366 | 11.47564 | -0.47607 | 0.639654 |
| HNRNPU | 0.107208 | 10.10324 | 0.476032 | 0.639682 |
| BAIAP2 | -0.15798 | 7.107539 | -0.47494 | 0.640447 |
| EWSR1 | -0.10339 | 8.645802 | -0.4735 | 0.641454 |
| TFF3 | -0.40952 | 6.336065 | -0.47338 | 0.641537 |
| PSEN2 | 0.256931 | 5.511287 | 0.473304 | 0.641591 |
| ICAM5 | -0.29949 | 4.116877 | -0.47276 | 0.641969 |
| IL4R | -0.11786 | 8.39375 | -0.47157 | 0.642803 |
| RBP1 | -0.28473 | 8.532945 | -0.47024 | 0.643733 |
| UBE2C | 0.173578 | 8.471728 | 0.469363 | 0.644352 |
| COL15A1 | -0.31653 | 8.723275 | -0.46749 | 0.645667 |
| MPO | 0.235605 | 5.467643 | 0.467093 | 0.645944 |
| CDKN1C | -0.23733 | 7.133342 | -0.46694 | 0.646051 |
| KRT31 | 0.201861 | 5.593587 | 0.465434 | 0.647109 |
| S100A4 | -0.18208 | 9.150282 | -0.46445 | 0.647803 |
| ETF1 | 0.134676 | 8.347536 | 0.462702 | 0.64903 |
| ADCY1 | 0.281116 | 5.28892 | 0.461805 | 0.649661 |
| HSP90B1 | 0.298164 | 7.513627 | 0.460923 | 0.650282 |
| SNAP25 | -0.19503 | 6.406769 | -0.46036 | 0.650682 |
| AGXT | -0.328 | 4.94122 | -0.46029 | 0.650725 |
| TMED2 | -0.11498 | 10.57273 | -0.46017 | 0.650815 |
| DDX39A | 0.16013 | 8.971488 | 0.460101 | 0.650861 |
| FOXN3 | 0.216218 | 7.391779 | 0.45969 | 0.651151 |
| VWF | 0.174571 | 8.303881 | 0.459018 | 0.651624 |
| CLCN3 | -0.15521 | 8.515339 | -0.45897 | 0.651661 |
| TRIB3 | -0.13339 | 7.253538 | -0.45755 | 0.652658 |
| SH2B1 | 0.200453 | 5.816929 | 0.457426 | 0.652746 |
| UBE2D3 | 0.115605 | 10.34239 | 0.457325 | 0.652818 |
| CHRNG | -0.20849 | 5.445923 | -0.45588 | 0.653839 |
| HLA-DOA | -0.15421 | 6.669819 | -0.45459 | 0.654748 |
| CAMK2B | 0.143185 | 6.958444 | 0.454361 | 0.65491 |
| GOLGA4 | -0.26628 | 6.472389 | -0.45387 | 0.655259 |
| SSRP1 | -0.1327 | 7.879773 | -0.45365 | 0.65541 |
| CD96 | 0.164247 | 4.787607 | 0.453057 | 0.655831 |
| EIF4G3 | 0.116415 | 7.196718 | 0.452644 | 0.656123 |
| ODF2 | 0.274654 | 2.492087 | 0.452133 | 0.656484 |
| LIG1 | 0.127065 | 6.461418 | 0.45164 | 0.656833 |
| IGFBP6 | 0.236269 | 8.349055 | 0.451622 | 0.656846 |
| A2M | -0.23006 | 10.16544 | -0.45028 | 0.657793 |
| ACOX2 | 0.27494 | 4.758234 | 0.450078 | 0.657938 |
| ISOC1 | -0.14725 | 7.016943 | -0.4491 | 0.658627 |
| COPB2 | -0.12011 | 8.780937 | -0.44891 | 0.658763 |
| CD1D | 0.172741 | 4.739795 | 0.447665 | 0.659647 |
| NAPG | -0.18973 | 6.9675 | -0.44654 | 0.660447 |
| CLDN18 | 0.28375 | 3.327532 | 0.443475 | 0.662618 |
| SOX9 | 0.201638 | 8.04116 | 0.442338 | 0.663426 |
| SDC4 | 0.209229 | 9.657528 | 0.442143 | 0.663564 |
| CLDN5 | 0.380762 | 5.572949 | 0.441804 | 0.663805 |
| HMOX2 | 0.143824 | 7.652643 | 0.441232 | 0.664212 |
| RALB | -0.11448 | 8.421916 | -0.44103 | 0.664357 |
| GINS1 | -0.223 | 7.272219 | -0.4396 | 0.665372 |
| SFN | 0.236604 | 13.28067 | 0.439406 | 0.66551 |
| IDH1 | -0.11563 | 8.62693 | -0.43677 | 0.667385 |
| INHA | 0.147022 | 5.146362 | 0.436619 | 0.667494 |
| PDE4DIP | -0.48346 | 6.200628 | -0.43555 | 0.668254 |
| MMP10 | -0.44265 | 8.366486 | -0.43531 | 0.668424 |
| HLA-G | 0.149641 | 11.49878 | 0.433638 | 0.669618 |
| RPL3L | 0.410131 | 4.161578 | 0.432767 | 0.670239 |
| TIAM1 | 0.196806 | 7.976872 | 0.432362 | 0.670528 |
| PPP2R5B | -0.11633 | 6.243246 | -0.43223 | 0.670624 |
| CDH8 | 0.21312 | 5.761824 | 0.431093 | 0.671434 |
| LMNB1 | 0.170805 | 6.111021 | 0.431039 | 0.671473 |
| SPHK1 | -0.17787 | 8.535473 | -0.4298 | 0.672355 |
| RAC1 | -0.18322 | 10.69134 | -0.42977 | 0.672376 |
| PIK3R5 | 0.136574 | 6.829613 | 0.428611 | 0.673207 |
| TPH1 | 0.281425 | 0.58577 | 0.428066 | 0.673597 |
| RPA1 | -0.18052 | 8.141627 | -0.42728 | 0.674162 |
| EFNA3 | -0.37065 | 5.639357 | -0.42552 | 0.675417 |
| CFD | -0.59283 | 6.288983 | -0.42549 | 0.67544 |
| NDST2 | 0.123569 | 6.802133 | 0.424445 | 0.676187 |
| SERPINA1 | 0.237798 | 7.730241 | 0.424053 | 0.676468 |
| AMACR | -0.31602 | 1.324949 | -0.42335 | 0.676971 |
| SLC1A4 | -0.12823 | 8.215725 | -0.42247 | 0.677599 |
| RAD21 | -0.1565 | 9.215394 | -0.422 | 0.677938 |
| CXCL11 | -0.28726 | 6.054162 | -0.42144 | 0.678338 |
| BUB1B | -0.19843 | 7.184605 | -0.421 | 0.678658 |
| FRK | -0.37192 | 3.687545 | -0.41753 | 0.681148 |
| TTR | 0.316292 | 3.834334 | 0.417032 | 0.681504 |
| NAB2 | 0.129056 | 6.494231 | 0.416755 | 0.681703 |
| NR0B2 | 0.21062 | 4.398184 | 0.415432 | 0.682654 |
| GPR124 | 0.178506 | 6.660024 | 0.415198 | 0.682822 |
| SERPINA6 | -0.27973 | 4.085436 | -0.41514 | 0.682867 |
| ABL1 | 0.148872 | 7.980853 | 0.414864 | 0.683062 |
| EGF | -0.2264 | 5.123611 | -0.41387 | 0.683776 |
| LDLRAP1 | -0.104 | 7.515683 | -0.4135 | 0.684045 |
| IRF7 | -0.15675 | 8.352485 | -0.41299 | 0.684413 |
| LIMA1 | -0.13574 | 9.84848 | -0.41123 | 0.685675 |
| CBX5 | -0.11094 | 7.588935 | -0.41106 | 0.685797 |
| KRT8 | 0.281284 | 7.308333 | 0.411046 | 0.68581 |
| SLC7A2 | 0.342253 | 3.589989 | 0.410142 | 0.686462 |
| GSPT1 | -0.12958 | 8.303683 | -0.40994 | 0.686606 |
| RORA | 0.232781 | 5.605972 | 0.409662 | 0.686807 |
| FDXR | 0.168346 | 5.364914 | 0.408396 | 0.68772 |
| SORBS1 | 0.147546 | 8.504451 | 0.408105 | 0.68793 |
| GABRA3 | -0.23084 | 1.468742 | -0.40722 | 0.688571 |
| NRXN2 | 0.349509 | 2.763927 | 0.40426 | 0.690705 |
| REG1A | 0.256273 | 2.979746 | 0.403577 | 0.691199 |
| BAK1 | 0.118237 | 7.008097 | 0.403177 | 0.691487 |
| GPNMB | -0.16008 | 11.30639 | -0.40317 | 0.691496 |
| BCL2L10 | 0.294246 | 3.199503 | 0.402641 | 0.691875 |
| FABP5 | 0.27962 | 12.0378 | 0.40248 | 0.691991 |
| CALR | 0.125635 | 9.919994 | 0.4016 | 0.692628 |
| COL5A2 | 0.231793 | 10.55836 | 0.400287 | 0.693578 |
| RAD1 | 0.131638 | 7.379419 | 0.400038 | 0.693757 |
| PLAU | 0.19478 | 9.707016 | 0.399804 | 0.693927 |
| RPL39 | 0.112096 | 12.66605 | 0.399114 | 0.694426 |
| CDKN2D | 0.13511 | 5.518192 | 0.398339 | 0.694987 |
| BMP6 | 0.135227 | 6.199211 | 0.396915 | 0.696019 |
| STK25 | 0.139822 | 7.012644 | 0.396307 | 0.69646 |
| IGSF6 | 0.171613 | 5.845023 | 0.395799 | 0.696828 |
| POLD1 | -0.13404 | 6.447485 | -0.39524 | 0.697232 |
| CCR1 | 0.19765 | 7.068853 | 0.395154 | 0.697296 |
| SERPINA5 | -0.17063 | 3.83462 | -0.39504 | 0.697376 |
| RPA2 | -0.10473 | 8.42116 | -0.39325 | 0.698679 |
| DPYD | -0.1828 | 7.233114 | -0.3926 | 0.699149 |
| DARS | -0.15272 | 9.837317 | -0.39134 | 0.700061 |
| STAT2 | 0.118968 | 6.298394 | 0.389597 | 0.701329 |
| USH1C | -0.1879 | 5.388452 | -0.38915 | 0.701652 |
| LAMC2 | 0.329302 | 9.422139 | 0.389087 | 0.7017 |
| UPF1 | 0.105407 | 7.531424 | 0.388966 | 0.701788 |
| MAPK11 | -0.15061 | 6.240867 | -0.38862 | 0.702043 |
| SPRY2 | -0.24709 | 6.505806 | -0.38578 | 0.704108 |
| SLC27A5 | 0.185455 | 4.125418 | 0.385697 | 0.704166 |
| HES1 | -0.13232 | 7.807395 | -0.38473 | 0.704874 |
| TNFRSF21 | -0.20449 | 9.283954 | -0.38352 | 0.70575 |
| F13A1 | -0.2842 | 8.759135 | -0.38042 | 0.70801 |
| CYLD | -0.31265 | 6.64226 | -0.37846 | 0.709441 |
| ABCA1 | 0.141385 | 8.18842 | 0.378244 | 0.7096 |
| LIG3 | 0.140118 | 5.694208 | 0.377891 | 0.709857 |
| PTGES | 0.11993 | 7.034589 | 0.377734 | 0.709972 |
| JAK2 | -0.1397 | 5.998714 | -0.37751 | 0.710138 |
| PRKCG | 0.144725 | 1.630326 | 0.375676 | 0.711475 |
| ABAT | -0.15666 | 5.295666 | -0.37464 | 0.712231 |
| GAPDHS | -0.20855 | 5.205204 | -0.37258 | 0.713736 |
| EBI3 | 0.203195 | 2.497168 | 0.371724 | 0.714365 |
| HSPA5 | 0.136133 | 10.70791 | 0.371101 | 0.714822 |
| NXT1 | 0.107995 | 7.758988 | 0.369985 | 0.715639 |
| TGIF2 | 0.131009 | 6.635206 | 0.369881 | 0.715715 |
| CXCL10 | -0.29952 | 8.645392 | -0.36948 | 0.71601 |
| PPFIA4 | -0.26368 | 2.662721 | -0.36845 | 0.716766 |
| GNMT | -0.28664 | 3.056959 | -0.368 | 0.717093 |
| KLF4 | 0.217003 | 9.838164 | 0.366915 | 0.717889 |
| IGF1R | 0.17095 | 7.88765 | 0.365843 | 0.718675 |
| IL2RG | 0.119905 | 7.56096 | 0.365392 | 0.719006 |
| ITGB5 | -0.13564 | 8.536754 | -0.36313 | 0.720667 |
| RAD51C | 0.211023 | 6.216472 | 0.362133 | 0.721399 |
| MAPK13 | -0.15367 | 7.650432 | -0.36183 | 0.721618 |
| SELENBP1 | 0.189844 | 6.18696 | 0.359524 | 0.723317 |
| CX3CL1 | -0.257 | 5.933706 | -0.35914 | 0.723596 |
| ADM | -0.23863 | 9.206695 | -0.35781 | 0.724581 |
| CLDN4 | -0.29535 | 5.962535 | -0.35608 | 0.725852 |
| NAP1L1 | 0.093821 | 10.02235 | 0.356028 | 0.725889 |
| CD69 | -0.19057 | 6.488211 | -0.35592 | 0.725966 |
| CDKN2C | -0.19263 | 6.551104 | -0.3555 | 0.726279 |
| IDH2 | -0.16934 | 8.746103 | -0.35517 | 0.726524 |
| MPPED2 | 0.379963 | 3.168667 | 0.355139 | 0.726544 |
| BUB1 | -0.15543 | 6.545597 | -0.35352 | 0.72774 |
| JUP | -0.21956 | 10.41623 | -0.35351 | 0.727741 |
| PRIM2 | -0.18868 | 4.750251 | -0.35138 | 0.729313 |
| DEPTOR | 0.189297 | 6.481985 | 0.35026 | 0.730141 |
| TRO | 0.139103 | 5.805607 | 0.34989 | 0.730414 |
| GNG11 | -0.21991 | 7.159299 | -0.3493 | 0.730846 |
| ATOX1 | 0.112316 | 8.175053 | 0.347532 | 0.732155 |
| UBR7 | -0.08838 | 7.781631 | -0.34733 | 0.732304 |
| DAP3 | -0.10698 | 8.152295 | -0.34716 | 0.732431 |
| AVPR1A | 0.143054 | 4.259579 | 0.346451 | 0.732954 |
| ICOSLG | 0.13204 | 6.731123 | 0.34613 | 0.733191 |
| GPRC5B | 0.26825 | 6.278845 | 0.345788 | 0.733444 |
| CLIC3 | 0.330426 | 6.211064 | 0.345765 | 0.733461 |
| NLRP3 | -0.28483 | 3.617757 | -0.34572 | 0.733496 |
| ZNF185 | -0.16162 | 8.100593 | -0.34499 | 0.734036 |
| ABCD3 | -0.12087 | 3.589547 | -0.3441 | 0.73469 |
| HMGA1 | 0.18521 | 8.909587 | 0.343468 | 0.735159 |
| CACNG1 | -0.35154 | 4.631167 | -0.34317 | 0.735376 |
| RETSAT | -0.08944 | 7.009139 | -0.34284 | 0.735624 |
| SLC37A1 | -0.21769 | 2.602907 | -0.34252 | 0.735863 |
| PGLYRP1 | -0.17277 | 2.479674 | -0.33986 | 0.737828 |
| ZAP70 | 0.339237 | 3.583866 | 0.339828 | 0.737853 |
| ARFGAP3 | -0.08888 | 8.076771 | -0.33844 | 0.738882 |
| STRN | 0.179111 | 5.890224 | 0.336683 | 0.740185 |
| BTG2 | 0.104171 | 8.33295 | 0.336337 | 0.740441 |
| HLA-A | 0.084595 | 12.66028 | 0.335718 | 0.7409 |
| PIM1 | 0.123847 | 8.189951 | 0.335474 | 0.741081 |
| HGF | -0.13385 | 6.355315 | -0.33514 | 0.741332 |
| MTF2 | 0.086234 | 7.652803 | 0.334864 | 0.741534 |
| PKP3 | 0.138381 | 8.797427 | 0.333279 | 0.74271 |
| SPON1 | 0.236019 | 8.351015 | 0.332106 | 0.743581 |
| PCK1 | 0.395554 | 2.408434 | 0.331492 | 0.744037 |
| AK2 | 0.104834 | 7.995377 | 0.330716 | 0.744614 |
| TAP2 | 0.117686 | 6.794771 | 0.329494 | 0.745522 |
| DCTPP1 | -0.25161 | 7.696862 | -0.32873 | 0.746091 |
| CRYAB | -0.24073 | 9.210321 | -0.32828 | 0.746428 |
| SHROOM2 | 0.116779 | 6.938102 | 0.328103 | 0.746557 |
| COL4A2 | 0.187073 | 10.35318 | 0.325593 | 0.748424 |
| KRT1 | -0.40008 | 8.047894 | -0.32514 | 0.748763 |
| NADSYN1 | 0.256131 | 5.780281 | 0.325082 | 0.748805 |
| PTTG1 | 0.113674 | 9.140652 | 0.324265 | 0.749413 |
| STX12 | 0.069049 | 7.990176 | 0.323506 | 0.749978 |
| PTGER3 | 0.12128 | 6.043202 | 0.322008 | 0.751094 |
| HUS1 | -0.28026 | 6.300457 | -0.32137 | 0.751572 |
| PRDX1 | -0.11083 | 11.25943 | -0.3211 | 0.751771 |
| EPB41L2 | 0.137778 | 7.518258 | 0.320747 | 0.752034 |
| TPSAB1 | -0.21343 | 7.809363 | -0.31954 | 0.752935 |
| GSTK1 | -0.11596 | 8.503179 | -0.31926 | 0.753141 |
| MAPK12 | 0.132368 | 5.756118 | 0.318372 | 0.753806 |
| TIMELESS | -0.12574 | 6.86405 | -0.31752 | 0.75444 |
| MAPRE3 | 0.262507 | 4.17387 | 0.317174 | 0.7547 |
| LSR | -0.15541 | 7.918458 | -0.317 | 0.754829 |
| IGFBP4 | -0.24772 | 9.769933 | -0.31686 | 0.754931 |
| CLN5 | 0.094102 | 7.006798 | 0.315962 | 0.755605 |
| CLDN11 | 0.231736 | 4.928357 | 0.315254 | 0.756133 |
| PPP1R8 | -0.0693 | 7.896165 | -0.31474 | 0.756518 |
| CCL4 | 0.139172 | 7.375294 | 0.314042 | 0.757039 |
| ELF3 | -0.16427 | 7.443172 | -0.31299 | 0.757824 |
| LCP2 | 0.24052 | 6.403814 | 0.311129 | 0.759217 |
| OCEL1 | -0.07634 | 6.065861 | -0.30964 | 0.760328 |
| BIRC5 | 0.110099 | 7.092826 | 0.307666 | 0.761809 |
| CELSR2 | 0.145252 | 8.113959 | 0.307006 | 0.762303 |
| TLR3 | -0.16527 | 5.550068 | -0.30689 | 0.762389 |
| MRPL3 | -0.10809 | 9.553821 | -0.30615 | 0.762945 |
| GAMT | -0.3402 | 3.707959 | -0.30538 | 0.763525 |
| SLC6A6 | 0.217226 | 3.349749 | 0.304715 | 0.76402 |
| CCNF | -0.11754 | 5.738604 | -0.30391 | 0.764623 |
| MKNK1 | -0.07042 | 8.398865 | -0.29947 | 0.767956 |
| RQCD1 | -0.06136 | 7.537315 | -0.29869 | 0.768542 |
| TGFB3 | 0.150065 | 7.648123 | 0.298151 | 0.768945 |
| SGCA | -0.24735 | 2.040052 | -0.29564 | 0.770834 |
| SQLE | 0.166739 | 9.019889 | 0.295 | 0.771313 |
| HMMR | 0.184725 | 6.615208 | 0.294317 | 0.771826 |
| SCAMP1 | -0.09285 | 8.53599 | -0.2925 | 0.773194 |
| VCAM1 | -0.16894 | 7.449257 | -0.29178 | 0.773738 |
| GALC | 0.09174 | 7.311537 | 0.290671 | 0.77457 |
| MCM7 | -0.09449 | 7.892531 | -0.29049 | 0.774704 |
| BUB3 | -0.10024 | 8.65287 | -0.29 | 0.775074 |
| EPB41L3 | -0.1757 | 6.032783 | -0.28976 | 0.775256 |
| SLC7A5 | -0.19073 | 9.594839 | -0.28872 | 0.776038 |
| NEDD9 | 0.148855 | 6.820448 | 0.28564 | 0.778361 |
| HMGB3 | -0.09614 | 7.946214 | -0.28549 | 0.778476 |
| CASP8 | 0.086407 | 6.803918 | 0.284193 | 0.779452 |
| KLF7 | 0.100919 | 8.10676 | 0.283703 | 0.779821 |
| ALDH1A1 | 0.334446 | 5.601532 | 0.283691 | 0.779831 |
| ABCA2 | 0.075324 | 6.259285 | 0.283138 | 0.780247 |
| RARS | 0.074834 | 8.165445 | 0.282504 | 0.780726 |
| ABCD2 | 0.125488 | 3.754662 | 0.281793 | 0.781263 |
| IL7 | 0.209572 | 3.965825 | 0.280349 | 0.782353 |
| MMP9 | 0.184239 | 9.738967 | 0.279589 | 0.782927 |
| MKI67 | 0.121209 | 6.950412 | 0.279581 | 0.782933 |
| PFKFB3 | -0.11841 | 8.088043 | -0.27746 | 0.784535 |
| CLU | -0.39136 | 6.768577 | -0.27703 | 0.784863 |
| TXNRD2 | 0.147722 | 5.051774 | 0.276147 | 0.785528 |
| ABCE1 | -0.10652 | 7.964116 | -0.27581 | 0.785781 |
| RXRA | 0.096639 | 8.02776 | 0.275414 | 0.786082 |
| ATP6V1B1 | 0.241662 | 3.20347 | 0.275315 | 0.786158 |
| CXCL9 | -0.19129 | 7.891011 | -0.27272 | 0.788117 |
| PRLR | -0.13407 | 3.733965 | -0.27262 | 0.788193 |
| PLAC1 | -0.19429 | 6.153078 | -0.2726 | 0.788211 |
| LGALS3 | -0.0684 | 10.64991 | -0.27166 | 0.788923 |
| RBL1 | -0.08651 | 4.722552 | -0.27033 | 0.789932 |
| CXCL13 | -0.23949 | 7.361805 | -0.26985 | 0.790293 |
| IGFBP7 | 0.141347 | 11.07307 | 0.269336 | 0.790683 |
| THSD4 | -0.10907 | 5.399645 | -0.26931 | 0.790702 |
| LALBA | -0.20231 | 3.69309 | -0.26912 | 0.790844 |
| ST8SIA4 | -0.08133 | 5.019098 | -0.26894 | 0.79098 |
| PRPS1 | 0.065073 | 8.058281 | 0.268763 | 0.791118 |
| POLD3 | -0.07453 | 6.716993 | -0.26866 | 0.791198 |
| PGF | 0.108033 | 8.953458 | 0.268571 | 0.791263 |
| SRGN | 0.130172 | 9.763126 | 0.268423 | 0.791375 |
| SHMT1 | 0.077141 | 5.068049 | 0.267889 | 0.791779 |
| RAB2A | -0.07935 | 9.120851 | -0.26475 | 0.794159 |
| TJP1 | -0.06761 | 9.0808 | -0.26406 | 0.794686 |
| PTP4A3 | 0.156948 | 6.379963 | 0.262509 | 0.795861 |
| GBP2 | -0.12684 | 7.796365 | -0.26212 | 0.796157 |
| ILVBL | 0.076492 | 7.670919 | 0.260926 | 0.797062 |
| PDCD1LG2 | 0.120157 | 2.093775 | 0.26089 | 0.797089 |
| ENO2 | -0.30225 | 5.070012 | -0.26089 | 0.797093 |
| FES | 0.19129 | 2.9968 | 0.260762 | 0.797187 |
| IL27RA | -0.09471 | 6.714109 | -0.2603 | 0.797537 |
| SULT2B1 | -0.1635 | 6.890408 | -0.25904 | 0.798494 |
| CSF1 | -0.10217 | 6.949016 | -0.25857 | 0.798854 |
| PAPD7 | 0.068869 | 8.376397 | 0.258447 | 0.798945 |
| TH | 0.220555 | 3.473741 | 0.258184 | 0.799145 |
| NRP1 | -0.09987 | 7.523473 | -0.25756 | 0.799621 |
| DDIT3 | -0.07176 | 7.254817 | -0.25728 | 0.799831 |
| VWA5A | -0.09086 | 7.01073 | -0.25583 | 0.800938 |
| CNN3 | -0.13536 | 7.958772 | -0.2557 | 0.801031 |
| GSR | -0.20707 | 5.439546 | -0.25544 | 0.801228 |
| ATP6AP1 | -0.06679 | 9.278033 | -0.25419 | 0.802184 |
| BDKRB2 | 0.063035 | 6.886302 | 0.252576 | 0.80341 |
| BCAN | 0.088929 | 7.260647 | 0.250828 | 0.80474 |
| GPX3 | 0.214413 | 8.717434 | 0.250798 | 0.804764 |
| TST | 0.245254 | 6.765802 | 0.249479 | 0.805768 |
| TRIP13 | 0.104515 | 7.813035 | 0.248856 | 0.806243 |
| CXCR4 | 0.148156 | 8.713596 | 0.248777 | 0.806303 |
| PDGFRB | -0.12103 | 8.105141 | -0.24852 | 0.806501 |
| RABEP1 | -0.0651 | 5.971438 | -0.24495 | 0.809218 |
| PLCG1 | -0.1018 | 6.457131 | -0.24475 | 0.809372 |
| MYD88 | -0.0695 | 9.276505 | -0.24266 | 0.810965 |
| IL2RA | 0.086557 | 6.244558 | 0.242361 | 0.811195 |
| CA12 | 0.137654 | 8.986891 | 0.240373 | 0.812712 |
| PRC1 | -0.10224 | 7.759177 | -0.23936 | 0.813482 |
| RNASEL | 0.111277 | 5.490005 | 0.239313 | 0.813522 |
| MYL7 | -0.14629 | 2.956254 | -0.239 | 0.813759 |
| PTCD2 | -0.11649 | 3.465987 | -0.23844 | 0.814188 |
| KDM4B | -0.13938 | 6.260497 | -0.23814 | 0.814419 |
| NDUFB4 | 0.08551 | 10.32261 | 0.237635 | 0.814804 |
| CHPT1 | 0.07575 | 7.803356 | 0.23683 | 0.815418 |
| COL1A1 | 0.145475 | 12.35163 | 0.236457 | 0.815703 |
| METTL3 | -0.06581 | 7.60956 | -0.23641 | 0.815742 |
| ST5 | 0.09197 | 7.848914 | 0.234033 | 0.817557 |
| TSKU | -0.07641 | 7.723655 | -0.23266 | 0.818609 |
| HKDC1 | -0.19796 | 3.243134 | -0.23202 | 0.819098 |
| MAP2K6 | 0.05815 | 6.234485 | 0.231946 | 0.819152 |
| ALDH1A2 | -0.1131 | 3.6427 | -0.23176 | 0.819294 |
| IFNAR1 | -0.08434 | 5.384009 | -0.23144 | 0.819538 |
| OVOL2 | 0.082767 | 6.678921 | 0.231115 | 0.819789 |
| ADAMTS5 | -0.08416 | 6.121817 | -0.23069 | 0.820111 |
| AP3S1 | -0.05226 | 9.832258 | -0.23029 | 0.820417 |
| UNC13B | -0.06814 | 7.841394 | -0.23029 | 0.820417 |
| PERP | -0.12414 | 11.29773 | -0.22982 | 0.820783 |
| ARL3 | -0.06199 | 7.20129 | -0.22965 | 0.820912 |
| COL9A1 | 0.113323 | 2.244872 | 0.229279 | 0.821194 |
| XRCC3 | 0.140651 | 4.056286 | 0.22795 | 0.822211 |
| SLC6A8 | -0.10979 | 7.714946 | -0.227 | 0.822939 |
| ARFGEF1 | -0.08379 | 7.56538 | -0.22698 | 0.822952 |
| SLC27A2 | 0.174522 | 3.022219 | 0.226152 | 0.823589 |
| NR3C1 | -0.05916 | 9.068821 | -0.226 | 0.823707 |
| SEC31A | 0.05131 | 9.977866 | 0.223553 | 0.82558 |
| GRB7 | 0.205522 | 7.081456 | 0.223494 | 0.825626 |
| ATP7A | 0.072731 | 5.765174 | 0.221742 | 0.826969 |
| AARS | -0.05506 | 9.0226 | -0.21927 | 0.828862 |
| CSF2 | -0.1955 | 3.875316 | -0.21927 | 0.828864 |
| NBN | -0.07478 | 8.23942 | -0.21844 | 0.829503 |
| GC | 0.191709 | 4.325899 | 0.217977 | 0.829858 |
| IRS1 | 0.088664 | 7.334781 | 0.216566 | 0.830941 |
| HDAC9 | -0.07762 | 5.811016 | -0.21529 | 0.831924 |
| HELLS | -0.24097 | 3.932642 | -0.21411 | 0.83283 |
| SQSTM1 | -0.07653 | 9.372012 | -0.21296 | 0.83371 |
| SLC16A1 | 0.091049 | 8.511518 | 0.212633 | 0.833963 |
| VIPR1 | -0.11111 | 6.290984 | -0.2115 | 0.834834 |
| AEBP1 | -0.16956 | 8.661926 | -0.20927 | 0.836548 |
| MYB | -0.1234 | 5.590938 | -0.20893 | 0.836809 |
| POP7 | 0.069296 | 7.711218 | 0.208008 | 0.837519 |
| SYBU | -0.08881 | 6.624491 | -0.20782 | 0.837664 |
| GZMA | 0.142882 | 6.198527 | 0.206744 | 0.838491 |
| INPPL1 | -0.07501 | 7.228514 | -0.20634 | 0.838802 |
| THBD | 0.12499 | 8.843175 | 0.20622 | 0.838895 |
| EIF5A | 0.196453 | 8.43158 | 0.20617 | 0.838933 |
| PECR | 0.109255 | 3.215676 | 0.205719 | 0.83928 |
| PTEN | -0.05312 | 8.352902 | -0.20443 | 0.840276 |
| PFKP | -0.08315 | 8.686012 | -0.204 | 0.840606 |
| FAM162A | 0.077974 | 9.264983 | 0.201434 | 0.84258 |
| PEX26 | -0.05591 | 6.346118 | -0.20069 | 0.843155 |
| IRF4 | -0.06208 | 6.644365 | -0.19985 | 0.843798 |
| HK1 | 0.054662 | 8.998478 | 0.199823 | 0.843822 |
| MMD | 0.067556 | 7.783543 | 0.199543 | 0.844038 |
| AP3B1 | 0.053183 | 7.916636 | 0.197888 | 0.845314 |
| MLH1 | -0.04919 | 8.199831 | -0.19724 | 0.845814 |
| MCM3 | -0.06339 | 8.232666 | -0.19646 | 0.846416 |
| HOXD11 | 0.134791 | 5.590195 | 0.194687 | 0.847782 |
| MAPT | 0.137221 | 5.230876 | 0.194394 | 0.848008 |
| RAB17 | 0.088291 | 5.624836 | 0.194327 | 0.84806 |
| SYNCRIP | 0.078849 | 8.618085 | 0.192551 | 0.84943 |
| LAD1 | -0.11626 | 9.025837 | -0.19243 | 0.849521 |
| VCL | -0.0621 | 10.20566 | -0.19091 | 0.8507 |
| SYNGR2 | -0.05042 | 9.275673 | -0.19082 | 0.85077 |
| MADCAM1 | 0.071816 | 5.944802 | 0.190659 | 0.850891 |
| PPP3R1 | -0.07352 | 6.539258 | -0.1899 | 0.85148 |
| MYCN | -0.18308 | 3.722823 | -0.18927 | 0.851966 |
| NCL | 0.051351 | 10.66931 | 0.188982 | 0.852186 |
| SULT1B1 | -0.0751 | 5.982442 | -0.18791 | 0.853012 |
| PDGFC | -0.08227 | 8.949266 | -0.18725 | 0.853524 |
| DOPEY1 | -0.06404 | 5.700589 | -0.18723 | 0.853542 |
| DCLRE1B | -0.06349 | 5.389884 | -0.18715 | 0.8536 |
| POLE | -0.07223 | 6.398141 | -0.18634 | 0.854226 |
| E2F3 | 0.052418 | 6.868375 | 0.18556 | 0.85483 |
| ADRBK1 | -0.05128 | 8.252922 | -0.18541 | 0.854947 |
| CYR61 | 0.093304 | 9.713048 | 0.184756 | 0.855452 |
| VAV3 | -0.10562 | 7.8069 | -0.18333 | 0.856551 |
| CDC25B | -0.04589 | 8.841653 | -0.18324 | 0.856624 |
| PAM | 0.087125 | 9.507875 | 0.182167 | 0.857453 |
| SPAG5 | -0.04798 | 5.989836 | -0.18087 | 0.858458 |
| LXN | 0.104775 | 7.290217 | 0.180461 | 0.858773 |
| DNMT1 | -0.05109 | 8.321629 | -0.17908 | 0.859843 |
| CAT | -0.06157 | 9.019238 | -0.17853 | 0.860265 |
| WSB1 | 0.062248 | 8.977856 | 0.178113 | 0.86059 |
| PRSS23 | 0.08962 | 8.875865 | 0.17799 | 0.860685 |
| JAG1 | -0.05705 | 10.90951 | -0.17415 | 0.863656 |
| TPST2 | -0.04522 | 7.937472 | -0.17377 | 0.863955 |
| PSMB8 | 0.06904 | 8.088918 | 0.173514 | 0.864152 |
| SMC1A | -0.05054 | 8.284941 | -0.17295 | 0.864585 |
| TNFAIP3 | 0.08294 | 9.652042 | 0.171938 | 0.865373 |
| LAMP2 | 0.060226 | 9.114075 | 0.169481 | 0.867277 |
| CDH11 | -0.11953 | 8.871181 | -0.16589 | 0.870064 |
| SNX2 | 0.046099 | 7.559819 | 0.165368 | 0.870466 |
| PDK3 | 0.047665 | 7.087706 | 0.165051 | 0.870713 |
| SPC24 | 0.08984 | 3.354482 | 0.164412 | 0.871209 |
| APOH | 0.144256 | 3.529965 | 0.163342 | 0.872039 |
| CD80 | -0.05262 | 6.705792 | -0.16238 | 0.872785 |
| OPN3 | 0.087625 | 8.12975 | 0.161988 | 0.873089 |
| B2M | -0.03986 | 12.80928 | -0.16189 | 0.873165 |
| LDHA | 0.040143 | 12.16091 | 0.161287 | 0.873634 |
| ZAK | -0.0694 | 5.614581 | -0.16081 | 0.874007 |
| LAMA2 | -0.08586 | 6.585547 | -0.16029 | 0.87441 |
| IL11 | -0.09779 | 5.559302 | -0.15995 | 0.874675 |
| H2AFV | 0.049652 | 9.246498 | 0.159908 | 0.874705 |
| HNRNPD | 0.045292 | 9.586766 | 0.159216 | 0.875242 |
| MAPK9 | 0.043414 | 7.13844 | 0.158885 | 0.875499 |
| PAOX | -0.04668 | 5.759051 | -0.15751 | 0.876567 |
| CENPF | -0.07295 | 7.303128 | -0.15635 | 0.877472 |
| NLGN3 | -0.09325 | 3.485922 | -0.15584 | 0.877867 |
| DAP | 0.055803 | 7.699629 | 0.154824 | 0.878654 |
| NUP153 | 0.038255 | 8.408232 | 0.15212 | 0.880756 |
| TLR1 | -0.05583 | 5.443523 | -0.15039 | 0.8821 |
| SOAT2 | -0.09927 | 3.82397 | -0.14971 | 0.882633 |
| FKBP4 | -0.05668 | 7.702296 | -0.14941 | 0.882863 |
| CHST8 | 0.049736 | 5.051417 | 0.149299 | 0.88295 |
| ERO1L | 0.090129 | 6.371673 | 0.148931 | 0.883236 |
| CLEC4A | -0.10898 | 5.348215 | -0.14778 | 0.884131 |
| GINS4 | 0.095009 | 5.092298 | 0.14753 | 0.884326 |
| HIST1H2BK | 0.059807 | 9.276133 | 0.146423 | 0.885188 |
| CKS1B | -0.06303 | 8.586611 | -0.1464 | 0.885205 |
| GPC1 | -0.06703 | 7.981318 | -0.14563 | 0.885805 |
| NR1I2 | -0.10075 | 4.967169 | -0.14388 | 0.887166 |
| CD34 | 0.057502 | 7.088958 | 0.143251 | 0.887657 |
| CD9 | -0.06688 | 10.70953 | -0.1418 | 0.888784 |
| MAP7 | -0.06528 | 6.729832 | -0.13921 | 0.890806 |
| SCG3 | -0.1036 | 0.380641 | -0.13904 | 0.890937 |
| TPD52L1 | -0.0505 | 9.411239 | -0.13773 | 0.891956 |
| ASCL1 | -0.09525 | 4.660408 | -0.13511 | 0.893997 |
| CYP46A1 | -0.11325 | 4.41155 | -0.13397 | 0.894885 |
| CHMP1A | -0.03705 | 7.517115 | -0.13371 | 0.895093 |
| YRDC | -0.03531 | 7.223518 | -0.13335 | 0.895372 |
| RPA3 | -0.04338 | 8.167507 | -0.1324 | 0.89611 |
| MAST4 | -0.06253 | 7.867074 | -0.13039 | 0.89768 |
| ETV4 | 0.090839 | 4.948153 | 0.128163 | 0.899417 |
| GNA15 | 0.054287 | 8.086041 | 0.127681 | 0.899794 |
| FANCC | 0.028812 | 6.849457 | 0.125004 | 0.901883 |
| TAPBP | -0.04015 | 9.358441 | -0.12482 | 0.902029 |
| MYBPC3 | -0.05841 | 4.954873 | -0.12354 | 0.903028 |
| USP12 | -0.03024 | 6.693067 | -0.12335 | 0.903171 |
| CYP39A1 | -0.16612 | 3.279142 | -0.12326 | 0.903242 |
| FBXO4 | -0.08982 | 3.35764 | -0.12321 | 0.90328 |
| PDK1 | 0.108219 | 4.626142 | 0.123056 | 0.903403 |
| BLVRB | -0.03823 | 7.11032 | -0.12147 | 0.904646 |
| PLA2G12A | -0.038 | 5.816896 | -0.1211 | 0.904929 |
| PTGS2 | -0.11441 | 6.400169 | -0.12033 | 0.905529 |
| TFRC | 0.055417 | 10.48425 | 0.118126 | 0.907254 |
| CITED2 | 0.044745 | 6.858036 | 0.117232 | 0.907953 |
| CDK4 | 0.03928 | 8.757939 | 0.116755 | 0.908325 |
| SLA | 0.040937 | 7.860566 | 0.116645 | 0.908411 |
| CCNE1 | -0.03721 | 5.979738 | -0.11646 | 0.908555 |
| ALOX15B | -0.06366 | 5.095691 | -0.11612 | 0.908822 |
| RELN | -0.0443 | 5.67164 | -0.11606 | 0.908865 |
| FGFR3 | -0.12023 | 7.416473 | -0.11595 | 0.908954 |
| ASS1 | 0.063669 | 8.454355 | 0.115187 | 0.909551 |
| PAFAH1B1 | -0.03834 | 8.194849 | -0.11483 | 0.909833 |
| PMAIP1 | -0.07813 | 7.729119 | -0.11471 | 0.909921 |
| PTRF | -0.03703 | 9.567378 | -0.11403 | 0.910452 |
| ACSL5 | 0.032756 | 6.360478 | 0.111774 | 0.912219 |
| PBX2 | 0.050416 | 6.617552 | 0.11055 | 0.913176 |
| AMFR | 0.048263 | 6.309867 | 0.105986 | 0.916746 |
| TLR6 | 0.03751 | 5.196343 | 0.105855 | 0.916848 |
| EREG | 0.108256 | 5.582599 | 0.105787 | 0.916902 |
| FAS | 0.049648 | 7.156791 | 0.105453 | 0.917163 |
| HSD17B11 | -0.03426 | 7.404644 | -0.10535 | 0.917245 |
| PLK4 | 0.056735 | 5.949012 | 0.105333 | 0.917257 |
| VDAC2 | 0.02993 | 10.52123 | 0.105138 | 0.917409 |
| PLXNB2 | 0.041024 | 8.950348 | 0.103149 | 0.918966 |
| GREB1 | 0.057862 | 4.025534 | 0.102641 | 0.919363 |
| MAPK1 | 0.022559 | 8.273696 | 0.102523 | 0.919456 |
| PRKCDBP | 0.039976 | 7.551642 | 0.101023 | 0.920631 |
| CD47 | -0.04042 | 9.898571 | -0.10051 | 0.921031 |
| MYH10 | -0.02818 | 8.472356 | -0.09995 | 0.921474 |
| E2F4 | 0.029804 | 9.196951 | 0.098215 | 0.922829 |
| IRF1 | -0.03149 | 7.995585 | -0.09767 | 0.923256 |
| RET | 0.039527 | 6.759579 | 0.09707 | 0.923726 |
| GPC4 | 0.062517 | 6.296576 | 0.096952 | 0.923818 |
| RAF1 | 0.023488 | 8.21027 | 0.096625 | 0.924074 |
| PLA2G16 | 0.102594 | 4.772404 | 0.095306 | 0.925107 |
| LYN | 0.037562 | 7.690373 | 0.094096 | 0.926055 |
| DLGAP5 | -0.05942 | 6.845783 | -0.09371 | 0.926354 |
| ABCC1 | 0.039063 | 7.074913 | 0.09276 | 0.927102 |
| CISH | -0.0264 | 7.872677 | -0.09208 | 0.927633 |
| KDELR3 | -0.04222 | 8.052122 | -0.09206 | 0.927652 |
| GJB3 | -0.04936 | 8.365165 | -0.09131 | 0.928235 |
| CNTN1 | 0.040124 | 6.258666 | 0.091097 | 0.928405 |
| NRIP1 | -0.03736 | 8.916803 | -0.08927 | 0.929839 |
| ITGB1 | -0.02479 | 11.15662 | -0.08615 | 0.932283 |
| TIAL1 | 0.02129 | 8.637869 | 0.086108 | 0.932315 |
| BCL2 | 0.038338 | 6.178618 | 0.085274 | 0.932969 |
| CFHR2 | -0.02756 | 6.255345 | -0.08481 | 0.933332 |
| IKBKG | -0.01866 | 7.960821 | -0.08294 | 0.934796 |
| LSP1 | 0.057215 | 6.822863 | 0.081343 | 0.936052 |
| DYRK3 | 0.03182 | 5.887028 | 0.080762 | 0.936507 |
| ARCN1 | 0.024072 | 8.352173 | 0.080146 | 0.936991 |
| NDUFA6 | -0.0298 | 9.019176 | -0.0792 | 0.937733 |
| AFF1 | -0.02344 | 8.865782 | -0.07888 | 0.937986 |
| GLA | -0.02395 | 7.954819 | -0.07877 | 0.938074 |
| TUBG1 | -0.02483 | 7.408323 | -0.07725 | 0.939263 |
| TROAP | 0.023852 | 6.780306 | 0.07637 | 0.939953 |
| PGK1 | -0.02216 | 11.08469 | -0.07531 | 0.940787 |
| GINS3 | 0.03276 | 6.29115 | 0.074501 | 0.94142 |
| GOSR2 | 0.021156 | 6.909232 | 0.074321 | 0.941561 |
| MXD3 | -0.02227 | 3.657067 | -0.07338 | 0.942302 |
| BIK | 0.073789 | 5.601748 | 0.072804 | 0.942751 |
| KIF5A | 0.026887 | 5.977437 | 0.072743 | 0.942799 |
| ESPL1 | -0.02793 | 7.056413 | -0.07075 | 0.944366 |
| SLC26A2 | -0.04271 | 6.798249 | -0.07013 | 0.944847 |
| ANKH | -0.05637 | 4.677194 | -0.06947 | 0.945372 |
| COL3A1 | 0.028305 | 12.52468 | 0.063872 | 0.949764 |
| INPP5F | -0.02187 | 6.87206 | -0.06283 | 0.950586 |
| MOCS2 | 0.021269 | 6.044349 | 0.060614 | 0.952323 |
| EXT1 | 0.022352 | 8.33866 | 0.059846 | 0.952926 |
| RBPJ | 0.014203 | 9.164703 | 0.059735 | 0.953014 |
| CD99 | 0.022993 | 10.36616 | 0.056847 | 0.955283 |
| MAFF | 0.026219 | 8.093954 | 0.05564 | 0.956231 |
| CANT1 | -0.01674 | 8.055152 | -0.05452 | 0.957111 |
| PIGR | 0.034221 | 6.644531 | 0.053636 | 0.957806 |
| WHSC1 | 0.050112 | 5.422548 | 0.053576 | 0.957853 |
| ACACA | 0.03648 | 7.262064 | 0.052116 | 0.959001 |
| PLEK2 | -0.02989 | 8.43198 | -0.05201 | 0.959085 |
| PDLIM1 | -0.01304 | 10.75376 | -0.05193 | 0.959146 |
| COX6C | -0.01637 | 11.04664 | -0.05135 | 0.959603 |
| GPR65 | -0.01643 | 5.662908 | -0.05101 | 0.959869 |
| ABCA4 | -0.03383 | 4.140079 | -0.05069 | 0.960121 |
| CCR5 | -0.03533 | 5.848963 | -0.05035 | 0.960388 |
| SORD | -0.02618 | 8.25529 | -0.0503 | 0.960432 |
| DUSP3 | -0.01999 | 7.674479 | -0.04839 | 0.961933 |
| PLAC8 | -0.02309 | 6.985869 | -0.04825 | 0.962036 |
| PAICS | 0.018699 | 8.407985 | 0.048156 | 0.962113 |
| ABCB1 | 0.014596 | 5.441733 | 0.047288 | 0.962795 |
| PRF1 | 0.018396 | 6.840822 | 0.046486 | 0.963426 |
| NUDT21 | -0.01857 | 7.514241 | -0.04641 | 0.963487 |
| AK1 | -0.02325 | 7.748678 | -0.04629 | 0.963578 |
| ABCD1 | -0.01285 | 6.753772 | -0.04571 | 0.964039 |
| ITGA10 | -0.01269 | 6.572893 | -0.04531 | 0.96435 |
| RPS6KA1 | 0.014458 | 7.108656 | 0.044465 | 0.965015 |
| CENPA | 0.012986 | 6.884265 | 0.043105 | 0.966084 |
| ECSIT | 0.027228 | 5.971347 | 0.040684 | 0.967988 |
| GAB2 | 0.028185 | 5.582648 | 0.040389 | 0.96822 |
| B4GALT1 | 0.018742 | 7.185443 | 0.04006 | 0.968479 |
| PBK | 0.019152 | 6.520908 | 0.039561 | 0.968872 |
| IKBKB | -0.01476 | 7.143678 | -0.03817 | 0.969964 |
| UNC119 | -0.01487 | 3.858196 | -0.03733 | 0.970629 |
| CELSR1 | 0.017828 | 6.58507 | 0.037221 | 0.970711 |
| SYK | -0.02352 | 5.677607 | -0.03699 | 0.970893 |
| PVRL1 | -0.01968 | 5.123992 | -0.03692 | 0.97095 |
| DFFA | -0.01169 | 7.429572 | -0.03675 | 0.971086 |
| COL6A2 | 0.028573 | 10.32431 | 0.036507 | 0.971273 |
| ACVR1B | 0.009188 | 7.890231 | 0.035264 | 0.972251 |
| SLC2A1 | 0.019689 | 9.197081 | 0.034593 | 0.972779 |
| MAPK10 | 0.012524 | 7.018377 | 0.034496 | 0.972855 |
| HR | -0.03458 | 4.786576 | -0.03418 | 0.973103 |
| ICAM4 | 0.015228 | 4.755153 | 0.031486 | 0.975223 |
| SNRPB | -0.00848 | 10.00133 | -0.02929 | 0.97695 |
| SPARC | 0.014943 | 11.50632 | 0.029118 | 0.977086 |
| HIF1A | -0.01021 | 10.94062 | -0.02901 | 0.977172 |
| TIPIN | -0.01805 | 5.403641 | -0.02879 | 0.977347 |
| POSTN | -0.01783 | 11.22767 | -0.02641 | 0.979216 |
| SLC24A3 | 0.022359 | 6.037057 | 0.025999 | 0.97954 |
| RRP12 | -0.01083 | 6.257729 | -0.0248 | 0.980484 |
| VPS45 | -0.00716 | 7.153828 | -0.02467 | 0.980584 |
| FGFR1 | -0.00892 | 7.235991 | -0.02175 | 0.982885 |
| H2AFZ | -0.0074 | 10.57169 | -0.02124 | 0.983286 |
| PRKCA | 0.006306 | 6.620906 | 0.021121 | 0.983377 |
| SHC1 | -0.00613 | 9.906309 | -0.01974 | 0.984461 |
| LY96 | -0.00795 | 7.526111 | -0.01782 | 0.985974 |
| IGF2R | 0.005296 | 8.530611 | 0.01767 | 0.986093 |
| SGCE | 0.008377 | 6.744705 | 0.017125 | 0.986522 |
| SEC22B | 0.006999 | 8.565684 | 0.016153 | 0.987287 |
| IFNGR2 | 0.004609 | 9.586568 | 0.015854 | 0.987522 |
| ELOVL2 | -0.0086 | 5.070699 | -0.01584 | 0.987537 |
| ARG1 | -0.00693 | 5.030285 | -0.01555 | 0.987765 |
| KRT18 | -0.0057 | 8.801928 | -0.01538 | 0.987899 |
| NPM1 | 0.003271 | 11.39196 | 0.015031 | 0.98817 |
| MUC1 | -0.00525 | 6.610585 | -0.01304 | 0.989735 |
| TP53 | 0.005781 | 7.417142 | 0.011817 | 0.9907 |
| ENDOD1 | -0.00364 | 8.420571 | -0.01181 | 0.990707 |
| SOCS1 | 0.006607 | 5.75861 | 0.0111 | 0.991264 |
| IFNG | -0.00296 | 5.574523 | -0.01099 | 0.991353 |
| EFNA5 | -0.00308 | 7.089224 | -0.01005 | 0.99209 |
| TFCP2L1 | -0.00455 | 6.915771 | -0.00989 | 0.992213 |
| NASP | 0.003581 | 7.710294 | 0.007956 | 0.993738 |
| IL2RB | -0.00206 | 7.558136 | -0.00688 | 0.994582 |
| PRKAR2A | -0.00143 | 8.35986 | -0.00373 | 0.997065 |
| CDKN2A | 0.003065 | 7.516459 | 0.003359 | 0.997357 |
| CP | 0.002631 | 5.056322 | 0.002861 | 0.997748 |
| PTPN1 | -0.00043 | 7.775832 | -0.00141 | 0.998889 |
| WASL | 0.000161 | 5.003029 | 0.000242 | 0.99981 |
