# Supplementary figures and images for: Expression profile analysis identifies IER3 to predict overall survival and promote lymph node metastasis in tongue cancer
Source: Cancer Cell Int. 2019 Nov 21;19:307. doi: 10.1186/s12935-019-1028-2 (PMC6873470; doi:10.1186/s12935-019-1028-2)

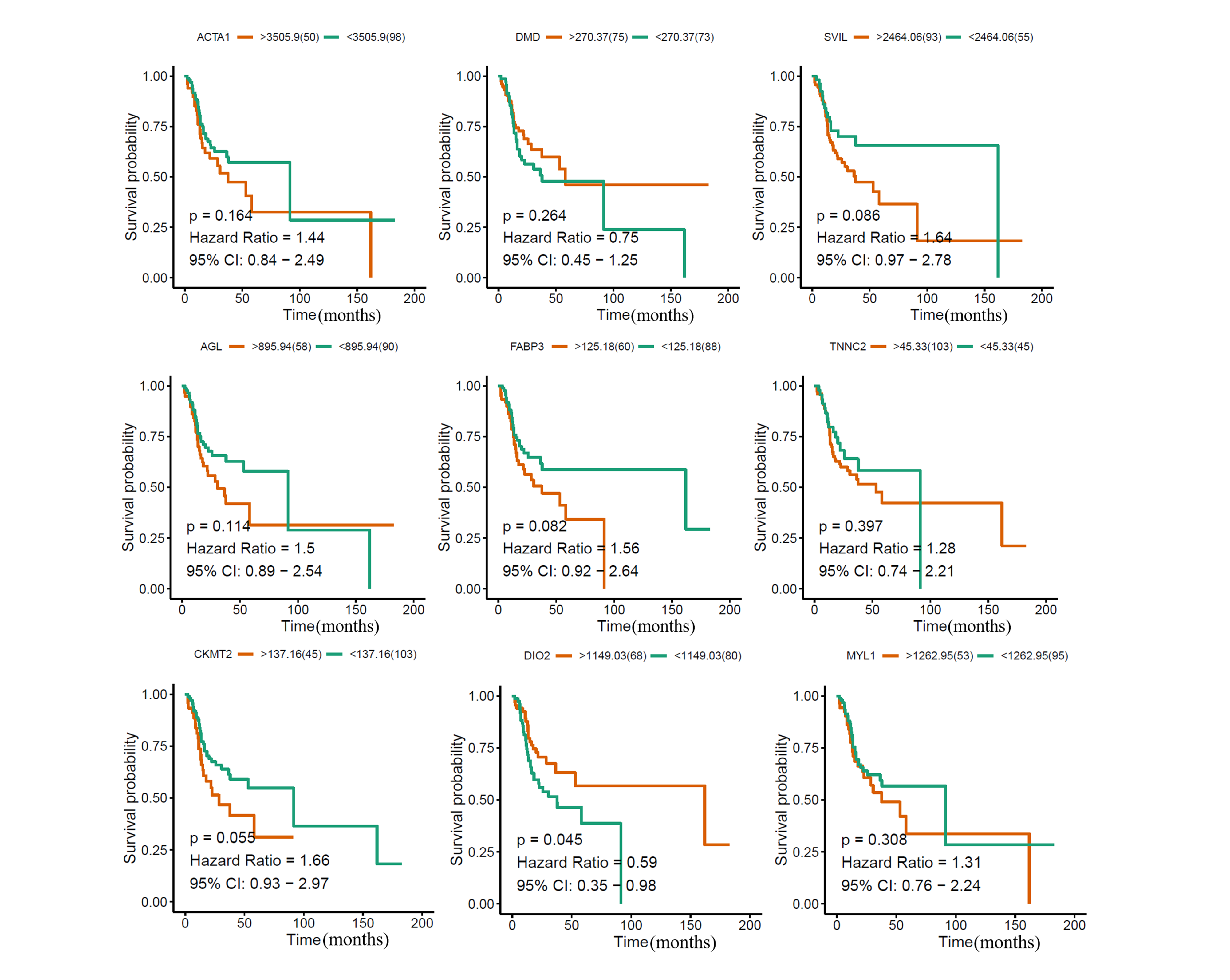

Supplement: Supplementary file 2 — Additional file 2: Figure S1. Overall survival analysis of 9 hub genes of TCGA tongue cancer patients. [file 12935_2019_1028_MOESM2_ESM.tif]
